# Skew *t* Mixture Latent State-Trait Analysis: A Monte Carlo Simulation Study on Statistical Performance

**DOI:** 10.3389/fpsyg.2018.01323

**Published:** 2018-08-02

**Authors:** Louisa Hohmann, Jana Holtmann, Michael Eid

**Affiliations:** Department of Education and Psychology, Freie Universität, Berlin, Germany

**Keywords:** mixture modeling, skew *t*-distribution, latent state-trait analysis, longitudinal data, non-normality

## Abstract

This simulation study assessed the statistical performance of a skew *t* mixture latent state-trait (LST) model for the analysis of longitudinal data. The model aims to identify interpretable latent classes with class-specific LST model parameters. A skew *t*-distribution within classes is allowed to account for non-normal outcomes. This flexible function covers heavy tails and may reduce the risk of identifying spurious classes, e.g., in case of outliers. Sample size, number of occasions and skewness of the trait variable were varied. Generally, parameter estimation accuracy increases with increasing numbers of observations and occasions. Larger bias compared to other parameters occurs for parameters referring to the skew *t-*distribution and variances of the latent trait variables. Standard error estimation accuracy shows diffuse patterns across conditions and parameters. Overall model performance is acceptable for large conditions, even though none of the models is free from bias. The application of the skew *t* mixture model in case of large numbers of occasions and observations may be possible, but results should be treated with caution. Moreover, the skew *t* approach may be useful for other mixture models.

## Introduction

Many psychological theories aim to explain the determinants of behavior and assume influences of the person, the situation and the interaction between person and situation: What persons do depends on their personal disposition as well as their current situation, and individual behavior may change over time (Funder, 2008). In order to distinguish between temporally stable and variable (occasion-specific) components of behavior, a wide range of psychometric models to analyze change has been developed.

Latent state-trait (LST) models (Steyer et al., 1992, 1999, 2015) are structural equation models (SEMs) for longitudinal data. In LST models, there are latent variables representing influences of (a) the person, (b) the situation and the person-situation interaction as well as (c) measurement error (Eid and Luhmann, 2012; Steyer et al., 2015). In contrast to other models for analyzing change (e.g., autoregressive or growth curve models), LST models focus on reversible short-term fluctuations around general dispositions (Khoo et al., 2006).

In the past decades, LST models have been successfully tested and applied in various fields of psychology (for an overview see Geiser and Lockhart, 2012). Furthermore, extensions have been made to include additional features (e.g., autoregressive components; Cole et al., 2005; Eid et al., 2017) or to account for difficult modeling issues, such as population heterogeneity (Courvoisier et al., 2007).

Typically, classical single group SEMs assume that the model parameters are identical for all individuals. However, this assumption might be too strict, as subpopulations with varying degrees of state variability could exist. In some fields of research sources of heterogeneity might be known beforehand, e.g., in case of experimental and control groups, but in other domains the groups are not clearly defined. To deal with population heterogeneity when grouping variables are unknown, mixture SEMs as extensions of single group SEMs have been developed. They are used to identify subpopulations, so called latent classes, with varying model parameters (Jedidi et al., 1997; Lubke and Muthén, 2005). Mixture LST models represent a special variant of mixture SEMs aiming to identify groups with class-specific LST models (Courvoisier et al., 2007): It is assumed that an LST model holds in each class, but the classes differ in their population parameters. For example, a perfectly stable and a highly variable class could exist (Eid et al., 2008).

From a technical viewpoint, observed variables in mixture models are non-normally distributed, if, for example, the variables are normally distributed within classes. The observed distributions represent weighted sums of elementary distributions which are a priori assumed to follow a specific functional form. This assumption determines the number of identified classes (McLachlan and Peel, 2004). Typically, due to computational convenience and mathematical tractability, normal distributions are assumed within classes (Hoeksma and Kelderman, 2006), but this assumption might be wrong if variables are non-normally distributed within classes. In practice, non-normal data is often observed (Micceri, 1989). A technical misspecification can lead to spurious classes and misinterpretations in applications (Bauer and Curran, 2003). Especially in case of outliers, as often observed in applied problems, latent classes might be simply formed to match heavy tails of the underlying distributions even though an additional subpopulation would not be necessary when assuming a different within-class distribution. Interpreting these classes as subgroups of the population would be incorrect and may result in biased effects (Muthén and Asparouhov, 2015).

To deal with this concern, a new method for mixture SEMs based on the restricted skew *t-*distribution has been proposed by Asparouhov and Muthén (2015). Instead of assuming within-class normality, the variables are allowed to follow the more flexible skew *t-*distribution. The standard structural equation modeling framework based on fitting means and covariances is extended, as the new approach additionally accounts for skewness and kurtosis of the data. Thus, the risk of identifying spurious classes due to heavy tails and skewed distributions is reduced.

Good performance of the skew *t* approach in the context of growth mixture models (Muthén and Asparouhov, 2015) suggests that it might be a powerful tool for other mixture models for longitudinal data, such as mixture LST models, as well. Based on theories about possible population heterogeneity with respect to the stability and variability of a given construct, mixture LST models could be used in various contexts in order to investigate whether unobserved subgroups exist. In applications of single group LST models the observed variables often show skewed distributions and outliers (Kenny and Zautra, 1995; Eid et al., 1999; Schmitt, 2000; Eid and Diener, 2004; Schermelleh-Engel et al., 2004; Courvoisier et al., 2007). Thus, by assuming a skew *t-*distribution within classes the underlying processes determining the non-normality could be investigated in more detail: Comparing normal and skew *t* mixture models could help to find adequate and parsimonious explanations for phenomena in various contexts.

The purpose of the current study is to examine the statistical performance of a skew *t* mixture LST model, focusing on realistic conditions in the domain of LST research in order to develop guidelines regarding the model's applicability in practice. Therefore, the design is based on an empirical application and simulation study by Courvoisier et al. (2007) examining a normal mixture LST model. The article is structured as follows: First, the basic ideas of single group and mixture LST models are introduced. Second, the skewed structural equation model (SEM) as introduced by Asparouhov and Muthén (2015) is described. Third, previous findings regarding applications and simulations of mixture SEMs and the skew *t* approach are summarized. Fourth, the Monte Carlo (MC) simulation study on the statistical performance of the skew *t* mixture LST model is reported, and finally the findings are discussed.

## Models

LST models are longitudinal models with different latent variables representing time-stable and occasion-specific aspects of behavior as well as measurement error.

### General single group LST model

Generally, an observed variable *Y*_*ij*_ representing an indicator *i* on occasion *j* is decomposed into a latent state variable *S*_*ij*_ and an error variable *E*_*ij*_.

(1) $$\begin{array}{r} Y_{ij}=S_{ij}+E_{ij}. \end{array}$$

The measurement error-free (true) values on *S*_*ij*_, characterizing an individual in a specific situation, can be further decomposed into a latent trait variable *T*, the stable part across occasions, and an occasion-specific variable *O*_*j*_, the deviation of the momentary state from the person-specific trait:

(2) $$\begin{array}{r} Y_{ij}=\alpha_{ij}+\lambda_{Tij}T+\lambda_{Oij}O_{j}+E_{ij}. \end{array}$$

**Figure 1:**
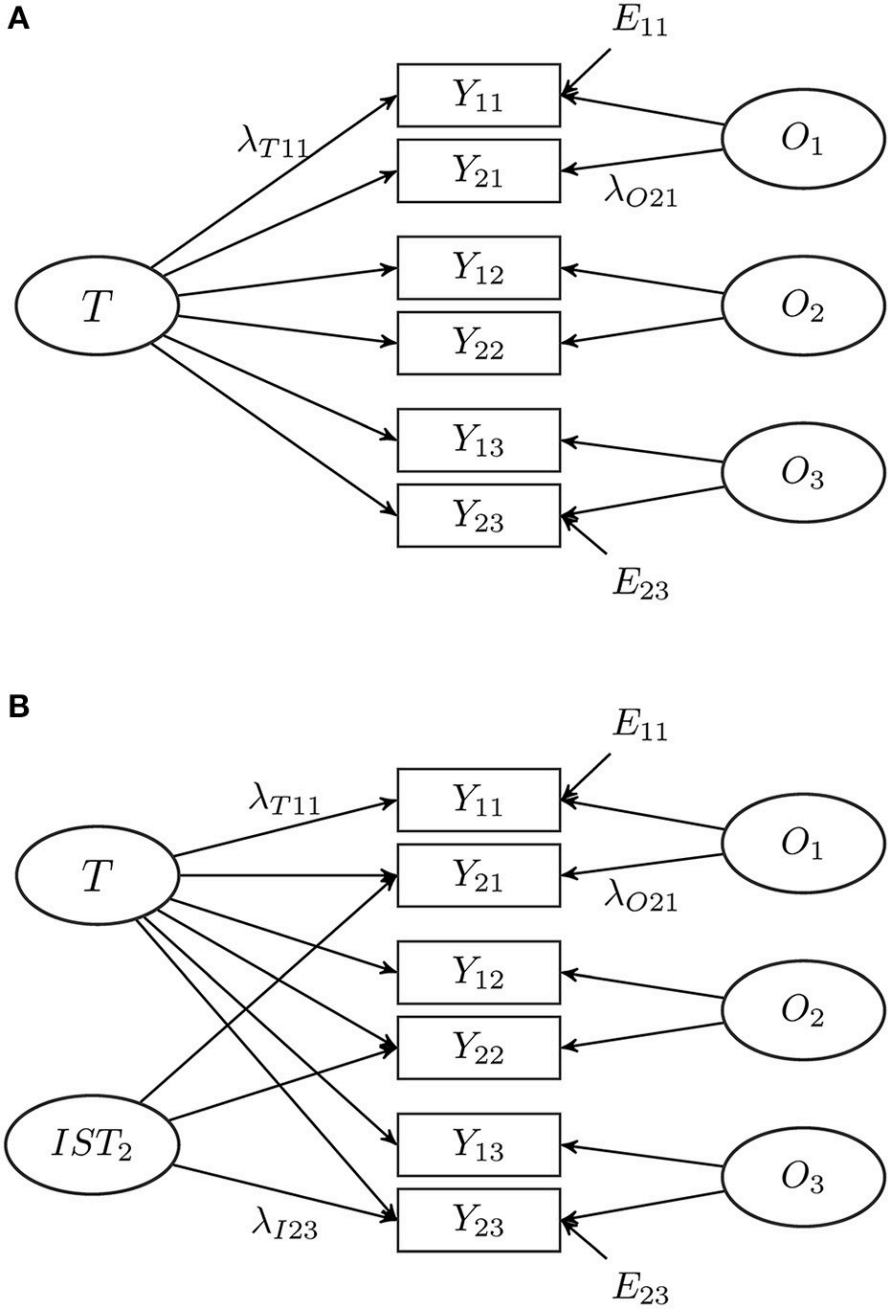
Examples of latent state-trait (LST) models with two indicators (*i* = 1, 2) and three occasions of measurement (*j* = 1, 2, 3). **(A)** Single-trait LST model; **(B)** LST model with an indicator-specific residual trait factor. *E*_*ij*_ = error variables; *IST*_2_ = latent indicator-specific trait residual variable for the second indicator; *O*_*j*_ = latent occasion-specific variable; *T* = latent trait variable; *Y*_*ij*_ = observed variable; λ_*Tij*_, λ_*Oij*_ and λ_*Iij*_ = loading parameters.

In this equation, it is assumed that the latent trait variables *T* and the occasion-specific variables *O*_*j*_ are perfectly correlated across the different indicators, where the intercept α_*ij*_ and the loading parameters λ_*Tij*_ and λ_*Oij*_ are real constants. To ensure identifiability some restrictions with respect to their possible values have to be made. For this simulation study, these restrictions and additional invariance settings are described below in the Methods section. A single group LST model for three occasions of measurement is depicted in Figure 1A.

The occasion-specific variables *O*_*j*_ are random fluctuations around the trait and are defined as residuals with respect to *T*, so that their expectation is zero (Steyer et al., 1999, 2015). Furthermore, the *O*_*j*_ are uncorrelated with each other. Moreover, in the model depicted in Figure 1A, correlations between *T* and *O*_*j*_, *T*, and *E*_*ij*_, as well as *O*_*j*_ and *E*_*ij*_ are zero and the *E*_*ij*_ are uncorrelated with each other.

### LST model with a common trait and indicator-specific trait residual factors

To apply SEMs, multiple indicators *i* per construct are necessary, for example different items, physiological measures or different raters. From many empirical applications it is known that different indicators representing the same construct might not be unidimensional: They may contain a unique part not shared by the other indicators. Due to these indicator-specific parts, indicators are assumed to be more highly correlated with themselves than with different indicators over time (Eid et al., 1999; Geiser and Lockhart, 2012).

To account for the heterogeneity of indicators, an LST model with a common trait and an indicator-specific trait residual factor was introduced by Eid and Diener (2004). In contrast to other models including indicator-specific influences (e.g., an LST model with indicator-specific traits; Eid, 1996), it includes a common trait, assuming that there is a general disposition underlying all indicators. This specification can have advantages with respect to the interpretation of the latent variables. Furthermore, it can avoid improper solutions (e.g., correlations >1) that could occur in LST models with indicator-specific traits when the trait variables are highly correlated (Courvoisier, 2006). In this model, an observed variable *Y*_*ij*_ with *i* > 1 is decomposed as follows:

(3) $$\begin{array}{r} Y_{ij}=\alpha_{ij}+\lambda_{Tij}T+\lambda_{Iij}IST_{i}+\lambda_{Oij}O_{j}+E_{ij}\;\text{for}i>1 \end{array}$$

Observed variables *Y*_*ij*_ with *i* = 1 are decomposed according to Equation (2). For all indicators with *i* > 1, an indicator-specific trait residual factor *IST*_*i*_ with a loading parameter λ_*Iij*_ is added in Equation (3). It represents parts of the indicators with *i* > 1 that are not shared with the first (reference) indicator. As *IST*_*i*_ are defined as residuals with respect to *T*, their expectation is zero. For example, positive values of *IST*_2_ reflect that the response on the second indicator (*i* = 2) is higher than expected based on the answer of the first indicator (*i* = 1). The interpretation of the other latent variables is not different from their interpretation in the general LST model. An LST model with a common trait, one indicator-specific trait residual factor, two indicators per occasion (*i* = 1, 2), and three occasions of measurement (*j* = 1, 2, 3) is depicted in Figure 1B.

In single group LST models these coefficients are assumed to be the same for the entire population. However, describing the population with a single set of parameters might be oversimplifying if structural differences between (unobserved) subgroups exist (Lubke and Muthén, 2005). In the following, a mixture LST model as a special variant of mixture SEMs accounting for unobserved population heterogeneity is described.

### Mixture LST models as extensions of mixture models and LST models

Mixture models represent a flexible method of modeling complex phenomena and have received attention from a theoretical as well as a practical perspective (McLachlan and Peel, 2004). The mixture LST model is a special case of mixture SEMs. Mixture models can be viewed as multigroup SEMs with unobserved and unknown grouping variables (Muthen, 2001). Thus, the application of mixture SEMs is useful if subpopulations are assumed but variables related to class affiliation are not easily measured and/ or the underlying processes are not clear (Ram and Grimm, 2009).

Mixture LST models combine the ideas of individual and structural differences in intraindividual variability: Because of the LST component of the model, interindividual differences in the expected observed variability are allowed. Additionally, because of the mixture component of the model, structural differences between subgroups are possible as the model parameters can differ between classes. Thus, to define the general mixture LST model for each latent class *c*, Equation (3) is extended to:

(4) $$\begin{array}{r} Y_{ijc}=\alpha_{ijc}+\lambda_{Tijc}T_{c}+\lambda_{Iijc}IST_{ic}+\lambda_{Oijc}O_{jc}+E_{ijc}, \end{array}$$

with probability π_*c*_ of belonging to a specific class. The interpretation of the components remains the same within classes. In order to identify the model, the same restrictions have to be made within classes as for a single group LST model (Courvoisier et al., 2007).

Applications of mixture models should be based on theories about population heterogeneity in order to retrieve interpretable results (Bauer and Curran, 2003). Many psychological theories deal with individual and structural differences in intraindividual variability (Molenaar et al., 2003; Molenaar and Campbell, 2009; Kuppens et al., 2010; Brose et al., 2012; Röcke and Brose, 2013). Different trait variances between latent classes observed in mixture LST models may for example differentiate “traited and untraited individuals” (Baumeister and Tice, 1988). Furthermore, mixture modeling is considered as a promising tool with respect to psychiatric research (Miettunen et al., 2016). By applying mixture LST models in this context, for example subgroups with varying patterns of stable or variable psychiatric symptoms could be detected.

### The skew *t* structural equation model

The number of identified classes in mixture models is determined by the functional form of the observed and latent variables' distributions assumed within classes. Typically, normal distributions are used due to feasibility and computational advantages (Dolan and van der Maas, 1998). An important disadvantage of this assumption is that in case of strongly non-normal outcome variables, latent classes might be formed to cover skewness and/ or heavy tails of the distribution. Thus, there are ongoing debates about whether small classes should be seen as subpopulations or technical devices (McLachlan and Peel, 2004). Relaxing the assumption of within-class normality and assuming another, more flexible functional form instead may reduce the risk of identifying classes without substantive meanings (Muthén and Asparouhov, 2015).

From a practical point of view, assuming skew *t*-distibutions is reasonable in different contexts: Not only for biological variables (e.g., fluorphorelabeled antibodies or flow cytometric data; Ho et al., 2012) and physical and hematological measurements (e.g., BMI, height and body fat; Lee and McLachlan, 2014; Muthén and Asparouhov, 2015), but also for psychological variables (e.g., fatigue; Ho et al., 2014; perceived severity of uisng steroids; Asparouhov and Muthén, 2015; cogniitve abilities and depression; Muthén and Asparouhov, 2014) or economic variables (e.g., excess rates; Arellano-Valle and Azzalini, 2013).

Asparouhov and Muthén (2015) introduced an SEM framework, namely “skewed structural equation models” (p. 1), based on the skew *t*-distribution that can also be applied to mixture SEMs. They use the restricted skew *t*-distribution, since it allows for explicit maximum likelihood (ML) estimation. A multivariate *p-*dimensional variable ***Y*** following this flexible functional form is denoted by

(5) $$\begin{array}{r} \text{Y}\sim rMST\left(\mu ,\Sigma ,\delta ,\nu \right). \end{array}$$

In this Equation, **μ** represents a *p* × 1 location vector, **Σ** equals a *p* × *p* scale matrix, **δ** reflects a *p* × 1 skewness vector, and ν is a positive degrees of freedom parameter. Thus, a multivariate skew *t-*distribution has *p*+1 more parameters compared to a multivariate normal distribution: For each variable a parameter **δ** is added indicating the skewness of the distribution to the left (positive values) or the right (negative values). Furthermore, the degrees of freedom parameter ν reflects the deviation from normality in terms of the thickness of the tails. This parameter is the same for all variables. Generally, values of ν < 3 are only recommended for distributions with substantially heavy tails (Lee and McLachlan, 2014; Muthén and Asparouhov, 2015).

The multivariate restricted skew *t*-distribution encompasses a multivariate *t*-distribution in the case of **δ** = 0. Furthermore, it is reduced to a multivariate skew-normal distribution when ν is fixed to a large value, i.e., 10,000. As a third special case, the multivariate normal distribution is given when fixing both **δ** to zero and ν to a large value (Muthén and Asparouhov, 2015).

For a skew *t* SEM, only *p* skewness parameters δ can be identified (Muthén and Asparouhov, 2015). In this simulation study, the *T*_*c*_ follow a skew *t*-distribution whereas for the other latent variables normal distributions are maintained. Thus, the skewness of the observed variables is explained by the skewness of the general disposition. The *O*_*jc*_ and *IST*_*ic*_ are defined as residuals with respect to *T*_*c*_, i.e., unstructured fluctuations around the general disposition, and are assumed to be normally distributed.

Thus, the class-specific trait factors *T*_*c*_ are expressed as:

(6) $$\begin{array}{r} T_{c}=\mu_{Tc}+\;\xi_{Tc} \end{array}$$

with μ_*Tc*_ representing the location parameter of *T*_*c*_ and,

(7) $$\begin{array}{r} \xi_{Tc}\;\sim \;rMST\left(0,\sigma_{Tc}^{2},\delta_{Tc},\nu_{c}\right), \end{array}$$

where 0 is the zero location parameter for $\xi_{Tc},\;\sigma_{Tc}^{2}\;$is the scale parameter of ξ_*Tc*_, δ_*Tc*_ is the within-class skewness parameter for ξ_*Tc*_ and ν_*c*_ is the degrees of freedom parameter.

Muthén and Asparouhov (2015) describe advantages of the skew *t* approach for SEMs in general and specifically for mixture models: Fitting this more flexible form to the data generally allows extracting more (higher-order) information, i.e., not only means and covariances but also skewness and kurtosis. The skew *t* models can provide better model fit than normal models as they account for imperfections of reality, for example, nonlinearity, to a greater degree. Compared to skew-normal distributions it is possible to handle larger skewness, i.e., |δ| > 1, of the data (Asparouhov and Muthén, 2015; Muthén and Asparouhov, 2015). Thereby, regarding mixture modeling, more parsimonious models can be identified and the risk of spurious classes is reduced compared to normal mixture SEMs: Applying the skew mixture SEM, within-class distributions are allowed to be skewed and include heavy tails. Thus, additional classes at the ends of the distribution to account for non-normality are not formed. Subpopulations identified by a skew mixture SEM can be more meaningfully compared to classes of normal mixture SEMs. Furthermore, the stability and reproducibility of a normal mixture solution can be checked as the restricted skew *t*-distribution encompasses the *t-*distribution, the skew-normal distribution and the normal distribution as special cases (Asparouhov and Muthén, 2015; Muthén and Asparouhov, 2015).

Nevertheless, disadvantages of the skew *t* approach should be considered as well: Computation times are larger as compared to the normal approach, larger sample sizes and more random start values are needed in applications. Classification entropy often appears to be lower. Additionally, the method is restricted to continuous variables and Likert scales may not contain enough information to estimate **δ** and ν (Asparouhov and Muthén, 2015; Muthén and Asparouhov, 2015).

## Previous research

### Applications of mixture models

Mixture models have been frequently applied in the past several years. GMMs have been used in order to identify subpopulations in the longitudinal change of various variables such as cortisol stress response (Ram and Grimm, 2009; Koss et al., 2013), drug, alcohol or tobacco use (Colder et al., 2001; Li et al., 2001; Hix-Small et al., 2004; Greenbaum et al., 2005), psychiatric symptoms (Stoolmiller et al., 2005; Armour et al., 2012; Hallquist and Lenzenweger, 2013), intelligence (Morgan and Beaujean, 2014), cognitive abilities (Espy et al., 2009), aggression (Brame et al., 2001; Petras et al., 2004), subjective well-being (Pinquart and Schindler, 2007; Li and Hser, 2011), prevalence of delinquent behavior (Reinecke, 2006), or sexual functioning (Legler et al., 2004). Furthermore, mixture SEMs assuming more complex relationships between several latent variables have been applied in various contexts as well, for instance psychopathology (Jonas and Markon, 2013; Litson et al., 2017), mood (Courvoisier et al., 2007), treatment effects (Peugh et al., 2017), cognitive abilities (McArdle and Prindle, 2008; Van Horn et al., 2009; Brandt et al., 2015), achievement goals (Wang et al., 2010), personality and life satisfaction (McIntyre, 2011; Heidemeier and Göritz, 2016), customer satisfaction (Jedidi et al., 1997; Herrmann et al., 2002), or job satisfaction (Lee and Song, 2003). Compared to the large body of GMMs, other mixture SEMs have been used to a lesser degree. Recently, mixture LST models have been examined solely by Courvoisier et al. (2007) with an application in the context of mood assessment.

Typically, within-class normality was assumed in previous studies of mixture SEMs, and some authors used log transformations to normalize the variables prior to the analysis (Lin et al., 2002; Wiesner and Windle, 2004; Greenbaum et al., 2005; Bollen and Curran, 2006; McArdle and Prindle, 2008). The skew *t* approach has not been frequently applied yet. Muthén and Asparouhov (2015) analyzed a GMM for body-mass index (BMI) with normal or skewed distributions within classes. With the skew *t*-distribution a more parsimonious model with two instead of three classes could be identified. In another study investigating longitudinal changes in purpose of life, a one class skew *t* growth curve model showed better model fit as compared to two- or three-class normal GMMs (Ko et al., 2016). Furthermore, Kooken (2015) examined the longitudinal growth of student classroom behavior with skewed observed variables. In this study, the skewed approach produced a non-positive definite covariance matrix and, therefore, a normal GMM was applied instead.

### Simulation studies for mixture SEMs

A large number of simulation studies in the context of mixture modeling has been conducted on various topics, e.g., performance of model selection criteria (Jedidi et al., 1997; Lee and Song, 2003; Brame et al., 2006; Courvoisier et al., 2007; Henson et al., 2007; Nylund et al., 2007; Kim, 2014; Morgan and Beaujean, 2014; Usami, 2014), class enumeration (Lubke and Tueller, 2010; Liu, 2011; Martin and von Oertzen, 2015; Diallo et al., 2016), parameter estimation (Jedidi et al., 1997; Lee and Song, 2003; Courvoisier et al., 2007; Lubke and Muthén, 2007; Tolvanen, 2007; Tueller and Lubke, 2010), class assignment (Lubke and Tueller, 2010), confidence intervals (Dolan and van der Maas, 1998), covariate inclusion (Li and Hser, 2011; Kim et al., 2016), missing data (Lee and Song, 2003; Gottfredson et al., 2014), local solutions (Hipp and Bauer, 2006), label switching (Tueller et al., 2011) or comparison with other approaches (Martin and von Oertzen, 2015).

With respect to parameter estimation accuracy, different influence factors were identified: Tolvanen (2007) reported positive effects of the degree of differences between latent classes, sample size and reliability of the observed variables on the performance of a two-class GMM. Tueller and Lubke (2010) identified combined influences of sample size, class separation, effect size, response format, proportion of class sizes and differences in factor variances for mixture SEMs. Within the framework of two-class factor mixture models, greater class separation was associated with better coverage, and also allowing class-specific parameters influenced statistical performance (Lubke and Muthén, 2007). So far only one simulation study (Courvoisier et al., 2007) was conducted for a two-class mixture LST model with within-class normality. Parameter estimation performance and behavior of the adjusted likelihood ratio test (aLRT; Lo et al., 2001) were examined. Parameter estimation efficiency and accuracy increased with increasing numbers of observations and occasions. The authors concluded that models with at least four measurement occasions and 250 observations yield satisfactory parameter estimates. Furthermore, the aLRT was powerful even in case of small sample sizes (*N* = 125).

For the skew *t* approach in mixture modeling only one simulation study examined parameter estimation and the choice of the correct number of classes in comparison to the normal approach (Muthén and Asparouhov, 2015). For *N* = 2,000, the skew *t* GMM always pointed to the correct two-class solution whereas the performance of the normal GMM was worse (88% correct identification). Furthermore, parameter and standard error (SE) estimation accuracy were high for the key parameters of the skew *t* GMM, namely class-specific means, skewness, degrees of freedom and the logit parameter of belonging to the first class (referring to the class size). The authors reported that results for *N* = 1,000 were good as well. Moreover, Asparouhov and Muthén (2015) conducted a simulation study on the performance of the skew approach for models with latent variables in which they examined a single group factor analysis model with a skew-normal distribution (*N* = 5,000). Again, the skew model performed better than a normal model, with low parameter estimation bias and high coverage. In this model, not only the factor was assumed to follow a skewed distribution, but also one indicator variable. Based on their results, Asparouhov and Muthén (2015) pointed out that robust ML estimation may not deal with non-normality in case of complex relationships between latent variables. These results indicate that applying the skew *t* approach might be of advantage in many cases, however, there is still need to evaluate its performance. The authors did not vary potential influence factors. For instance, larger degree of skewness and sample sizes were found to positively influence the performance of a skew *t* exploratory factor analysis model (Lin et al., 2015). Due to the distributional similarities between the skew *t* factor analysis model and the skew *t* mixture model, the performance of the latter may vary depending on sample size and skewness as well.

Previous results of skew *t* mixture models indicate that there is still need to evaluate the statistical performance of these models in order to identify conditions under which adequate results can be expected. Mixture models can vary greatly with various different specifications within and across classes (McLachlan and Peel, 2004). As the aim of this study is to investigate the mixture LST model under realistic conditions, the simulation was designed closely to Courvoisier et al. (2007) examining the same model type. Sample size and number of occasions were considered as potential influence factors. Furthermore, different degrees of skewness were investigated according to results by Lin et al. (2015). Better model performance is hypothesized for larger sample sizes, number of occasions and skewness.

## Method

### Data generation and population model

As a general population model the LST mixture model with one common trait, two indicators per occasion, one indicator-specific trait residual factor and two latent classes as examined by Courvoisier et al. (2007) was used. In addition to an empirical application in the context of mood analysis, these authors conducted a MC simulation study for the normal mixture LST model that fitted their data best. To simulate realistic values in the research domain of LST models, the same model parameters were chosen for the present MC simulation study.

In contrast to a normally distributed variable, for a skew *t*-distributed variable the parameter μ not simply equals the mean and the parameter σ^2^ not simply equals the variance, and modeling mean, variance and skewness is not independent from each other. Means, variances and univariate skewness values for variables following a skew *t-*distribution can be calculated using formulas based on μ, Σ, δ and ν presented in Asparouhov and Muthén (2015). For this simulation, μ_*Tc*_ (the location parameter) and $\sigma_{Tc}^{2}$ (the scale parameter) equal the class-specific means and variances of the trait factors in the simulation of Courvoisier et al. (2007). In line with Asparouhov and Muthén (2015), in this simulation study for the skewed trait variable results are presented for μ_*Tc*_, $\sigma_{Tc}^{2}$ and δ_*Tc*_. For the normally distributed latent variables, results are presented for the variances which equal the scale parameters.

Due to identifiability reasons the following restrictions have to be made within each class:

- A minimum of one loading parameter per factor has to be fixed to a positive value (typically 1).
- For factors with only two indicators that are uncorrelated to all other factors (*O*_*jc*_ in this design), both loadings have to be fixed.
- For the residual factors (*IST*_2*c*_ and *O*_*jc*_ in this design) the means are fixed to zero.
- One intercept α_*ijc*_ has to be fixed to zero to identify the mean of the common latent trait variable *T*_*c*_.

Class sizes were set to 76 and 24% and in the following the first class is considered to be the larger one (referring to a logit parameter of belonging to Class 1 of 1.15). In line with Courvoisier et al. (2007), some parameters were held equal across time within each class: Measurement invariance (MI) with respect to the mean structure, loadings, and occasion-specific variances was assumed. Thus, intercepts, α_*ijc*_ = α_*ic*_, loading parameters, λ_*Tijc*_ = λ_*Tic*_, λ_*Iijc*_ = λ_*Iic*_ and λ_*Oijc*_ = λ_*Oic*_, variances of the occasion-specific factors, *Var*(*O*_*jc*_) = *Var*(*O*_*c*_) for all *j*, and residual variances of the observed variables for *j* > 1, were set equal across time. Therefore, the identifiability restrictions and MI settings resulted in loadings λ_*T*1*c*_, λ_*I*2*c*_, λ_*O*1*c*_ and λ_*O*2*c*_ fixed to 1. Furthermore, the intercept of the first indicator α_1*c*_was fixed to 0. With respect to the error variances, Courvoisier et al. (2007) observed a so called *Socratic effect* (Jagodzinski et al., 1987), i.e., a larger error variance for the first as compared to the other occasions, that was also modeled. Apart from this effect, the MI restrictions regarding intercepts, loadings and residual variances reflect strict MI (Meredith, 1993). Furthermore, the residual variances were the same for both indicators. Equal intercepts and loading parameters across occasions ensure that the same construct is measured over occasions (Eid and Kutscher, 2014).

In addition to MI across time, the classes were allowed to differ in the following parameters (The index *c* indicates class-specific values):

- The loadings of the second indicator λ_*T*2*c*_ on the latent trait variable *T*_*c*_,
- the intercepts of the second indicator α_2*c*_,
- the location parameters of the latent trait variables μ_*c*_,
- the scale parameters of the latent trait variables $\sigma_{Tc}^{2}$,
- the variances of the latent indicator-specific trait residual variables *Var*(*IST*_2*c*_),
- the variances of the latent occasion-specific variables *Var*(*O*_*c*_),
- and the residual variances at the first occasion *Var*(*E*_1*c*_) and at the following occasions *Var*(*E*_*jc*_), *j* > 1.

Exact population parameters used in the simulation study are presented in Table S1 in the Supplementary Material. The larger (first) class is characterized by relatively lower average trait values, larger trait variances, and greater interindividual differences in occasion-specific fluctuations. Influences of measurement error (residual variances) are larger as compared to the second class as well.

**Figure 2:**
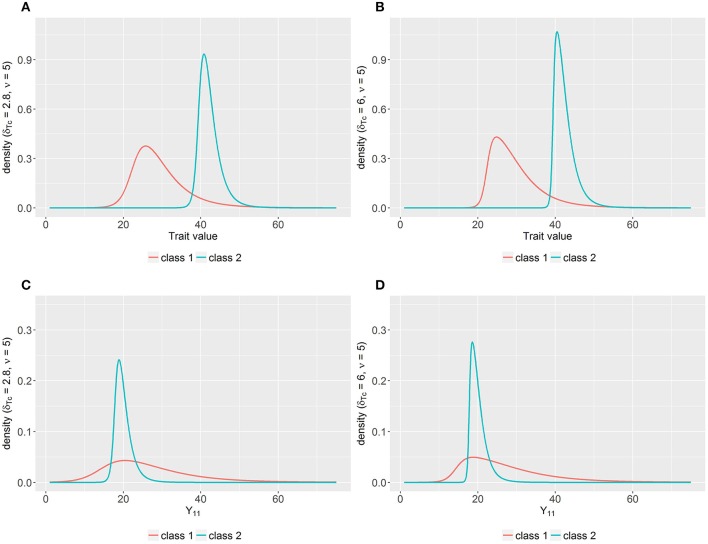
**(A)** Model-implied distribution of the trait variables per class for δ_*Tc*_ = 2.8 and ν_*c*_ = 5; **(B)** Model-implied distribution of the trait variables per class for δ_*Tc*_ = 6 and ν_*c*_ = 5; **(C)** Model-implied distribution of the observed variable *Y*_11_ per class for δ_*Tc*_ = 2.8 and ν_*c*_ = 5; **(D)** Model-implied distribution of the observed variable *Y*_11_ per class for δ_*Tc*_ = 6 and ν_*c*_ = 5; δ_*Tc*_ = class-specific skewness parameter for the trait factor; ν_*c*_ = class-specific degrees of freedom parameter; *Y*_11_ = observed variable for the first indicator at the first measurement occasion.

The common trait was simulated following a skew *t*-distribution with ν_*c*_ = 5 (based on Muthén and Asparouhov, 2015) and different skewness parameters δ_*Tc*_ depending on the simulation conditions as described below. In line with Muthén and Asparouhov (2015), these parameters were equal for both classes, but estimated separately so that results are presented for class-specific parameters. The model-implied distributions of the class-specific trait variables for the two skewness conditions and corresponding distributions of an observed variable (*Y*_11_) are depicted in Figure 2.

### Simulation design

In this simulation study, the following aspects were manipulated, resulting in 4 × 5 × 2 = 40 conditions:

- sample size (*N* = 125, 250, 500, 1,000),
- occasions of measurement (*j* = 2, 3, 4, 5, 6), and
- skewness (δ_*Tc*_ = 2.8, 6).

Sample sizes and occasions of measurement represent realistic numbers in the research domain of LST models (Geiser and Lockhart, 2012) and are based on Courvoisier et al. (2007). Sample sizes are within the “typical range of values” (p. 637) proposed by Bandalos (2013). The values for δ_*Tc*_ represent *mild* and *high* skewness parameters, as also used in other simulation studies (Wall et al., 2012; Lin et al., 2015). In line with Courvoisier et al. (2007) and Muthén and Asparouhov (2015) 500 replications were used.

All models were estimated with Mplus version 7.3 (Muthén and Muthén, 1998-2007) and analyzed using the software R (R Core Team, 2015). A sample input is provided in the Supplementary Material. Similar to Muthén and Asparouhov (2015) the default Mplus settings with respect to the number of iterations, and convergence criteria were used (Muthén and Muthén, 1998-2007). In the estimation of mixture models multiple optima of the likelihood function can occur (Hipp and Bauer, 2006). In line with other simulation studies on mixture models (Courvoisier et al., 2007; Muthén and Asparouhov, 2015) the population values were chosen as starting values in order to reduce related problems and computation time. This can also diminish the risk of label switching (Tueller et al., 2011) as described below.

### Evaluation criteria

The performance of the skew *t* models was judged regarding the following criteria:

- the rate of non-convergence after 500 iterations,
- the amount of parameter estimation bias (*peb*),
- the amount of standard error bias (*seb*),
- the mean squared error (*MSE*),
- and the 95 percent coverage of the data generating value.

In order to assess the accuracy of parameter estimation the *peb* was calculated per parameter. It represents the estimated model parameter's deviation from the population value, relative to the population value. Across replications it was calculated by

(8) $$\begin{array}{r} peb\left(\theta_{p}\right)=\frac{1}{n_{r}}\cdot \overset{n_{r}}{\underset{r=1}{\sum }}\frac{{\hat{\theta }}_{pr}-\theta_{p}}{\theta_{p}}, \end{array}$$

with θ_*p*_ denoting the population parameter, ${\hat{\theta }}_{pr}$ representing the estimate for model parameter *p* in replication *r*, and *n*_*r*_ representing the number of replications considered for the calculation.

Similarly, the *seb* was used to measure the accuracy of standard error (SE) estimation for a parameter. It represents the difference between an estimated sample SE from its population value relative to the population value and was calculated by

(9) $$\begin{array}{r} seb\left(\theta_{p}\right)=\frac{1}{n_{r}}\cdot \overset{n_{r}}{\underset{r=1}{\sum }}\frac{{SE_{\hat{\theta }}}_{pr}-SD_{p}}{SD_{p}}. \end{array}$$

While ${SE_{\hat{\theta }}}_{pr}$ denotes the SE of the estimate $\hat{\theta }$ for parameter *p* in replication *r*, *SD*_*p*_ represents the population value for the SE, that is, the standard deviation (SD) of the parameter estimates over all replications of the specific condition (Bandalos, 2013).

In line with Muthén and Muthén (2002) and Holtmann et al. (2016), absolute values for *peb* and *seb* below the threshold of 0.1, i.e., less than 10% relative deviation from population value, were considered acceptable. Values between 0.1 and 0.3 were considered as medium bias (10–30% relative deviation from population value) and values above 0.3 as large bias (more than 30% relative deviation from population value). In addition to the average values calculated by Equations (8) and (9), distributions of *peb* and *seb* based on the values per replication were inspected using boxplot diagrams.

Both low bias and low variation of the estimates across replications are desirable. In order to account for a possible trade-off between bias and variance, the *MSE* was calculated. Small values indicate small bias in combination with small variation of the estimates across replications. Because the *MSE* depends on the scale, comparisons between conditions were obtained instead of interpreting the absolute values in relation to a specific threshold (Muthén and Muthén, 1998-2007; Courvoisier, 2006; Wall et al., 2012).

The 95% coverage represents the proportion of replications for which the 95% confidence interval (CI) around the mean of the estimated parameter includes the population value. It should be near 0.95. Values between 0.91 and 0.98 were considered acceptable (Muthén and Muthén, 2002).

### Label switching

In simulation studies with mixture modeling, the problem of label switching can occur: The models are only identified up to a permutation of the class labels, i.e., in replications influenced by label switching, the classes swap their labels. With respect to this simulation study, this means that “Class 2” becomes the larger class with parameter estimates matching the values for Class 1 in the population model and vice versa (Dolan et al., 2005). Label switching can lead to incorrect conclusions regarding the means and SDs of parameter estimates so that it is necessary to control for it. Even though it can be prevented to some extent by providing the true population values as starting values for parameter estimation, it is still necessary to check the data after the simulation has been conducted (Tueller et al., 2011).

As the class assignment has to be sufficiently accurate to detect label switching (Tueller et al., 2011), an accuracy criterion for class proportions (Tueller et al., 2011) was applied. All replications with class proportions between 40% and 60% for the first class were excluded. Furthermore, all replications with class proportions of less than 40% for the first class were inspected in order to assess whether the labels may have switched. This criterion referred to a total of 29 replications. As the population values of the classes mostly differed with respect to the mean values of the trait and the variances of the occasion-specific factors, two criteria were applied to decide whether class labels have to be changed: a lower $\sigma_{T}^{2}$ in combination with a higher μ_*T*_ for the first as compared to the second class. In two cases labels were changed. Three replications fulfilled only one of these criteria and were excluded from further analyses as it could not be properly decided whether label switching occurred or not. The other 24 replications fulfilled none of the criteria and were further checked for estimation issues (see below).

## Results

### Convergence

In total, a large number of replications (98.89%; 19,778 out of 20,000) converged after 500 iterations. Increasing the number of iterations did not result in enhanced convergence rates. Generally, convergence was higher for models with larger numbers of observations and occasions. For the normal mixture LST model, Courvoisier et al. (2007) reported smaller convergence rates in small conditions (*j* = 2, 3 and *N* = 125, 250), whereas for the other models convergence rates in the case of normality were at least as high as for the skew *t* models.

Even though convergence was high for the skew *t* approach, for many replications estimation issues occurred. Statistical performance was examined solely for replications with clearly separated classes and without warnings given by Mplus. As a consequence, all of the 29 replications checked for label switching were excluded from the analysis due to warning messages.

With respect to the warnings, in a large number of replications single or multiple parameters were fixed by Mplus to avoid singularity of the information matrix. Detailed numbers of fixed parameters per parameter and condition are given in Figure S1 in the Supplementary Material. Mostly, $\sigma_{Tc}^{2}$ or *Var*(*IST*_2*c*_) and in some cases the residual variances were fixed. For models with few occasions (*j* = 2, 3), some *Var*(*O*_*c*_) were fixed as well. As the values were typically set to zero, including them would have greatly influenced the calculation of the evaluation criteria. Other warnings referred to problems with SE estimation due to a non-positive definite first-order derivate product matrix, low ν_*c*_ estimates^1^ or saddle points in the model estimation.

Regarding class separation, all replications with class proportions between 40% and 60% for the first class (based on the criterion of Tueller et al., 2011) were excluded. In these models it remained unclear which of the classes was the first or second class so that label switching could not be detected.

**Figure 3:**
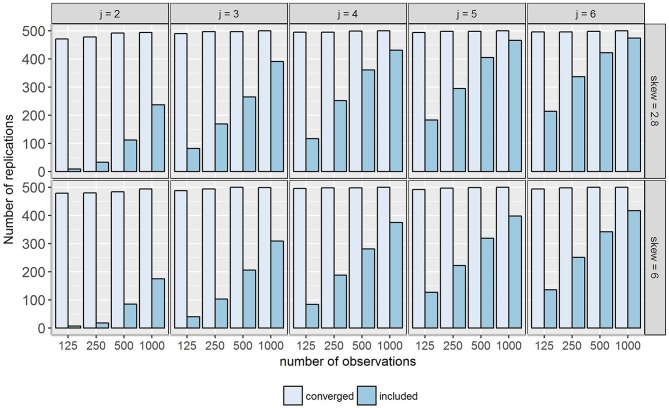
Number of converged and included replications in the simulation study. Included were replications without warning messages and with clear class separation, i.e., the larger class contains at least 60% of the observations. *j* = number of occasions; skew = class-specific skewness parameter for the trait factor (δ_*Tc*_).

Figure 3 shows the amount of converged and included replications. The exact numbers of included and excluded replications per model fulfilling aforementioned criteria are given in Table S2 in the Supplementary Material. After exclusion, only 47.2% of the converged replications (9,338 out of 19,778) remained in the analysis. Thus, all results presented hereafter should be interpreted considering the inclusion rates. As results of conditions with fewer observations and occasions are based on smaller proportions of replications, there is less confidence in the findings compared to other conditions (visualized by error bars representing standard errors in the figures). Comparisons between the skew *t* method and the normal method examined by Courvoisier et al. (2007) were not adequate due to the different numbers of included replications.

For some conditions less than 50 replications remained. This number appeared not to be sufficient for conclusions about statistical performance so that the following five conditions were excluded from further analyses:

- δ_*Tc*_ = 6, *j* = 2, *N* = 125 and *N* = 250,
- δ_*Tc*_ = 6, *j* = 3, *N* = 125,
- δ_*Tc*_ = 2.8, *j* = 2, *N* = 125 and *N* = 250.

**Figure 4:**
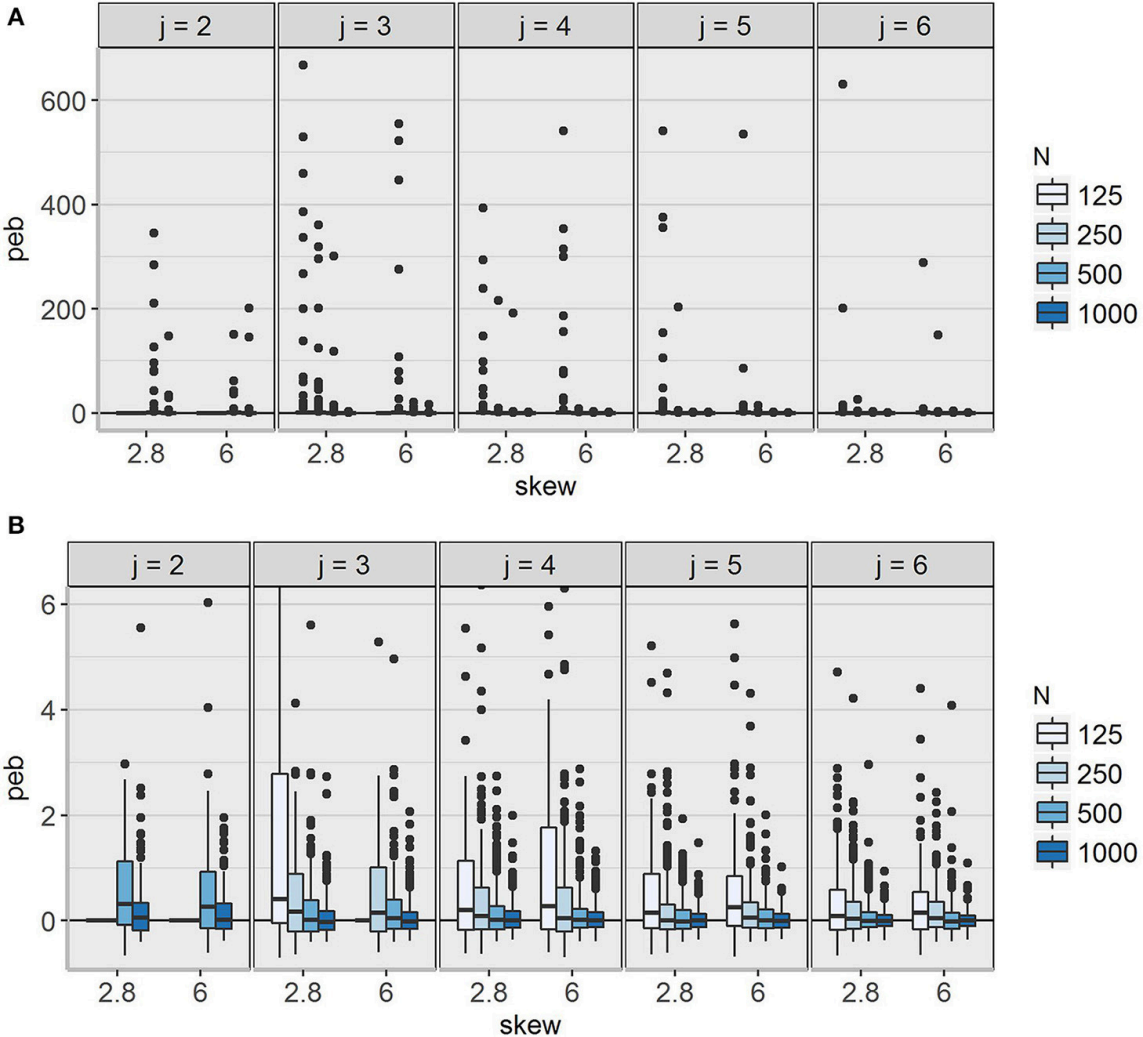
Distribution of parameter estimation bias (*peb*) values over all replications for the degrees of freedom parameter ν in Class 2. **(A)** Complete range including all outliers; **(B)** restricted range between −0.6 and 6, illustrating the medium 50% of the distribution. *j* = number of occasions; N = sample size; skew = class-specific skewness parameter for the trait factor (δ_*Tc*_).

Generally, unclear class separation and Mplus warnings occurred less frequently with increasing number of observations and occasions. Additionally, for models with larger skewness (δ_*Tc*_ = 6) more estimation problems were detected. Nevertheless, it should be noted that extreme values for parameter estimates were still present in replications without any warning messages. For an example see the boxplot diagrams of the *peb* values per replication for the degrees of freedom parameter ν_2_ in Class 2 (Figure 4).

### Model performance across all parameters

**Figure 5:**
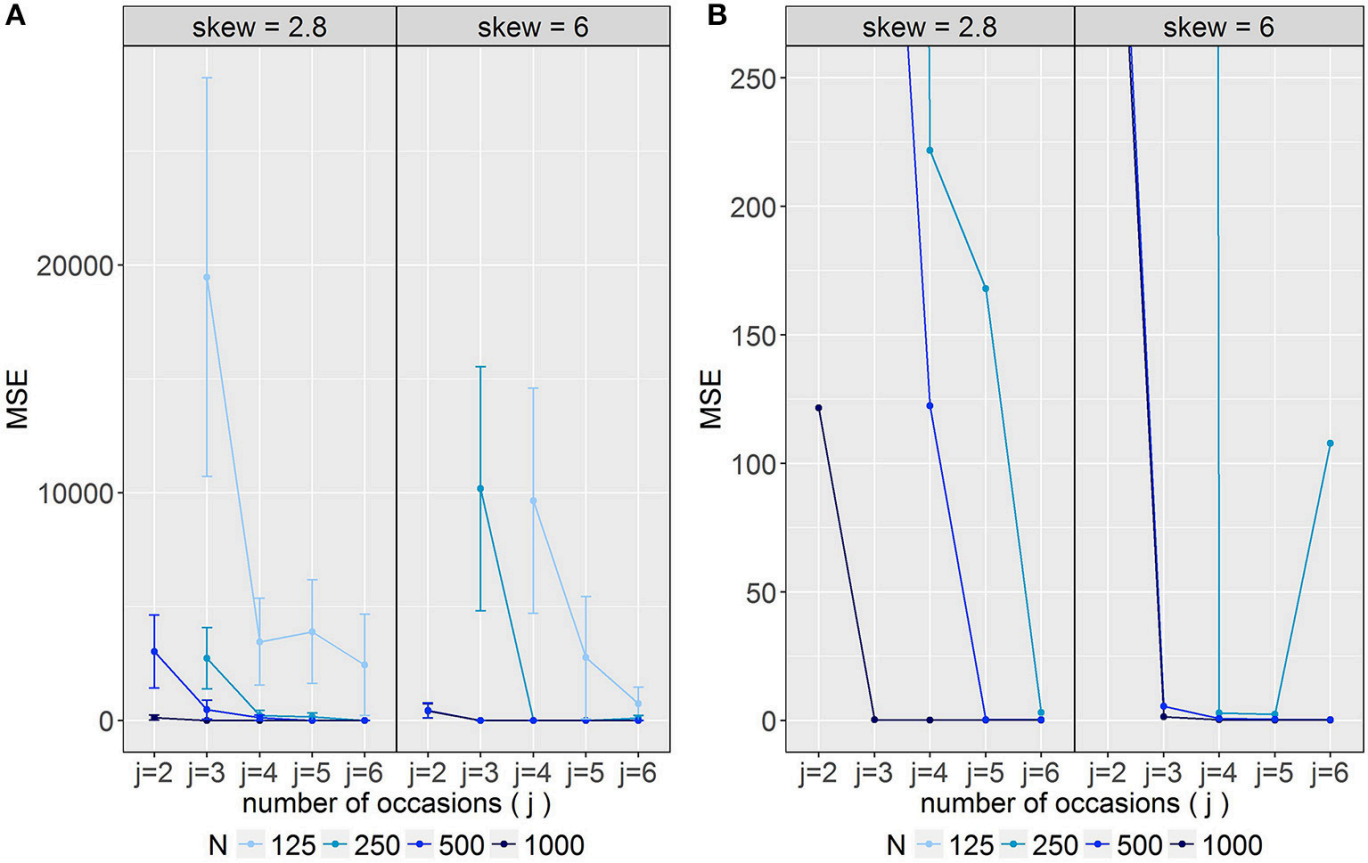
Average mean squared error *(MSE*) across all parameters **(A)** with unlimited y-axis, **(B)** with limited y-axis scaling to visualize models with low average *MSE* values. Error bars represent standard errors. For *j* = 2 and *j* = 3 some conditions are not displayed, because they were excluded from the analysis. *j* = number of occasions; N = sample size; skew = class-specific skewness parameter for the trait factor (δ_*Tc*_).

The mean *MSE* across parameters is displayed in Figure 5. Values steadily decreased with increasing number of occasions and observations. Moreover, different effects of *j* and *N* depending on the skewness were observed: Under conditions with high skewness (δ_*Tc*_ = 6) sample sizes of *N* = 500 yielded rather low *MSE* values even for three occasions, whereas for δ_*Tc*_ = 2.8 five occasions were necessary to reveal comparable values. For models with 250 observations and δ_*Tc*_ = 6 mean *MSE* was low for four and five occasions and increased for *j* = 6. In contrast, for models with 250 observations and δ_*Tc*_ = 2.8, relatively low *MSE* values were observed for six occasions only.

**Figure 6:**
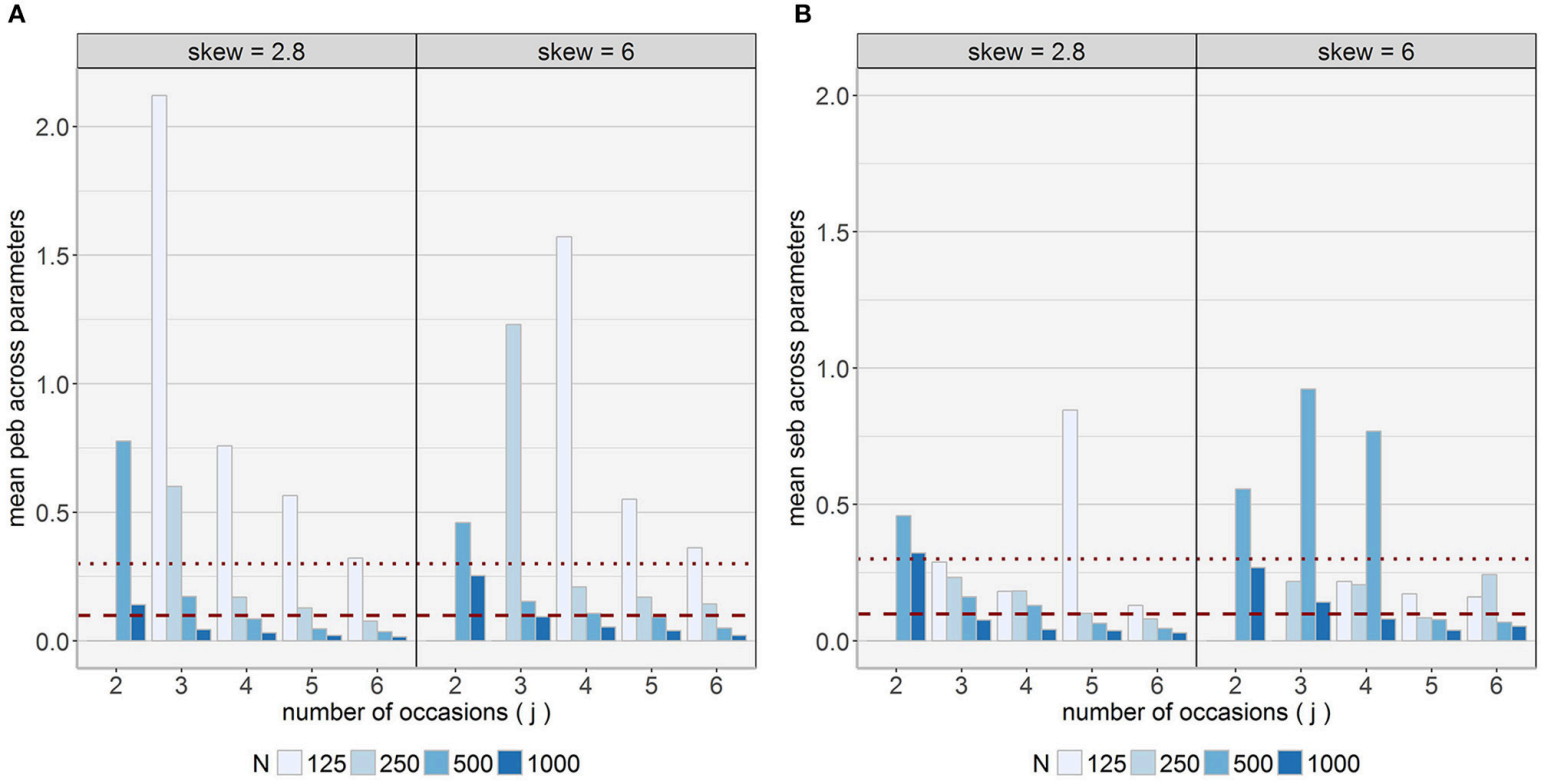
Average absolute bias across parameters per condition. **(A)** Parameter estimation bias (*peb*); **(B)** standard error bias (*seb*). The horizontal dashed lines represent the cut-off for medium bias (10% < *peb/ seb* < 30%), the horizontal dotted lines represent the cut-off for large bias (*peb/ seb* > 30%). *j* = number of occasions; N = sample size; skew = class-specific skewness parameter for the trait factor (δ_*Tc*_).

Average absolute *peb* values across parameters decreased with increasing number of occasions and observations (see Figure 6A). Furthermore, average bias was smaller for mild (δ_*Tc*_ = 2.8) as compared to high skewness (δ_*Tc*_ = 6). With respect to SE estimation accuracy no clear patterns with respect to effects of *j, N* and δ_*Tc*_ could be observed (see Figure 6B).

### Differences between parameters

**Figure 7:**
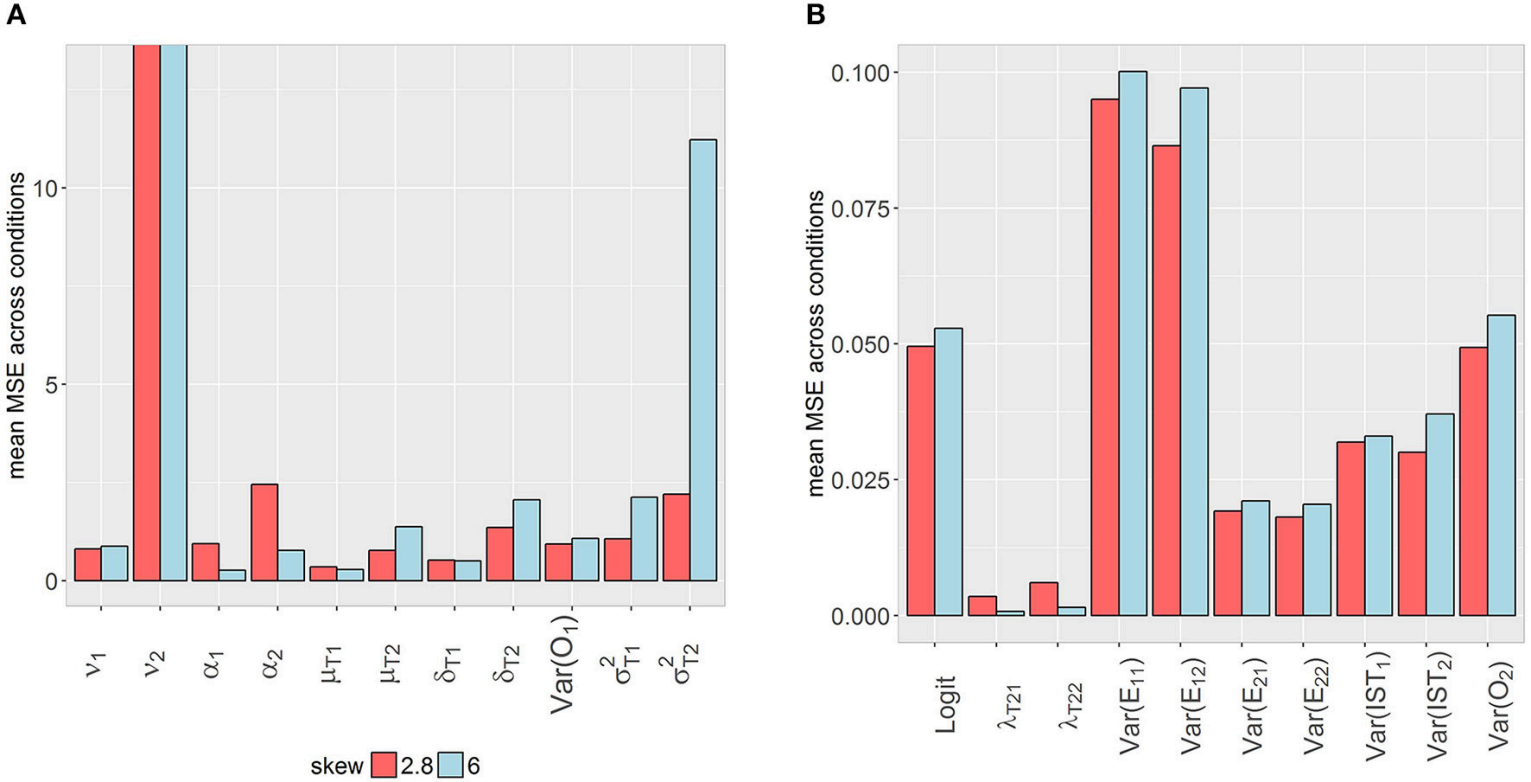
Average mean squared error (*MSE*) across conditions for the different parameters. The range of the y-axes differs between panel **(A)** displaying parameters with comparably high *MSE* values and panel **(B)** displaying parameters with comparably low *MSE* values. To compare the skewness conditions, the model with 3 occasions, 125 observations and δ_*Tc*_ = 2.8 was eliminated for this visualization as a corresponding model with δ_*Tc*_ = 6 was previously excluded from the analysis. The bars for ν_2_ were cut off in order to ensure visibility of the values for the other parameters (mean *MSE* for ν_2_ in conditions with δ_*Tc*_ = 2.8 was 20,694; mean *MSE* for ν_2_ in conditions with δ_*Tc*_ = 6 was 30,281). *c* = index for class; *E*_*jc*_ = residual variable; *j* = index for occasions; *O* = latent occasion-specific variable; *IST*_*c*_ = latent indicator-specific residual trait variable for the second indicator; *T*_*c*_ = latent trait variable; α_2*c*_ = intercept of the second indicator; ν_*c*_ = degrees of freedom parameter; δ_*Tc*_ = skewness parameter for *T*_*c*_; λ_*T*2*c*_ = trait loading of the second indicator; μ_*Tc*_ = location parameter for *T*_*c*_; $\sigma_{Tc}^{2}=$ scale parameter for *T*_*c*_.

The analysis revealed great differences between parameters. Large problems regarding parameter estimation accuracy across all conditions occurred for ν_2_ and $\sigma_{T2}^{2}$ with mean absolute *peb* values of 4.48 and 1.74, respectively (large bias). Furthermore, $\sigma_{T1}^{2}$and δ_*Tc*_ revealed average absolute *peb* values above 0.1 (medium bias) across conditions. These parameters also showed high mean *MSE* values across conditions, as displayed in Figure 7. *MSE* values were lower for parameters in Class 1 compared to Class 2 for ν_*c*_, $\sigma_{Tc}^{2},\delta_{Tc},\alpha_{2c}$, and μ_*Tc*_, whereas for *Var*(*O*_*c*_) lower values for Class 2 were identified. With respect to the other parameters the mean differences in *MSE* values between classes were negligible (< 0.02). Moreover, the effect of δ_*Tc*_ on *MSE* values differed between parameters: Greatest differences to the disadvantages of high skewness were observed for $\sigma_{Tc}^{2},\delta_{T2}$, ν_2_, μ_*T*2_, and *Var*(*O*_1_). In contrast, high skewness produced better estimation for the intercepts. For the other parameters the mean differences between the two skewness conditions were small (difference ≤ 0.100). To shed a more detailed light on the different parameters, results are described separately for (a) the class sizes/logit parameter of belonging to Class 1, (b) parameters referring to the skew *t*-distribution, i.e., δ_*Tc*_ and ν_*c*_, (c) scale parameters/variances of the latent variables, (d) residual variances, and (e) location parameters, intercepts, and loading parameters in the following. A summary table for the results is presented in the Supplementary Material.

#### Class sizes

In most of the models the logit parameter was underestimated indicating that the average proportion of observations belonging to Class 1 was underestimated. Deviations decreased with increasing number of occasions and observations and were slightly smaller for δ_*Tc*_ = 2.8 compared to δ_*Tc*_ = 6. Absolute *peb* values ranged between 0.126 for the condition with δ_*Tc*_ = 2.8, *j* = 3 and *N* = 125 and 0.002 for the condition with δ_*Tc*_ = 2.8, *j* = 5 and *N* = 125. For conditions with at least 500 observations absolute *peb* values lay between 0.003 and 0.025 indicating small deviations from the population value. It should be noted that all replications with π_1_ values between 0.40 and 0.60 were excluded as outlined above so that the following results are based only on replications with clear class separations.

**Figure 8:**
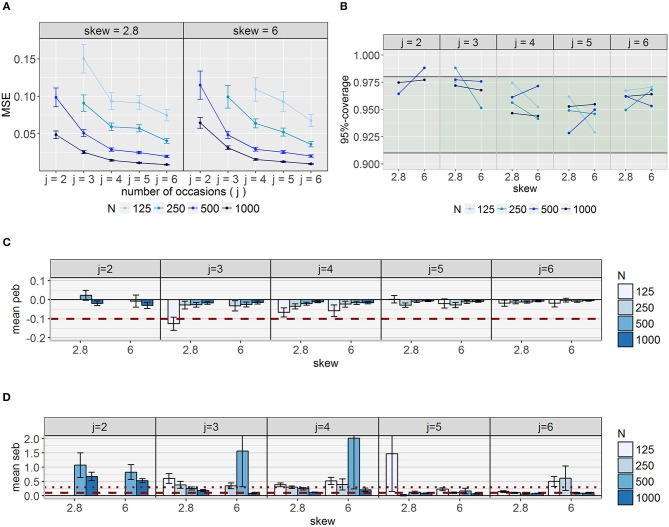
Evaluation criteria for the logit parameter per condition. **(A)** Mean squared error (*MSE*); **(B)** 95% coverage with desired range printed in green; **(C)** average parameter estimation bias (*peb*) across replications; **(D)** average standard error bias (*seb*) across replications, to visualize small values, the y-axis was cut off; the horizontal dashed lines represent the cut-offs for medium bias (10% < absolute *peb/ seb* < 30%), dotted lines represent the cut-offs for large bias (absolute *peb/ seb* > 30%). Error bars represent standard errors. The scaling of the y-axis differs between panels. *j* = number of occasions; N = sample size; skew = class-specific skewness parameter for the trait factor (δ_*Tc*_).

The evaluation criteria for the logit parameter are presented in Figure 8. *MSE* (Figure 8A) decreased steadily with increasing number of occasions and observations with similar patterns for both skewness conditions. Coverage values (Figure 8B) lay in the desired range for almost all conditions with rates higher than 98% only for two conditions (*j* = 2, *N* = 500, δ_*Tc*_ = 6 and *j* = 3, *N* = 250, δ_*TC*_ = 2.8). With respect to parameter estimation accuracy (Figure 8C), *peb* fell below the cut-off for all conditions except for *j* = 3, *N* = 125, δ_*Tc*_ = 2.8. Regarding SE estimation accuracy (Figure 8D) at least medium bias was identified in most conditions. *Seb* values were acceptable for at least 5 occasions and 500 observations, but regarding the differential effect of *j, N*, andδ_*Tc*_, there was no clear pattern.

#### Parameters related to the skew *t*-distribution

**Figure 9:**
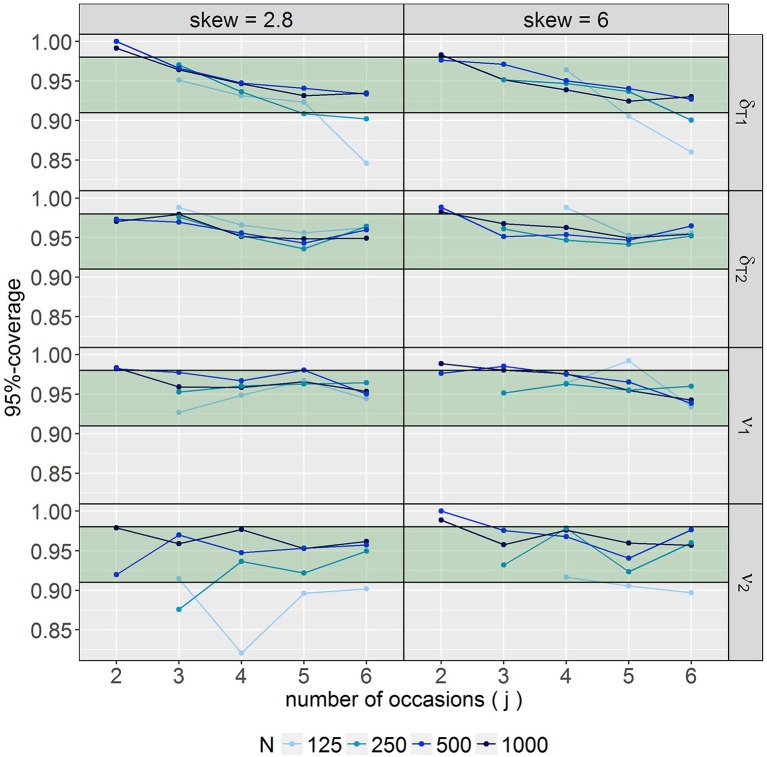
95% coverage for the parameters referring to the skew *t*-distribution. The desired range of 0.91–0.98 is printed in green. ν_*c*_ = degrees of freedom parameter for class *c*; δ_*Tc*_ = skewness parameter for *T*_*c*_ for class *c*.

Coverage of ν_*c*_ and δ_*Tc*_, as displayed in Figure 9, showed too high coverage for conditions with *j* = 2 (>0.98 for almost all four parameters). Especially forν_2_, values below the desired interval were observed for conditions with mild skewness and *N* = 125. Furthermore, in some conditions with *N* = 125 and 250 in combination with large *j*, coverage of δ_*T*1_ was below 0.91. Considering all four parameters in common, coverage fell in the desired interval for models with at least 500 observations and 4 occasions in case of high skewness and for models with at least 250 observations and 4 occasions in case of mild skewness. With respect to the other conditions no clear patterns regarding the over- or underestimation of coverage were identified.

**Figure 10:**
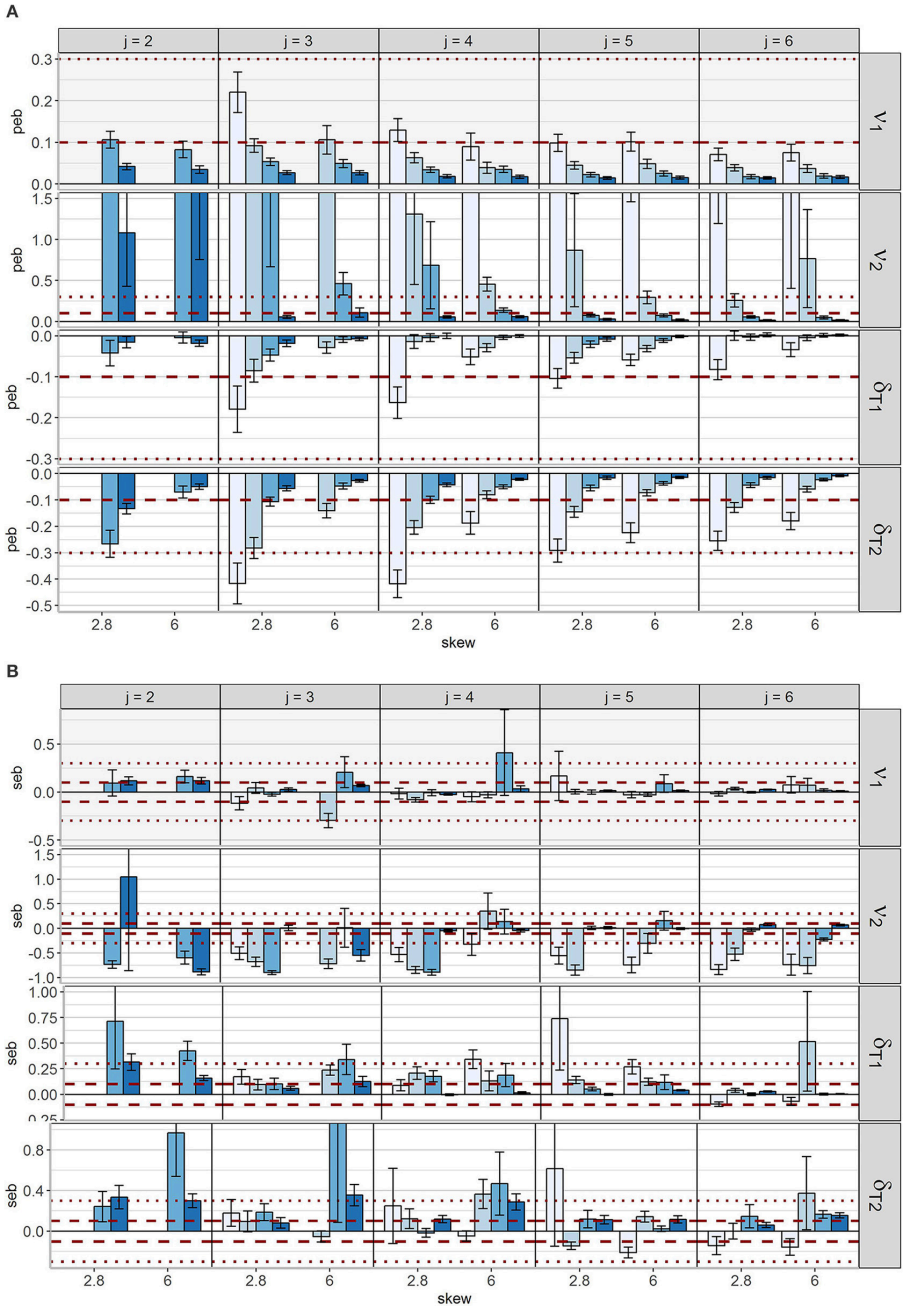
Mean bias across conditions for parameters referring to the skew *t*-distribution. The horizontal dashed lines represent the cut-offs for medium bias (10% < absolute *peb* < 30%), the horizontal dotted lines represent the cut-offs for large bias (absolute *peb* > 30%). To visualize small values, the y-axis of ν_2_ was cut off. The scaling of the y-axis differs between parameters. Error bars reflect standard errors. ν_*c*_ = degrees of freedom parameter for class *c*; δ_*Tc*_ = skewness parameter for *T*_*c*_ for class *c;* **(A)** parameter estimation bias (*peb*); **(B)** standard error bias (*seb*).

As depicted in Figure 10A, parameter estimation accuracy generally increased with increasing number of observations and occasions for all four parameters. For δ_*Tc*_ and ν_2_ most conditions with high skewness were less sensitive to bias compared to the corresponding conditions with mild skewness. Nevertheless, more conditions with δ_*Tc*_ = 2.8 revealed unbiased estimates of all four parameters in common. Whereas for *N* = 1,000 three occasions were sufficient in case of δ_*Tc*_ = 2.8, at least four occasions were necessary for δ_*Tc*_ = 6 and the same sample size. For *j* = 5 all conditions with *N* ≥ 500 revealed unbiased degrees of freedom and skewness parameters.

*Peb* values were highest for ν_2_, which was overestimated in many conditions. Mean *peb* values between 11.96 and 39.89 occurred for ν_2_ under conditions with low sample sizes and occasions. The cut-offs for medium (0.10 < *peb* < 0.30) and large bias (*peb* > 0.30) were exceeded in 4 and 20 conditions, respectively. Inspecting the boxplot diagrams of *peb* values per replication (see Figure 4) revealed many outliers. For some models (e.g., δ_*Tc*_ = 2.8, *j* = 3, *N* = 125), even the central 50% of the distribution included values of 2.7 indicating 270% bias.

The other parameters related to the skew *t*-distribution were less sensitive to bias: For ν_1_, unbiased estimates were observed for models with at least 3 occasions and 250 observations, except for the condition with 3 occasions and 250 observations in combination with δ_*Tc*_ = 6 (*peb* = 11%). In the case of bias, the population values of the skewness parameters were underestimated. Downward bias for δ_*T*1_ occurred only in few conditions with 125 observations (maximum bias of 16%). *Peb* of δ_*T*2_ depended on the degree of skewness: When skewness was high, at least 250 observations and four occasions or fewer occasions in combination with at least *N* = 500 were necessary for acceptable values. For mild skewness a minimum of *N* = 500 and *j* = 5 was required for *peb* values below the threshold.

SE estimation accuracy is displayed in Figure 10B. Patterns with respect to influences of *j, N* and δ_*Tc*_ on *seb* were not clear. In general, solely for one model with *j* = 6, *N* = 1,000 and δ_*Tc*_ = 2.8, acceptable *seb* values could be detected for all parameters referring to the skew *t*-distribution.

#### Scale parameters/variances of the latent variables

Coverage of *Var*(*IST*_21_), *Var*(*O*_1_) and $\sigma_{T2}^{2}$ was below .91 for only few conditions with *N* = 125 or *N* = 500 in combination with *j* = 2. For conditions with *N* ≥ 500 in combination with high skew conditions with *j* = 2 and *j* = 4 coverage values of *Var*(*IST*_21_) lie above 0.98. For $\sigma_{T1}^{2}$ and the other latent variances, coverage values fell in the desired interval.

**Figure 11:**
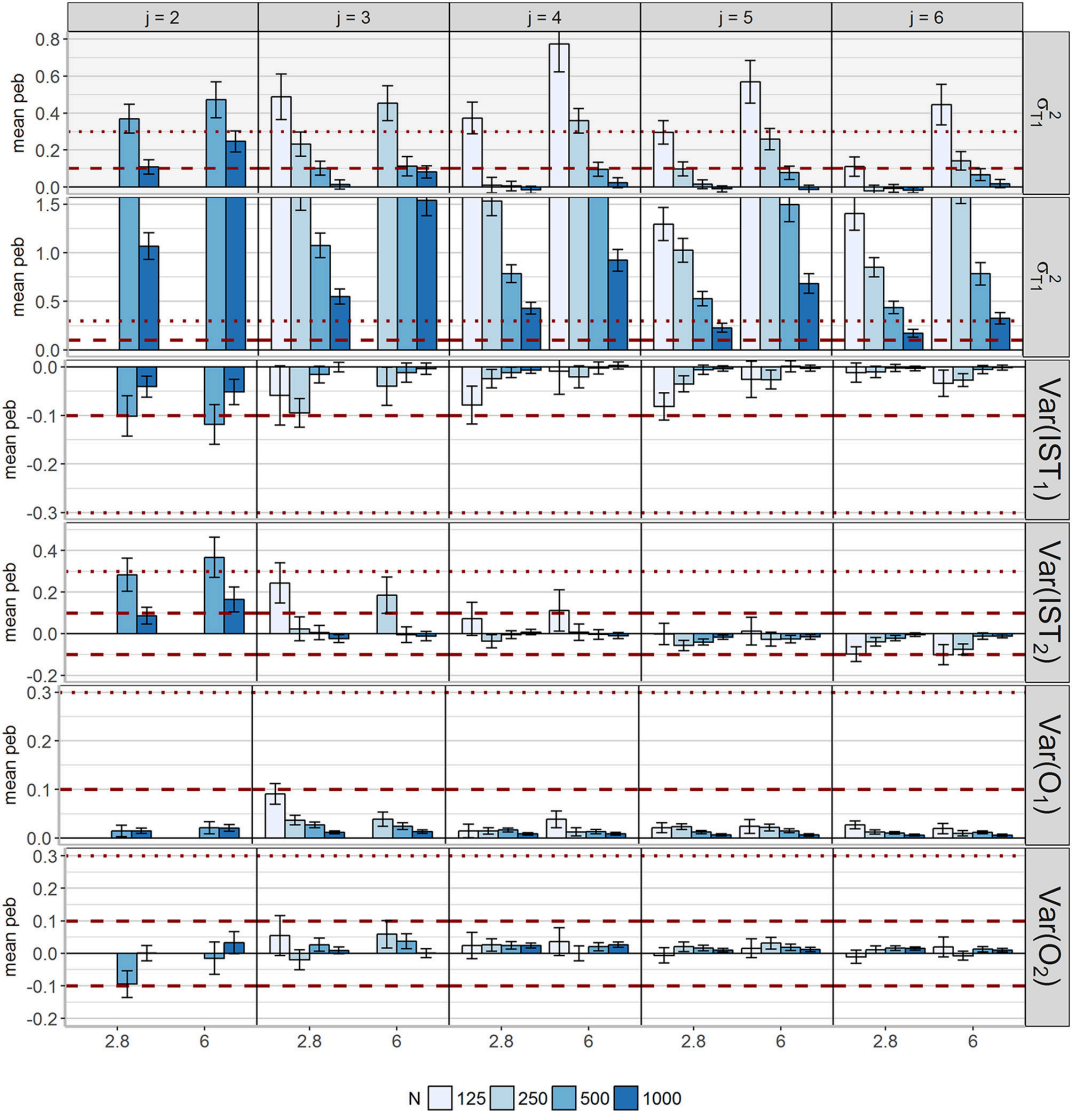
Mean parameter estimation bias (*peb*) across replications for the variances/ scale parameters of the latent variables. Error bars reflect standard errors. The red dashed lines represent the cut-off for medium bias (10% < absolute *peb* < 30%), dotted lines represent the cut-off for large bias (absolute *peb* > 30%). To visualize small values, the y-axis of $\sigma_{T2}^{2}$ was cut off. The scaling of the y-axis differs between parameters; *c* = index for class; *IST*_*c*_ = indicator-specific trait residual factor; *j* = occasions; *O*_*c*_ = occasion-specific factor; skew = class-specific skewness parameter for the trait factor (δ_*Tc*_); $\sigma_{Tc}^{2}=$ scale parameter for *T*_*c*_.

Parameter estimation accuracy differed between parameters as depicted in Figure 11: Whereas *Var*(*O*_*c*_) were not sensitive to bias and *Var*(*IST*_21_) exhibited medium bias (10–12%) only in conditions with *j* = 2 and *N* = 500, *peb* values of *Var*(*IST*_22_) and $\sigma_{Tc}^{2}$ more often exceeded the cut-offs. For *Var*(*IS*_*T*_22_)_, population values were overestimated in conditions with few occasions and underestimated in conditions with more occasions. Some models with *j* = 2 and *j* = 3 exhibited bias above 10% (16–37% bias) with generally larger values for high skewness conditions. Values were acceptable for most conditions with four or five occasions, but increasing the number of occasions to *j* = 6 increased bias in direction of the threshold of −0.1. *Peb* was even higher for the variances of the traits. Bias decreased with increasing *N* and *j* as well as decreasing δ_*Tc*_. Whereas for $\sigma_{T1}^{2}$ acceptable values for large *N* and/or large *j* occurred, there was no condition for which the bias of $\sigma_{T2}^{2}$ fell below the cut-off. Relative bias ranged between 17% and 609% for this parameter.

**Figure 12:**
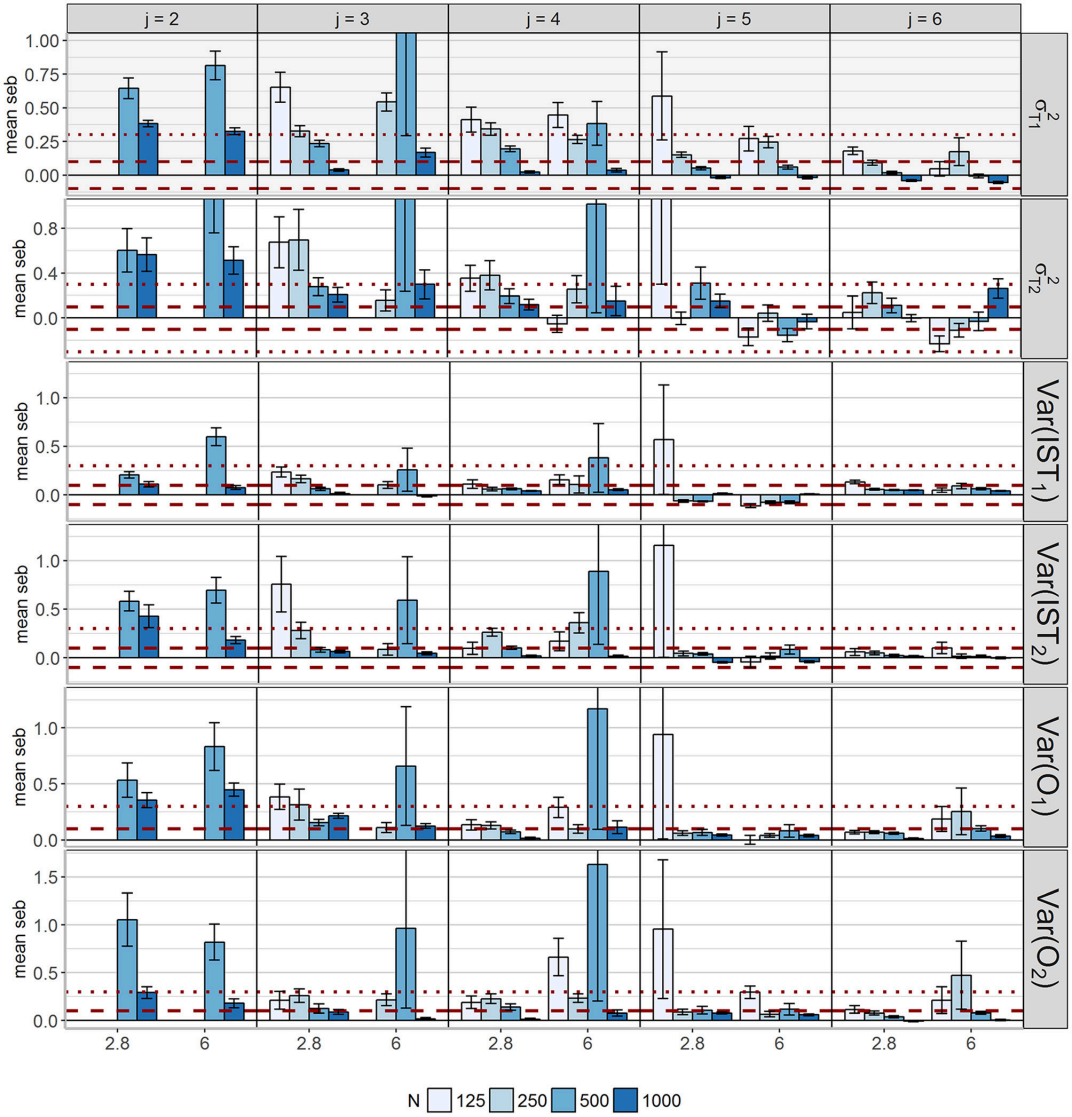
Mean standard error bias (*seb*) across replications for the variances of the latent variables. Error bars reflect standard errors. The red dashed lines represent the cut-off for medium bias (10% < absolute *peb* < 30%), dotted lines represent the cut-off for large bias (absolute *peb* > 30%). To visualize small values, the y-axes of $\sigma_{Tc}^{2}$ were cut off. The scaling of the y-axis differs between parameters; *c* = index for class; *IST*_*c*_ = indicator-specific trait residual factor; *j* = occasions; *O*_*c*_ = occasion-specific factor; skew = class-specific skewness parameter for the trait factor (δ_*Tc*_); $\sigma_{Tc}^{2}=$ scale parameter for *T*_*c*_.

Again *seb* showed diffuse patterns for all parameters (Figure 12). Acceptable *seb* values with respect to all latent variances were identified for five occasions and 1,000 observations.

#### Residual variances

Generally, *peb* values exceeded the cut-offs solely in Class 2 for few conditions: *Var*(*E*_12_) exhibited 10% (δ_*Tc*_ = 6) and 11% (δ_*Tc*_ = 2.8) bias for models with *j* = 4 and *N* = 125, and *Var*(*E*_22_) showed 11% (δ_*Tc*_ = 6) and 12% (δ_*Tc*_ = 2.8) bias for conditions with *j* = 2 and *N* = 500. SE estimation accuracy was lower than parameter estimation accuracy without clear effects of *j, N* and δ_*Tc*_. Severe bias occurred even in conditions with large *j* and *N*, and only conditions with *j* ≥ 4 and *N* = 1,000 showed acceptable *seb* values across all four residual variance parameters.

#### Intercepts, trait loadings, and location parameters of the latent trait variables

Coverage showed the most problems for λ_*T*22_ with values > 0.98 under most conditions with high skewness and 125 or 250 observations. Increasing the sample size to 1,000 revealed values near 0.95 for at least four occasions. With respect to μ_*Tc*_ and α_2*c*_ coverage did not reach 0.91 under some conditions with *N* = 125. Furthermore, for *j* = 2 and *N* = 500 in combination with mild skew, coverage values for μ_*T*1_ lay above the desired interval and for μ_*T*2_ below the desired interval.

With respect to parameter estimation, λ_*T*2*c*_ and μ_*Tc*_ were not sensitive to bias. The intercepts were biased solely in few conditions. α_21_ was only afflicted by bias in conditions with δ_*Tc*_ = 2.8 and acceptable values occurred for all conditions with at least 500 observations and 4 occasions. Absolute bias values for Class 2 were higher with also some conditions with high skewness being biased. For *N* ≥ 500 in combination with *j* ≥ 3, parameter estimation appeared to be unbiased for both degrees of skewness.

Again average *seb* for intercepts, trait loadings and location parameters of the trait variable exceeded the cut-offs for many conditions without clear patterns regarding the effects of *j, N*, and δ_*Tc*_. Mostly SEs were biased upwards. Patterns for intercepts and trait loadings were very similar and generally large *j* in combination with large *N* revealed acceptable values. Patterns for μ_*Tc*_ were similar to the patterns for $\sigma_{Tc}^{2}$.

#### Trends of common bias between parameters

**Figure 13:**
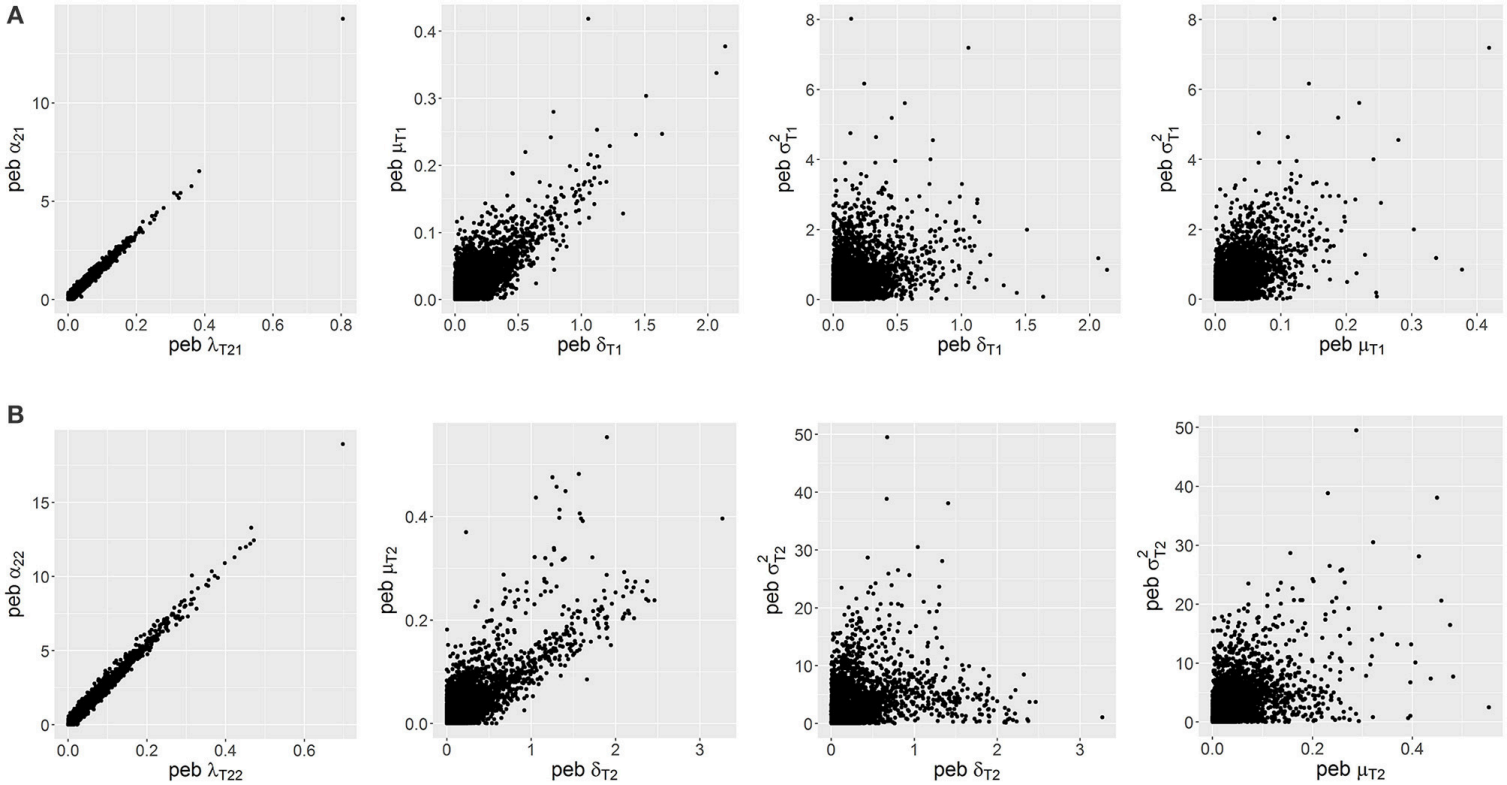
Relationships between absolute parameter estimation bias (*peb*) values of the different parameters across replications for **(A)** Class 1 and **(B)** Class 2. The scaling of the axes differs between plots; α_2*c*_ = intercept of the second indicator; *c* = index for class, δ_*Tc*_ = skewness parameter for *T*_*c*;_ λ_*T*2*c*_ = trait loading of the second indicator; μ_*Tc*_ = location parameter for *T*_*c*_; $\sigma_{Tc}^{2}=$ scale parameter for *T*_*c*_.

By inspecting correlations and bivariate distributions of absolute *peb* values of the different parameters, some patterns could be observed as depicted in Figure 13. Within classes, trait loadings and intercepts were nearly perfectly correlated (*r*>0.96). Furthermore, δ_*Tc*_, μ_*Tc*_, and $\sigma_{Tc}^{2}\;$showed similar trends for the first class (0.28 < *r* < 0.56) as well as for the second class (0.37 < *r* < 0.47). For absolute *seb* values similar patterns were identified. Bias often occurs for all three parameters related to the (skewed) class-specific trait value in common. Across classes bias did not show common trends. Tables with bivariate Spearman's ρ correlations can be found in the Supplementary Material.

### Model performance considering different parameter types

**Figure 14:**
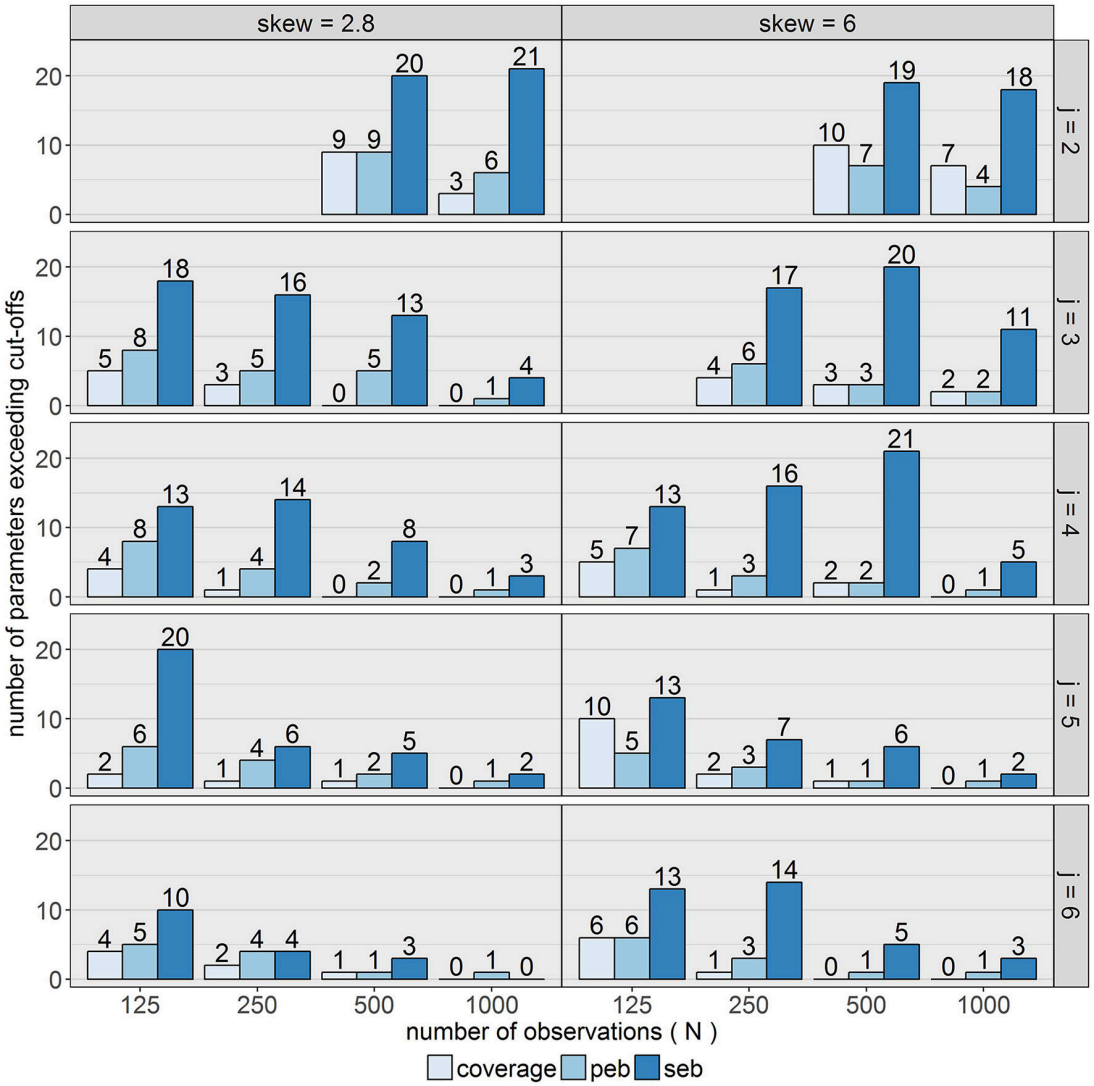
Number of parameters exceeding the cut-off values of the different criteria. The total number of estimated parameters in the design was 21. Coverage was considered acceptable between 0.91 and 0.98. Cut-off (medium bias) for unacceptable *peb* and *seb* values was set to 0.1. *j* = number of occasions; *peb* = parameter estimation bias; *seb* = standard error bias; skew = class-specific skewness parameter for the trait factor (δ_*Tc*_).

In order to evaluate model performance considering the estimation accuracy of different parameter types, it was inspected how many parameters per model exceeded the cut-offs for *peb, seb* and coverage. As displayed in Figure 14, none of the models was free from bias. At least one parameter, $\sigma_{T2}^{2}$, was biased in each of the conditions. For *N* ≥ 500 in combination with *j* ≥ 5 and *N* = 1,000 in combination with *j* = 4 (high skewness) or *N* = 1,000 in combination with *j* ≥ 3 (mild skewness) *peb* of all other parameters lay in the desired range.

The total number of exceeded cut-offs for coverage, *peb* and *seb* mostly decreased with increasing *j, N* and lower skew. For *j* = 3 and *j* = 4 in combination with high skewness, some diffuse patterns were identified. Differences between skewness conditions declined for conditions with larger numbers of occasions and observations. In general, most problems occurred with respect to SE estimation accuracy (*seb*), but in contrast to parameter estimation accuracy, one condition (*j* = 6, *N* = 1,000, δ_*Tc*_ = 2.8) revealed acceptable *seb* values for all parameters. In conclusion, the skew *t* mixture LST model exhibited many problems with respect to SE estimation and failed to recover the true population values for at least one parameter in all conditions. Tables containing information about parameter and SE estimation per parameter for each condition are provided in the Supplementary Material.

## Discussion

The purpose of this MC simulation study was to evaluate the statistical performance of a skew *t* mixture LST model, a combination of the mixture LST model introduced by Courvoisier et al. (2007) and the skew *t* approach proposed by Asparouhov and Muthén (2015). The mixture LST model allows for individual as well as structural differences in intraindividual variability. It aims to identify distinct numbers of interpretable latent classes with class-specific model parameters. According to the skew *t* component of the model, non-normal distributions within classes are allowed. This reduces the risk of identifying spurious latent classes due to non-normality of the outcomes, as this more flexible distribution accounts for asymmetry or heavy tails (Lin et al., 2015).

In LST research non-normal variables are observed frequently (Eid et al., 1999) and theories about population heterogeneity with respect to the stability and variability of constructs are present (Baumeister and Tice, 1988). Hence, LST models offer a promising framework for both applications of mixture SEMs and the skew *t* approach. As these aspects have not been combined so far, the skew *t* mixture LST model fills a gap in the current literature.

Previous simulation studies with (LST) mixture models and skew *t* factor mixture models identified effects of sample size, number of occasions and degree of skewness on model performance (Courvoisier et al., 2007; Tueller and Lubke, 2010; Lin et al., 2015). These aspects were varied in this simulation study in order to find out under which conditions parameters of the skew *t* mixture LST model can be appropriately estimated.

In the following, the results regarding convergence, parameter and SE estimation are discussed and recommendations for applications in practice are given.

### Convergence

Even though convergence rates for the skew *t* mixture LST model were high and for small *N* and *j* even higher as compared to the normal mixture LST model (Courvoisier et al., 2007), many replications revealed improper solutions as indicated by warnings given in Mplus. Conditions with few observations and occasions exhibited more issues, especially in combination with high skewness. This is in line with Kooken (2015) applying a normal GMM with three measurement occasions for skewed data where the skewed approach produced an improper solution although the sample sizes were relatively large (*N* ≥ 1,822 depending on the occasion).

Lubke and Muthén (2007) state that class-specific covariance matrices of the observed variables, determined by class-specific parameters, may lead to singularities and convergence problems. However, identification of the parameters that vary across classes is a key goal in the context of mixture LST models. Lubke and Muthén used fifty sets of random starting values in order to reduce convergence problems. In our study, the true population values were chosen as starting values for optimal starting conditions for the estimation process. Additionally providing multiple starting values may have decreased the rate of improper solutions. However, by using population values as starting values, the approach was investigated under ideal conditions, and bias results are assumed to represent a lower limit of bias that can be expected with less ideal starting values.

For the replications revealing warning messages in the current simulation study, often variances of the latent variables were fixed to zero in order to avoid the singularity of the information matrix. This may be due to the relatively small population values of the latent variances. From a theoretical viewpoint, zero variances may be reasonable in research fields such as psychiatry: If a subgroup not showing a specific symptom or behavior exists, this leads to a zero variance for a latent trait variable. For example, for the addiction severity index, a semi structured interview assessing seven areas of functioning for patients with substance use disorders, in practice often many zero values (no severity) occur (Delucchi and Bostrom, 2004).

### Parameter estimation

*MSE* values steadily decreased with increasing *N* and *j*, although none of the models was free from parameter estimation bias (*peb*): Generally, cut-offs for bias were less frequently exceeded for models with larger *N* and *j*. Nevertheless, at least one parameter showed unacceptable *peb* values in each model.

Across all conditions, parameters related to the trait variables *T*_*c*_ were most sensitive to bias. In contrast, for the normal mixture LST model bias was larger for the intercepts, trait loadings and variances of the indicator-specific traits as compared to the other parameters (Courvoisier et al., 2007). This underlines that model performance greatly varies depending on the assumption of the functional form of the distribution within classes. Especially when skew *t* models are compared to (skew) normal models, biased estimates for δ_*Tc*_ and ν_*c*_ are problematic: Values for δ_*Tc*_ near zero and/or large values of ν_*c*_ may suggest another functional form within classes and, therefore, lead to misinterpretations.

Bias of the logit parameter was generally low for the skew *t* mixture LST model, exceeding the cut-off solely in one condition. Parameter estimation accuracy appeared to be acceptable even for models with small numbers of occasions and observations. Thus, class proportions were recovered well and problems were mainly related to parameters within classes. However, it should be noted that all replications with unclear class separation were excluded because it remained unclear whether label switching occurred or not (Tueller et al., 2011).

Bias for ν_*c*_, $\sigma_{Tc}^{2},\;\delta_{Tc},\;\alpha_{2c}$, and μ_*Tc*_ was more pronounced in Class 2 which may be due to the smaller class size (π_2_ = 24%) resulting in less information available to estimate the parameters. For instance, for conditions with *N* = 125, solely 30 observations were expected in this class. Especially with respect to the parameters belonging to the skewed trait factor, few observations appear not to be sufficient for unbiased estimates. As depicted in Figure 2, a degrees of freedom parameter of ν_*c*_ = 5 leads to heavy tails in the expected distribution. Within a small sample there may be only very few outlier values so that the skewed variables cannot be appropriately recovered. This seems to be even more problematic for larger skewness, as higher *MSE* values of$\;\sigma_{Tc}^{2},\;\delta_{T2}$, ν_2_, and μ_*T*2_ for conditions with high skewness indicate.

For some conditions with many observations (*N* ≥ 500) and occasions (*j* ≥ 5) solely $\sigma_{Tc}^{2}\;$ exhibited upward bias. In these models, in contrast to *peb* values, 95% coverage of the parameter was acceptable and *MSE* values were comparatively small. These results indicate that increasing the number of occasions and observations may lead to unbiased parameter estimates. However, based on our results predictions regarding the required sample size and number of measurement occasions for unbiased estimates cannot be made. In practice *j* > 6 and *N* > 1,000 are rarely realized. According to formula (19) (p. 4) given in Asparouhov and Muthén (2015), a larger scale parameter σ^2^ of a variable following a skew *t*-distribution leads to a larger variance of this variable. From a theoretical viewpoint upward bias of the trait variance indicates a greater degree of interindividual differences of the general dispositions. As the variance of the traits is used for the calculation of important coefficients indicating the degree of stability and variability within the LST framework (Steyer et al., 1992; Eid and Luhmann, 2012), biased estimates of $\sigma_{Tc}^{2}$ resulting in biased trait variances, are crucial in applications. As MC simulation studies evaluate models under ideal conditions (Muthén and Muthén, 2002), even more bias can be expected in applications.

### SE estimation

More problems as compared to parameter estimation occurred with respect to SE estimation. Solely one condition was free from bias, and no clear results regarding differential effects of *N, j* and δ_*Tc*_ could be observed. As coverage is influenced by SE estimation (Muthén and Muthén, 2002), diffuse patterns regarding coverage are in line with the diffuse patterns of *seb* across conditions.

For the calculation of *seb* values, the SDs of parameter estimates serve as population values for SEs (Bandalos, 2013). SDs were large for some parameters (especially ν_2_) in conditions with few observations and occasions even though replications with warning messages were excluded. The remaining replications also contained outliers enlarging the SDs. As the calculations of parameters related to the skew *t*-distribution influence each other, outlier values of one parameter may cause outlier values for another parameter as well. It remains unclear whether the outliers represent parts of the distribution or problematic values that should not be considered. Therefore, the SDs influenced by these large values may not be trustworthy and cause diffuse results regarding SE estimation. Furthermore, the diffuse *seb* patterns may be due to the different numbers of included replications.

For the normal mixture LST model, diffuse *seb* patterns were observed as well, but for large *N* and *j* bias fell below the cut-off for all parameters (Courvoisier et al., 2007). In contrast to Courvoisier et al. (2007), some parameters of the skew *t* mixture LST model, i.e., location and scale parameters of the trait variables or intercepts and loadings, exhibited similar *seb* patterns within classes across conditions. This is in line with common trends of absolute *peb* values for the respective parameters and underlines that parameter estimation in skewed models is more complex and entangled.

*Peb* and *seb* largely varied across replications for the skewness parameters and the latent variances. In order to reduce the effect of outliers and extreme values on bias calculation, we additionally used the median as a measure of central tendency across replications. The results are presented in the Supplementary Material. Compared to the usual definition of bias using the average across replications (see Equations 8 and 9), less bias was observed for small conditions. However, as extreme values can occur in simulation studies, eliminating the values reduces the validity of the results. Therefore, we decided to base our interpretation on the average bias values presented above.

### Limitations and future research

The current work focused on realistic conditions in the LST research domain and was, therefore, closely designed to the only available application of mixture LST modeling. Thus, it remains unclear whether the results are generalizable to other situations. Statistical performance of mixture models depends on further aspects, such as degree of class separation, effect size, proportion of class sizes or parameter invariance between classes (Lubke and Muthén, 2007; Tueller and Lubke, 2010), and these factors may even interact with the skewness of the data. Thus, future simulation studies could investigate (skew *t*) mixture LST models with different population parameters to consider these aspects.

Furthermore, the number of indicator variables for each measurement occasion is known to affect the convergence and parameter estimation (Marsh et al., 1998; Kenny and McCoach, 2003). This may be an additional variable that could be considered in a further simulation study. Moreover, statistical performance may depend on the number of latent subpopulations which may be investigated in further simulation studies as well. In the current design, apart from the residual variances at the first occasion of measurement, all parameters were held equal across time. Due to this MI setting, increasing the number of occasions is similar to increasing the sample size. From a theoretical viewpoint in LST research, the numbers of observations and occasions are two distinct aspects in applications. Therefore, to examine differential influences of these factors on statistical performance of models in more detail, future simulation studies could include different degrees of MI across time. However, it should be noted that less restrictive models need more parameters to be estimated which may complicate model estimation (Lubke and Muthén, 2007). This study solely examined the statistical performance in terms of parameter and SE estimation. Future work could focus on the ability of the skew *t* approach to detect the correct number of classes. Nevertheless, as starting values can greatly influence estimation in mixture models, it should be carefully considered which values to provide for three class solutions. Additionally, class assignment accuracy and its relationship to parameter and SE estimation could be studied. Some applications of mixture SEMs focus on assigning individuals to their most likely class, but even in case of accurate parameter estimation, classification quality can be poor, so that individuals may be incorrectly classified (Lubke and Tueller, 2010). Therefore, identifying conditions under which class assignment accuracy is high may be of particular interest for some research domains and applications such as categorical diagnostic in psychiatry.

Future research could compare normal and skew *t* mixture SEM and their respective identified number of classes, as done for example by Muthén and Asparouhov (2015) in the context of GMMs. In various contexts (e.g., biological, economic, and psychological research), where non-normal distributions are plausible, these comparisons may offer different perspectives and therefore, improve the interpretability of the results.

In the current work, the number of included replications greatly differed between conditions due to the exclusion of improper solutions, so that the comparability between conditions is limited. There are debates about how to handle estimation issues (Boomsma, 1985; Chen et al., 2001), and some authors include replications with warning messages in order to consider the entire distribution of the parameters (Leite, 2007; Ulitzsch et al., 2017). However, other authors not only delete replications with improper solutions, but also unrealistic parameter estimates provided by replications without any warning messages. For example, Enders and Bandalos (2001) excluded replications with absolute *peb* values greater than 4, but also state that this cut-off was “clearly arbitrary” (p. 440), based on the rationale that these values were considered problematic in applications. For the current simulation, solely improper solutions were excluded in line with other simulations (Nylund et al., 2007; Geiser and Lockhart, 2012). Warning messages are generally considered to be problematic in empirical applications, but heuristics for additional “problematic” outlier values could not be defined, as modeling with skew *t*-distribution has not been frequently used in applications yet. Future studies could evaluate effects of different inclusion strategies of replications.

## Conclusion

In practice, when researchers are interested in population heterogeneity with respect to reversible short-term fluctuations around a stable trait, mixture LST models are a powerful data-analytical tool. However, researchers should be aware of the classical assumption of within-class normality in normal mixture models. The results should be treated with caution, as in case of non-normality, classes may be simply formed to cover heavy tails of the distributions. That is, if researchers encounter small, non-substantial and hard-to-interpret subgroups or have substantial reasons to assume an underlying skewed distribution, the skew *t* approach might be a valuable alternative. Applying the flexible skew *t* approach may offer different perspectives on the underlying relationships.

The following recommendations for applications can be made according to the results of this simulation study:

Unbiased parameter estimation was not possible for any of the conditions considered in this design. Applying a skew *t* mixture LST model with two classes and trait factor skewness of δ_*Tc*_ = 2.8 and δ_*Tc*_ = 6 in combination with five degrees of freedom is not recommended for small sample sizes (*N* = 125, *N* = 250) and few occasions (*j* = 2). However, the number of affected parameters was relatively small for large sample sizes and number of occasions, and in contrast to bias values, coverage values lay in the desired range. In case of mildly skewed variables, 500 observations in combination with at least four occasions and 1,000 observations with at least three occasions may be sufficient. For variables with high skewness, at least five occasions should be investigated for 500 observations and at least four occasions may be sufficient in case of 1,000 observations. However, as bias still occurs in these large conditions, the results should be treated with caution.

The current simulation underlines that larger sample sizes are necessary for skew *t* mixture SEMs as compared to normal mixture SEMs (Muthén and Asparouhov, 2015): For the normal mixture LST model with two classes at least four occasions and 250 observations were sufficient for unbiased estimates (Courvoisier et al., 2007), but under these conditions parameter estimation was not appropriate for the skew *t* model. Asparouhov and Muthén (2015) reported good results for *N* = 2,000 for skew *t* GMMs or *N* = 5,000 for skew-normal factor analysis, but whether these numbers lead to unbiased results within the skew *t* mixture LST framework has to be tested.

However, Muthén and Asparouhov (2015) argue that successful analyses with the skew *t* approach may be possible for sample sizes between 100 and 200. This may be true for other SEMs with different structural relationships between parameters. There is still more research necessary to investigate the approach in other contexts. For instance, it may be advantageous to assume skew *t*-distributions in other SEM models for longitudinal data. Further simulation studies are needed in this context to evaluate the statistical performance of these models.

In applications, multiple starting values should be used to increase convergence, even more as compared to normal mixture SEMs (e.g., 400 initial stage starts as used by Muthén and Asparouhov, 2015). Furthermore, researchers should be aware of the problem that skew *t* models may exhibit estimation issues even though models converged, especially in case of small sample sizes, few measurement occasions and strongly non-normal data. Model parameters should be carefully checked. Models with warning messages may include meaningful information as well. For instance, fixed values, such as zero variances, may characterize people with response tendencies or, subgroups of specific patients in psychiatry research. Compared to models with proper solutions, those with improper solutions show larger bias across parameters (Chen et al., 2001). Therefore, the models with estimation issues should not be interpreted. Instead, additional models with parameter constraints should be tested. Furthermore, it is clearly recommended to conduct a simulation study with the properties of the model in the respective application, in order to ensure that the fit coefficients and parameter estimates can be trusted.

## Author contributions

LH, JH, and ME contributed conception and design of the study; LH organized the data simulation under supervision of JH; LH performed the statistical analysis; LH wrote the first draft of the manuscript; JH and ME wrote sections of the manuscript. All authors contributed to manuscript revision, read and approved the submitted version.

### Conflict of interest statement

The authors declare that the research was conducted in the absence of any commercial or financial relationships that could be construed as a potential conflict of interest.

## Footnotes

^1^Warning given in Mplus: Due to a low estimate of the degrees of freedom parameter in class 1/class 2 the estimated skewness in that class is infinity. The distributional assumptions of the skew *t*-distribution may not be appropriate.

## References

[B1] Arellano-Valle R. B. Azzalini A. (2013). The centred parameterization and related quantities of the skew-t distribution. J. Multivar. Anal. 113, 73–90. 10.1016/j.jmva.2011.05.016

[B2] Armour C. Shevlin M. Elklit A. Mroczek D. (2012). A latent growth mixture modeling approach to PTSD symptoms in rape victims. Traumatology 18, 20–28. 10.1177/1534765610395627 22661909 PMC3365569

[B3] Asparouhov T. Muthén B. (2015). Structural equation models and mixture models with continuous nonnormal skewed distributions. Struct. Equ. Model Multidiscip. J. 23, 1–19. 10.1080/10705511.2014.947375

[B4] Bandalos D. L. (2013). The use of Monte Carlo studies in structural equation modeling research, in Structural Equation Modeling: A Second Course. eds Hancock G. R. Mueller R. O. (Charlotte, NC: Information Age), 625–666.

[B5] Bauer D. J. Curran P. J. (2003). Distributional assumptions of growth mixture models: implications for overextraction of latent trajectory classes. Psychol Methods 8, 338–363. 10.1037/1082-989X.8.3.338 14596495

[B6] Baumeister R. F. Tice D. M. (1988). Metatraits. J. Pers. 56, 571–598.

[B7] Bollen K. A. Curran P. J. (2006). Latent curve models: A structural equation perspective, in Wiley Series in Probability and Statistics (Hoboken, NJ: John Wiley & Sons, Inc.), 162–87. 10.1002/0471746096

[B8] Boomsma A. (1985). Nonconvergence, improper solutions, and starting values in LISREL maximum likelihood estimation. Psychometrika 50, 229–242. 10.1007/BF02294248

[B9] Brame B. Nagin D. S. Tremblay R. E. (2001). Developmental trajectories of physical aggression from school entry to late adolescence. J. Child Psychol. Psychiatry 42, 503–512. 10.1111/1469-7610.00744 11383966

[B10] Brame R. Nagin D. S. Wasserman L. (2006). Exploring some analytical characteristics of finite mixture models. J. Quant. Criminol. 22, 31–59. 10.1007/s10940-005-9001-8

[B11] Brandt H. Umbach N. Kelava A. (2015). The standardization of linear and nonlinear effects in direct and indirect applications of structural equation mixture models for normal and nonnormal data. Front. Psychol. 6:1813. 10.3389/fpsyg.2015.01813 26648886 PMC4663265

[B12] Brose A. Schmiedek F. Lövdén M. Lindenberger U. (2012). Daily variability in working memory is coupled with negative affect: the role of attention and motivation. Emotion 12, 605–617. 10.1037/a0024436 21787075

[B13] Chen F. Bollen K. A. Paxton P. Curran P. J. Kirby J. B. (2001). Improper solutions in structural equation models: causes, consequences, and strategies. Sociol. Methods Res. 29, 468–508. 10.1177/0049124101029004003

[B14] Colder C. R. Mehta P. Balanda K. Campbell R. T. Mayhew K. Stanton W. R.. (2001). Identifying trajectories of adolescent smoking: an application of latent growth mixture modeling. Health Psychol. 20, 127–135. 10.1037/0278-6133.20.2.127 11315730

[B15] Cole D. A. Martin N. C. Steiger J. H. (2005). Empirical and conceptual problems with longitudinal trait-state models: introducing a trait-state-occasion model. Psychol. Methods 10, 3–20. 10.1037/1082-989X.10.1.3 15810866

[B16] Courvoisier D. S. (2006). Unfolding the Constituents of Psychological Scores syDevelopment and Application of Mixture and Multitrait-Multimethod LST Models. Availabe online at: http://nbn-resolving.de/urn:nbn:ch:unige-4478

[B17] Courvoisier D. S. Eid M. Nussbeck F. W. (2007). Mixture distribution latent state-trait analysis: basic ideas and applications. Psychol. Methods 12, 80–104. 10.1037/1082-989X.12.1.80 17402813

[B18] Delucchi K. L. Bostrom A. (2004). Methods for analysis of skewed data distributions in psychiatric clinical studies: working with many zero values. Am. J. Psychiatry 161, 1159–1168. 10.1176/appi.ajp.161.7.1159 15229044

[B19] Diallo T. M. Morin A. J. Lu H. (2016). Impact of misspecifications of the latent variance–covariance and residual matrices on the class enumeration accuracy of growth mixture models. Struct. Equ. Model Multidiscip. J. 23, 507–531. 10.1080/10705511.2016.1169188

[B20] Dolan C. V. Schmittmann V. D. Lubke G. H. Neale M. C. (2005). Regime switching in the latent growth curve mixture model. Struct. Equ. Model. 12, 94–119. 10.1207/s15328007sem1201_5

[B21] Dolan C. V. van der Maas H. L. J. (1998). Fitting multivariage normal finite mixtures subject to structural equation modeling. Psychometrika 63, 227–253.

[B22] Eid M. (1996). Longitudinal confirmatory factor analysis for polytomous item responses: Model definition and model selection on the basis of stochastic measurement theory. Methods Psychol. Res. 1, 65–85.

[B23] Eid M. Diener E. (2004). Global judgments of subjective well-being: situational variability and long-term stability. Soc. Indic. Res. 65, 245–277. 10.1023/B:SOCI.0000003801.89195.bc

[B24] Eid M. Geiser C. Nussbeck F. (2008). Neuere psychometrische Ansätze der Veränderungsmessung. Z. Für Psychiatr. Psychol. Psychother. 56, 181–189. 10.1024/1661-4747.56.3.181

[B25] Eid M. Holtmann J. Santangelo P. Ebner-Priemer U. (2017). On the definition of latent-state-trait models with autoregressive effects. Eur. J. Psychol. Assess. 33, 285–295. 10.1027/1015-5759/a000435

[B26] Eid M. Kutscher T. (2014). Statistical models for analyzing stability and change in happiness, in Stability of Happiness: Theory and Evidence on Whether Happiness Can Change, eds Sheldon K. Lucas R. E. (London: Academic Press), 263–297. 10.1016/B978-0-12-411478-4.00013-8

[B27] Eid M. Luhmann M. (2012). Models of measurement of persons in situations, in Encyclopedia of the Sciences of Learning, ed Seel N. M. (New York, NY: Springer), 2323–2325.

[B28] Eid M. Schneider C. Schwenkmezger P. (1999). Do you feel better or worse? The validity of perceived deviations of mood states from mood traits. Eur. J. Personal 13, 283–306. 10.1002/(SICI)1099-0984(199907/08)13:4<283::AID-PER341>3.0.CO;2-0

[B29] Enders C. K. Bandalos D. L. (2001). The relative performance of full information maximum likelihood estimation for missing data in structural equation models. Struct. Equ. Model 8, 430–457. 10.1207/S15328007SEM0803_5

[B30] Espy K. A. Fang H. Charak D. Minich N. Taylor H. G. (2009). Growth mixture modeling of academic achievement in children of varying birth weight risk. Neuropsychology 23, 460–474. 10.1037/a0015676 19586210 PMC2776698

[B31] Funder D. C. (2008). Persons, situations and person-situation interactions, in Handbook of Personality: Theory and Research, 3rd Edn., eds John O. P. Robins R. W. Pervin L. A. (New York, NY: The Guildford Press), 568–80.

[B32] Geiser C. Lockhart G. (2012). A comparison of four approaches to account for method effects in latent state–trait analyses. Psychol. Methods 17, 255–283. 10.1037/a0026977 22309958 PMC3368086

[B33] Gottfredson N. C. Bauer D. J. Baldwin S. A. (2014). Modeling change in the presence of nonrandomly missing data: evaluating a shared parameter mixture model. Struct. Equ. Model Multidiscip. J. 21, 196–209. 10.1080/10705511.2014.882666 PMC4084916 25013354

[B34] Greenbaum P. E. Del Boca F. K. Darkes J. Wang C.-P. Goldman M. S. (2005). Variation in the drinking trajectories of freshmen college students. J. Consult. Clin. Psychol. 73, 229–238. 10.1037/0022-006X.73.2.229 15796630

[B35] Hallquist M. N. Lenzenweger M. F. (2013). Identifying latent trajectories of personality disorder symptom change: growth mixture modeling in the longitudinal study of personality disorders. J. Abnorm. Psychol. 122, 138–155. 10.1037/a0030060 23231459 PMC3570677

[B36] Heidemeier H. Göritz A. S. (2016). The instrumental role of personality traits: using mixture structural equation modeling to investigate individual differences in the relationships between the Big Five traits and life satisfaction. J. Happiness Stud. 17, 2595–2612. 10.1007/s10902-015-9708-7

[B37] Henson J. M. Reise S. P. Kim K. H. (2007). Detecting mixtures from structural model differences using latent variable mixture modeling: a comparison of relative model fit statistics. Struct. Equ. Model Multidiscip. J. 14, 202–226. 10.1080/10705510709336744

[B38] Herrmann A. Hahn C. H. Johnson M. D. Huber F. (2002). Capturing customer heterogeneity using a finite mixture PLS approach. Schmalenbach Bus. Rev. 54, 243–269. Availabe online at: https://scholarship.sha.cornell.edu/articles/697

[B39] Hipp J. R. Bauer D. J. (2006). Local solutions in the estimation of growth mixture models. Psychol. Methods 11, 36–53. 10.1037/1082-989X.11.1.36 16594766

[B40] Hix-Small H. Duncan T. E. Duncan S. C. Okut H. (2004). A multivariate associative finite growth mixture modeling approach examining adolescent alcohol and marijuana use. J. Psychopathol. Behav. Assess. 26, 255–270. 10.1023/B:JOBA.0000045341.56296.fa

[B41] Ho H. J. Lin T. I. Chang H. H. Haase S. B. Huang S. Pyne S. (2012). Parametric modeling of cellular state transitions as measured with flow cytometry. BMC Bioinformatics 13 (Suppl. 5):S5. 10.1186/1471-2105-13-S5-S5 22537009 PMC3358665

[B42] Ho R. T. Fong T. C. Cheung I. K. (2014). Cancer-related fatigue in breast cancer patients: factor mixture models with continuous non-normal distributions. Qual. Life Res. 23, 2909–2916. 10.1007/s11136-014-0731-7 24899547

[B43] Hoeksma J. B. Kelderman H. (2006). On growth curves and mixture models. Infant Child Dev. 15, 627–634. 10.1002/icd.483

[B44] Holtmann J. Koch T. Lochner K. Eid M. (2016). A comparison of ML, WLSMV, and Bayesian methods for multilevel structural equation models in small samples: a simulation study. Multivar. Behav. Res. 51, 661–680. 10.1080/00273171.2016.1208074 27594086

[B45] Jagodzinski W. Kühnel S. M. Schmidt P. (1987). Is there a “Socratic effect” in nonexperimental panel studies? Consistency of an attitude towards guestworkers. Sociol. Methods Res. 15, 259–302. 10.1177/0049124187015003004

[B46] Jedidi K. Jagpal H. S. DeSarbo W. S. (1997). Finite-mixture structural equation models for response-based segmentation and unobserved heterogeneity. Mark Sci. 16, 39–59. 10.1287/mksc.16.1.39

[B47] Jonas K. G. Markon K. E. (2013). A model of psychosis and its relationship with impairment. Soc. Psychiatry Psychiatr. Epidemiol. 48, 1367–1375. 10.1007/s00127-012-0642-2 23306423

[B48] Kenny D. A. McCoach D. B. (2003). Effect of the number of variables on measures of fit in structural equation modeling. Struct. Equ. Model Multidiscip. J. 10, 333–351. 10.1207/s15328007sem1003_1

[B49] Kenny D. A. Zautra A. (1995). The trait-state-error model for multiwave data. J. Consult. Clin. Psychol. 63, 52–59. 10.1037/0022-006X.63.1.52 7896990

[B50] Khoo S.-T. West S. G. Wu W. Kwok O.-M. (2006). Longitudinal methods, in Handbook of Multimethod Measurement in Psychology, eds Eid M. Diener E. (Washington, DC: American Psychological Association), 301–317.

[B51] Kim M. Vermunt J. Bakk Z. Jaki T. Van Horn M. L. (2016). Modeling predictors of latent classes in regression mixture models. Struct. Equ. Model Multidiscip. J. 23, 601–614. 10.1080/10705511.2016.1158655 PMC6777571 31588168

[B52] Kim S.-Y. (2014). Determining the number of latent classes in single-and multiphase growth mixture models. Struct. Equ. Model. Multidiscip. J. 21, 263–279. 10.1080/10705511.2014.882690 PMC3979564 24729675

[B53] Ko H.-J. Hooker K. Geldhof G. J. McAdams D. P. (2016). Longitudinal purpose in life trajectories: examining predictors in late midlife. Psychol. Aging 31, 693–698. 10.1037/pag0000093 27684106

[B54] Kooken J. (2015). Modeling Heterogeneity in Growth Mixture Models: A Case Study of Model Selection Using Direct Behavior Rating. Available online at: http://digitalcommons.uconn.edu/dissertations/961/

[B55] Koss K. J. George M. R. Davies P. T. Cicchetti D. Cummings E. M. Sturge-Apple M. L. (2013). Patterns of children's adrenocortical reactivity to interparental conflict and associations with child adjustment: a growth mixture modeling approach. Dev. Psychol. 49, 317–326. 10.1037/a0028246 22545835 PMC3819209

[B56] Kuppens P. Oravecz Z. Tuerlinckx F. (2010). Feelings change: Accounting for individual differences in the temporal dynamics of affect. J. Pers. Soc. Psychol. 99, 1042–1060. 10.1037/a0020962 20853980

[B57] Lee S. McLachlan G. J. (2014). Finite mixtures of multivariate skew t-distributions: Some recent and new results. Stat. Comput. 24, 181–202. 10.1007/s11222-012-9362-4

[B58] Lee S.-Y. Song X.-Y. (2003). Maximum likelihood estimation and model comparison for mixtures of structural equation models with ignorable missing data. J. Classif. 20, 221–255. 10.1007/s00357-003-0013-5

[B59] Legler J. M. Davis W. W. Potosky A. L. Hoffman R. M. (2004). Latent variable modelling of recovery trajectories: sexual function following radical prostatectomy. Stat. Med. 23, 2875–2893. 10.1002/sim.1864 15344192

[B60] Leite W. L. (2007). A comparison of latent growth models for constructs measured by multiple items. Struct. Equ. Model Multidiscip. J. 14, 581–610. 10.1080/10705510701575438

[B61] Li F. Duncan T. E. Duncan S. C. Hops H. (2001). Piecewise growth mixture modeling of adolescent alcohol use data. Struct. Equ. Model. 8, 175–204. 10.1207/S15328007SEM0802_2 11327186

[B62] Li L. Hser Y.-I. (2011). On inclusion of covariates for class enumeration of growth mixture models. Multivar. Behav. Res. 46, 266–302. 10.1080/00273171.2011.556549 23904664 PMC3726037

[B63] Lin H. Turnbull B. W. McCulloch C. E. Slate E. H. (2002). Latent class models for joint analysis of longitudinal biomarker and event process data: application to longitudinal prostate-specific antigen readings and prostate cancer. J. Am. Stat. Assoc. 97, 53–65. 10.1198/016214502753479220

[B64] Lin T.-I. Wu P. H. McLachlan G. J. Lee S. X. (2015). A robust factor analysis model using the restricted skew-t distribution. Test 24, 510–531. 10.1007/s11749-014-0422-2

[B65] Litson K. Geiser C. Burns G. L. Servera M. (2017). Examining trait\ times method interactions using mixture distribution multitrait–multimethod models. Struct. Equ. Model Multidiscip. J. 24, 31–51. 10.1080/10705511.2016.1238307 PMC5624226 28983185

[B66] Liu M. (2011). Using Latent Profile Models and Unstructured Growth Mixture Models to Assess the Number Of Latent Classes In Growth Mixture Modeling. Available online at: https://search.proquest.com/docview/902911563?accountid=11004.

[B67] Lo Y. Mendell N. R. Rubin D. B. (2001). Testing the number of components in a normal mixture. Biometrika 88, 767–778. 10.1093/biomet/88.3.767

[B68] Lubke G. H. Muthén B. (2005). Investigating population heterogeneity with factor mixture models. Psychol. Methods 10, 21–39. 10.1037/1082-989X.10.1.21 15810867

[B69] Lubke G. H. Muthén B. O. (2007). Performance of factor mixture models as a function of model size, covariate effects, and class-specific parameters. Struct. Equ. Model. 14, 26–47. 10.1080/10705510709336735

[B70] Lubke G. Tueller S. (2010). Latent class detection and class assignment: a comparison of the MAXEIG taxometric procedure and factor mixture modeling approaches. Struct. Equ. Model. 17, 605–628. 10.1080/10705511.2010.510050 24648712 PMC3955757

[B71] Marsh H. W. Hau K.-T. Balla J. R. Grayson D. (1998). Is more ever too much? The number of indicators per factor in confirmatory factor analysis. Multivar. Behav. Res. 33, 181–220. 26771883 10.1207/s15327906mbr3302_1

[B72] Martin D. P. von Oertzen T. (2015). Growth mixture models outperform simpler clustering algorithms when detecting longitudinal heterogeneity, even with small sample sizes. Struct. Equ. Model Multidiscip. J. 22, 264–275. 10.1080/10705511.2014.936340

[B73] McArdle J. J. Prindle J. J. (2008). A latent change score analysis of a randomized clinical trial in reasoning training. Psychol. Aging 23, 702–719. 10.1037/a0014349 19140642

[B74] McIntyre H. H. (2011). Investigating response styles in self-report personality data via a joint structural equation mixture modeling of item responses and response times. Personal Individ. Differ. 50, 597–602. 10.1016/j.paid.2010.12.001

[B75] McLachlan G. Peel D. (2004). Finite Mixture Models. New York, NY: John Wiley and Sons.

[B76] Meredith W. (1993). Measurement invariance, factor analysis and factorial invariance. Psychometrika 58, 525–543. 10.1007/BF02294825

[B77] Micceri T. (1989). The Unicorn, the normal curve, and other improbable creatures. Psychol. Bull. 105, 156–166. 10.1037/0033-2909.105.1.156

[B78] Miettunen J. Nordström T. Kaakinen M. Ahmed A. (2016). Latent variable mixture modeling in psychiatric research–a review and application. Psychol. Med. 46, 457–467. 10.1017/S0033291715002305 26526221

[B79] Molenaar P. C. M. Campbell C. G. (2009). The new person-specific paradigm in psychology. Curr. Dir. Psychol. Sci. 18, 112–117. 10.1111/j.1467-8721.2009.01619.x

[B80] Molenaar P. C. M. Huizenga H. M. Nesselroade J. R. (2003). The relationship between the structure of interindividual and intraindividual variability: a theoretical and empirical vindication of developmental systems theory, in Understanding Human Development, eds Staudinger U. M. Lindenberger U. (Boston, MA: Springer), 339–360.

[B81] Morgan G. B. Beaujean A. A. (2014). An investigation of growth mixture models for studying the Flynn Effect. J. Intell. 2, 156–179. 10.3390/jintelligence2040156

[B82] Muthén B. Asparouhov T. (2014). Non-normal growth mixture modeling. Prev. Sci. Methodol. Group. 34, 1041–1058. Available online at: https://www.statmodel.com/download/2014MayPSMGtalk.pdf 25504555 10.1002/sim.6388

[B83] Muthén B. (2001). Latent variable mixture modeling, in New Developments and Techniques in Structural Equation Modeling, eds Marcoulides G. A. Schumacker R. E. (Mahwah, NJ: Lawrence Erlbaum Associates), 1–33.

[B84] Muthén B. Asparouhov T. (2015). Growth mixture modeling with non-normal distributions. Stat. Med. 34, 1041–1058. 10.1002/sim.6388 25504555

[B85] Muthén L. K. Muthén B. O. (1998–2017). MPlus User's Guide, 8th Edn. Los Angeles, CA: Muthén Muthén.

[B86] Muthén L. K. Muthén B. O. (2002). How to use a Monte Carlo study to decide on sample size and determine power. Struct. Equ. Model. 9, 599–620. 10.1207/S15328007SEM0904_8

[B87] Nylund K. L. Asparouhov T. Muthén B. O. (2007). Deciding on the number of classes in latent class analysis and growth mixture modeling: a Monte Carlo simulation study. Struct. Equ. Model. 14, 535–569. 10.1080/10705510701575396

[B88] Petras H. Schaeffer C. M. Ialongo N. Hubbard S. Muthén B. Lambert S. F. (2004). When the course of aggressive behavior in childhood does not predict antisocial outcomes in adolescence and young adulthood: An examination of potential explanatory variables. Dev. Psychopathol. 16, 919–941. 10.1017/S0954579404040076 15704821

[B89] Peugh J. L. Strotman D. McGrady M. Rausch J. Kashikar-Zuck S. (2017). Beyond intent to treat (ITT): a complier average causal effect (CACE) estimation primer. J. Sch. Psychol. 60, 7–24. 10.1016/j.jsp.2015.12.006 28164801

[B90] Pinquart M. Schindler I. (2007). Changes of life satisfaction in the transition to retirement: a latent-class approach. Psychol. Aging 22, 442–455. 10.1037/0882-7974.22.3.442 17874946

[B91] Ram N. Grimm K. J. (2009). Growth mixture modeling: a method for identifying differences in longitudinal change among unobserved groups. Int. J. Behav. Dev. 33, 565–576. 10.1177/0165025409343765 23885133 PMC3718544

[B92] R Core Team (2015). R: A Language and Environment for Statistical Computing. Vienna, Austria Available online at: https://www.R-project.org/.

[B93] Reinecke J. (2006). Longitudinal analysis of adolescents' deviant and delinquent behavior. Methodology 2, 100–112. 10.1027/1614-2241.2.3.100

[B94] Röcke C. Brose A. (2013). Intraindividual variability and stability of affect and well-being: short-term and long-term change and stabilization processes. GeroPsych. 26, 185–99. 10.1037/a0039341

[B95] Schermelleh-Engel K. Keith N. Moosbrugger H. Hodapp V. (2004). Decomposing person and occasion-specific effects: An extension of latent state-trait (LST) theory to hierarchical LST models. Psychol. Methods. 9, 198–219. 10.1037/1082-989X.9.2.198 15137889

[B96] Schmitt M. (2000). Mother–Daughter attachement and family cohesion: Single-and multi-construct latent state-trait models of current and retrospective perceptions. Eur. J. Psychol. Assess. 16, 115–124. 10.1027//1015-5759.16.2.115

[B97] Steyer R. Ferring D. Schmitt M. J. (1992). States and traits in psychological assessment. Eur. J. Psychol. Assess. 8, 79–98.

[B98] Steyer R. Mayer A. Geiser C. Cole D. A. (2015). A theory of states and traits-revised. Annu. Rev. Clin. Psychol. 11, 71–98. 10.1146/annurev-clinpsy-032813-153719 25062476

[B99] Steyer R. Schmitt M. Eid M. (1999). Latent state–trait theory and research in personality and individual differences. Eur. J. Personal 13, 389–408. 10.1002/(SICI)1099-0984(199909/10)13:5<389::AID-PER361>3.0.CO;2-A

[B100] Stoolmiller M. Kim H. K. Capaldi D. M. (2005). The course of depressive symptoms in men from early adolescence to young adulthood: Identifying latent trajectories and early predictors. J. Abnorm. Psychol. 114, 331–345. 10.1037/0021-843X.114.3.331 16117571 PMC1698962

[B101] Tolvanen A. (2007). Latent Growth Mixture Modeling: A Simulation Study. Jyväskylä: University of Jyväskylä.

[B102] Tueller S. J. Drotar S. Lubke G. H. (2011). Addressing the problem of switched class labels in latent variable mixture model simulation studies. Struct. Equ. Model. 18, 110–131. 10.1080/10705511.2011.534695

[B103] Tueller S. Lubke G. H. (2010). Evaluation of structural equation mixture models: parameter estimates and correct class assignment. Struct. Equ. Model. 17, 165–192. 10.1080/10705511003659318 20582328 PMC2890304

[B104] Ulitzsch E. Holtmann J. Schultze M. Eid M. (2017). Comparing multilevel and classical confirmatory factor analysis parameterizations of multirater data: a Monte Carlo simulation study. Struct. Equ. Model Multidiscip. J. 24, 80–103. 10.1080/10705511.2016.1251846

[B105] Usami S. (2014). Performance of information criteria for model selection in a latent growth curve mixture model. J. Jpn. Soc. Comput. Stat. 27, 17–48. 10.5183/jjscs.1309001_207

[B106] Van Horn M. L. Jaki T. Masyn K. Ramey S. L. Smith J. A. Antaramian S. (2009). Assessing differential effects: applying regression mixture models to identify variations in the influence of family resources on academic achievement. Dev. Psychol. 45, 1298–1313. 10.1037/a0016427 19702393 PMC4492532

[B107] Wall M. M. Guo J. Amemiya Y. (2012). Mixture factor analysis for approximating a nonnormally distributed continuous latent factor with continuous and dichotomous observed variables. Multivar. Behav. Res. 47, 276–313. 10.1080/00273171.2012.658339 26734851 PMC9990906

[B108] Wang J. C. Liu W. C. Chatzisarantis N. L. Lim C. B. (2010). Influence of perceived motivational climate on achievement goals in physical education: a structural equation mixture modeling analysis. J. Sport Exerc. Psychol. 32, 324–338. 10.1123/jsep.32.3.324 20587821

[B109] Wiesner M. Windle M. (2004). Assessing covariates of adolescent delinquency trajectories: A latent growth mixture modeling approach. J. Youth Adolesc. 33, 431–442. 10.1023/B:JOYO.0000037635.06937.13

