# Supplementary material for: Skew t Mixture Latent State-Trait Analysis: A Monte Carlo Simulation Study on Statistical Performance
Source: Front Psychol. 2018 Aug 2;9:1323. doi: 10.3389/fpsyg.2018.01323 (PMC6083219; doi:10.3389/fpsyg.2018.01323)
Supplement: Supplementary file 1 [file Table_1.docx]

Supplementary Material

Skew *t* Mixture Latent State-Trait Analysis: A Monte Carlo Simulation Study on Statistical Performance

**Louisa Hohmann*, Jana Holtmann, Michael Eid,**

***Correspondence:** Louisa Hohmann: [louisa.hohmann@fu-berlin.de](mailto:louisa.hohmann@fu-berlin.de)

**Table S 1.** *Model Parameters for the Simulation Design*

| Parameter | Class 1 | Class 2 |
| --- | --- | --- |
| Trait loadings $\lambda_{T2c}$ | 1.05 | 0.94 |
| Intercepts $\alpha_{2c}$ | -1.02 | 0.70 |
| $\mu_{T_{c}}$ | 14.32 | 17.79 |
| $Var\left( O_{c} \right)$ | 9.97 | 0.78 |
| $\sigma_{T_{c}}^{2}$ | 1.59 | 0.64 |
| $Var\left( {IST}_{2c} \right)$ | 0.63 | 0.33 |
| $Var\left( E_{1c} \right)$ | 2.30 | 1.06 |
| $Var\left( E_{jc} \right)$ for $j>1$ | 1.42 | 0.64 |
| $\nu_{c}$ | 5.00 | 5.00 |
| $\delta_{Tc}$ | 2.80 / 6.00 | 2.80 / 6.00 |
| Class probability | 76 % (Logit = 1.15) | 24 % |
| *Note.* Due to measurement invariance settings, trait loadings and intercepts were equal across time, and residual variances were equal across time for *j* > 1. *c* = index for class; $E_{jc}$ = residual variable; *j* = index for occasions; *O* = latent occasion-specific variable; *IST_2c_* = latent indicator-specific residual trait variable for the second indicator; *T_c_* = latent trait variable; $\nu_{c}$= degrees of freedom parameter; $\delta_{Tc}$ = skewness parameter for *T_c_*; $\sigma_{T_{c}}^{2}$ = scale parameter for *T_c_*;$\mu_{T_{c}}$ = location parameter for *T_c._* | | |

Table S 2. *Number of Converged and Included Replications per Condition*

| Models with mild skewness ($\delta_{Tc}=2.8)$ | | | | | | | | | | Models with high skewness *(*$\delta_{Tc}=6)$ | | | | | | | | | | | |  |
| --- | --- | --- | --- | --- | --- | --- | --- | --- | --- | --- | --- | --- | --- | --- | --- | --- | --- | --- | --- | --- | --- | --- |
| Condition | | Number of replications | | Exclusion criteria fulfilled | | | | | | Condition | | | Number of replications | | Exclusion criteria fulfilled | | | | | | |  |
| *j* | *N* | *conv.* | *incl.* | *CS* | *sing.* | *SE* | $\nu_{1}$ | $\nu_{2}$ | *SP* | *j* | *N* | *conv.* | | *incl.* | | *CS* | *sing.* | *SE* | $\nu_{1}$ | $\nu_{2}$ | *SP* | |
| 2 | 125 | 471 | 9 | 50 | 456 | 78 | 8 | 53 | 0 | 2 | 125 | 479 | | 7 | | 65 | 466 | 102 | 11 | 43 | 0 | |
| 2 | 250 | 478 | 33 | 38 | 428 | 31 | 1 | 71 | 1 | 2 | 250 | 480 | | 18 | | 48 | 451 | 37 | 3 | 53 | 1 | |
| 2 | 500 | 492 | 112 | 18 | 357 | 13 | 0 | 49 | 3 | 2 | 500 | 484 | | 85 | | 17 | 385 | 20 | 0 | 39 | 4 | |
| 2 | 1,000 | 494 | 237 | 3 | 235 | 3 | 0 | 21 | 6 | 2 | 1,000 | 494 | | 175 | | 6 | 305 | 2 | 0 | 19 | 3 | |
| 3 | 125 | 490 | 82 | 18 | 397 | 36 | 4 | 53 | 1 | 3 | 125 | 488 | | 40 | | 38 | 435 | 47 | 4 | 53 | 2 | |
| 3 | 250 | 497 | 169 | 13 | 294 | 10 | 0 | 53 | 1 | 3 | 250 | 494 | | 103 | | 13 | 371 | 10 | 0 | 50 | 4 | |
| 3 | 500 | 497 | 265 | 0 | 206 | 1 | 0 | 33 | 2 | 3 | 500 | 500 | | 206 | | 5 | 276 | 2 | 0 | 22 | 2 | |
| 3 | 1,000 | 500 | 391 | 0 | 104 | 0 | 0 | 4 | 1 | 3 | 1,000 | 499 | | 309 | | 0 | 189 | 0 | 0 | 1 | 1 | |
| 4 | 125 | 495 | 117 | 12 | 352 | 13 | 0 | 54 | 1 | 4 | 125 | 496 | | 84 | | 13 | 390 | 22 | 0 | 52 | 1 | |
| 4 | 250 | 495 | 252 | 0 | 214 | 1 | 0 | 41 | 4 | 4 | 250 | 498 | | 188 | | 1 | 294 | 2 | 0 | 34 | 4 | |
| 4 | 500 | 499 | 361 | 0 | 128 | 0 | 0 | 10 | 2 | 4 | 500 | 498 | | 281 | | 0 | 213 | 0 | 0 | 9 | 1 | |
| 4 | 1,000 | 500 | 431 | 0 | 68 | 0 | 0 | 1 | 0 | 4 | 1,000 | 500 | | 375 | | 0 | 121 | 0 | 0 | 0 | 4 | |
| 5 | 125 | 494 | 183 | 5 | 282 | 6 | 0 | 53 | 0 | 5 | 125 | 492 | | 127 | | 7 | 349 | 5 | 0 | 49 | 0 | |
| 5 | 250 | 498 | 295 | 0 | 182 | 0 | 0 | 28 | 0 | 5 | 250 | 497 | | 222 | | 0 | 265 | 0 | 0 | 22 | 1 | |
| 5 | 500 | 498 | 405 | 0 | 90 | 0 | 0 | 4 | 0 | 5 | 500 | 499 | | 319 | | 0 | 176 | 0 | 0 | 3 | 2 | |
| 5 | 1,000 | 500 | 466 | 0 | 33 | 0 | 0 | 1 | 0 | 5 | 1,000 | 500 | | 398 | | 0 | 99 | 0 | 0 | 0 | 3 | |
| 6 | 125 | 496 | 214 | 1 | 258 | 3 | 0 | 43 | 2 | 6 | 125 | 494 | | 136 | | 3 | 342 | 3 | 0 | 40 | 0 | |
| 6 | 250 | 496 | 337 | 0 | 144 | 0 | 0 | 19 | 1 | 6 | 250 | 498 | | 251 | | 0 | 237 | 0 | 0 | 17 | 2 | |
| 6 | 500 | 498 | 422 | 0 | 72 | 0 | 0 | 5 | 1 | 6 | 500 | 500 | | 342 | | 0 | 157 | 0 | 0 | 2 | 0 | |
| 6 | 1,000 | 500 | 474 | 0 | 26 | 0 | 0 | 0 | 0 | 6 | 1,000 | 500 | | 417 | | 0 | 83 | 0 | 0 | 0 | 0 | |
| *Note.* For some replications more than one exclusion criterion was fulfilled; conv. = converged; CS = class separation insufficient; incl. = included; *j* = number of occasions; SE = standard error warning message; sing. = singularity of the information matrix; SP = saddle point; $\nu_{1}$ = warning message for degrees of freedom parameter in class 1; $\nu_{2}$ = warning message for degrees of freedom parameter in class 2; $\delta_{Tc}$ = class-specific skewness parameter for the trait factor. | | | | | | | | | | | | | | | | | | | | | |  |

Table S 3. *Summary of the Results of the Simulation Study*

| Param. | *peb* | | | | | | *seb* | | | *MSE* | | | | 95% coverage | | |
| --- | --- | --- | --- | --- | --- | --- | --- | --- | --- | --- | --- | --- | --- | --- | --- | --- |
|  |  | No. of  cut-offs exceeded | | Tendency with increasing | | |  | No. of  cut-offs exceeded | |  | Tendency with increasing | | |  | No. of  cut-offs exceeded | |
| [population value] | General results | md | lrg | *j* | *N* | $\delta_{Tc}$ | General results | md | lrg | Range | *j* | *N* | $\delta_{Tc}$ | General results | <.91 | >.98 |
| Parameters referring to the skew *t*-distribution | | | | | | | | | | | | | | | | |
| $\nu_{1}$  [5] | Acceptable for most models with *N* ≥ 250 | 5 | 0 | > | > | s | Acceptable for most models with *j* > 4 | 7 | 1 | [0.12; 6.07] | > | > | s | Problems for $\delta_{Tc}=6$ | 0 | 7 |
| $\nu_{2}$  [5] | Acceptable for *j* ≥ 4 + *N* = 1,000; *j* ≥ 5 + *N* = 1,000;  $\delta_{Tc}=2.8$ *j* = 3 + *N* = 1,000 | 4 | 20 | > | > | > | Diffuse pattern | 3 | 22 | [0.67; 413261] | > | > | < | Problems for small *N* and small *j* + $\delta_{Tc}=6$ | 6 | 2 |
| $\delta_{T1}$  [2.8/ 6] | Acceptable for all models with *N* ≥ 250 or *j* = 6, biased downwards | 3 | 0 | > | > | > | Diffuse pattern | 13 | 7 | [0.08; 2.31] | > | > | s | Problems for small *j* (>.98) and high *j* + small *N* (<.91) | 6 | 3 |
| $\delta_{T2}$  [2.8/ 6] | Acceptable for *j* ≥ 5, *N* ≥ 500, biased downwards | 13 | 2 | > | > | > | Diffuse pattern | 19 | 8 | [0.10; 8.18] | > | > | < | Most problems for $\delta_{Tc}=6$ + small *j* | 0 | 4 |
| Logit  [1.15] | Only (downward) bias for small *j + N* + $\delta_{Tc}=2.8$ | 1 | 0 | > | > | s | Acceptable for *j* ≥ 5 + *N* ≥ 500 | 12 | 15 | [0.01; 0.15] | > | > | s | >.98 for *j* = 2,3 + small *N* | 0 | 2 |

| Param. | *peb* | | | | | | | | | | | | | | | | *seb* | | | | | | | *MSE* | | | | | | | | | | | 95% coverage | | | | | | | | | | | |
| --- | --- | --- | --- | --- | --- | --- | --- | --- | --- | --- | --- | --- | --- | --- | --- | --- | --- | --- | --- | --- | --- | --- | --- | --- | --- | --- | --- | --- | --- | --- | --- | --- | --- | --- | --- | --- | --- | --- | --- | --- | --- | --- | --- | --- | --- | --- |
|  |  | | No. of  cut-offs exceeded | | | | | | Tendency with increasing | | | | | | | |  | | | No. of  cut-offs exceeded | | | |  | Tendency with increasing | | | | | | | | | |  | | | | No. of  cut-offs exceeded | | | | | | | |
| [population value] | | General results | | md | | lrg | | *j* | | | | | *N* | | | $\delta_{Tc}$ | | General results | | | md | lrg | | Range | *j* | | | *N* | | | $\delta_{Tc}$ | | | | | | General results | | | <.91 | | | | >.98 | | |
| Scale parameters/ variances of the latent trait variables | | | | | | | | | | | | | | | | | | | | | | | | | | | | | | | | | | | | | | | | | | | | | |  |
| $\sigma_{T1}^{2}$  [1.59] | Acceptable for *j* ≥ 4 + *N* ≥ 500 and $\delta_{Tc}=2.8$ + *N* ≥ 250 | | 9 | | 9 | | | > | | | > | | | < | | | | | Acceptable for *j* ≥ 5 + *N* ≥ 500  and *j* = 4 + *N* = 1,000 | | 9 | | 13 | [0.26; 6.33] | | > | | | > | | | | < | - | | | | 0 | | | | 0 | | | | |
| $\sigma_{T2}^{2}$  [0.64] | Biased upwards in all models | | 2 | | 33 | | | > | | | > | | | < | | | | | Diffuse pattern | | 16 | | 12 | [0.32; 0.17] | | > | | | > | | | | < | Problems for small *N* and *j* | | | | 5 | | | | 0 | | | | |
| $Var({IST}_{21})$  [0.63] | | Biased downwards for *j* = 2 + *N* = 250 | 2 | | | | 0 | | | > | | D | | | D | | | | Acceptable for *j* ≥ 5, *N* ≥ 250 | | 11 | | 3 | [0.00; 0.12] | | | > | | | > | | s | | | | Problems with small *N* (<.91) or large *N* (>.98) | | | | | 5 | | 2 | |  |  |
| $Var({IST}_{22})$  [0.33] | | Biased upwards for small *j*, biased downwards for large *j* | 6 | | | | 1 | | | > | | D | | | D | | | | Acceptable for *j* ≥ 5, *N* ≥ 250 | | 5 | | 8 | [0.00; 0.10] | | | > | | | > | | s | | | | - | | | | | 0 | | 0 | |  |  |
| $Var(O_{1})$  [9.97] | | - | 0 | | | | 0 | | | - | | - | | | - | | | | Acceptable for *j* ≥ 5, *N* = 1,000 | | 11 | | 9 | [0.16; 4.52] | | | > | | | > | | < | | | | Problems with small *N* + small *j* | | | | | 4 | | 0 | |  |  |
| $Var(O_{2})$  [0.78] | | - | 0 | | | | 0 | | | - | | - | | | - | | | | Acceptable for *j* ≥ 3, *N* = 1,000 | | 15 | | 7 | [0.01; 0.19] | | | > | | | > | | s | | | | - | | | | | 0 | | 0 | |  |  |

| Param. | *peb* | | | | | | | | | | | | | | | | | | | | | *seb* | | | | | | | | | | | | | | | *MSE* | | | | | | | | | | | | | | 95% coverage | | | | | | | | | | |
| --- | --- | --- | --- | --- | --- | --- | --- | --- | --- | --- | --- | --- | --- | --- | --- | --- | --- | --- | --- | --- | --- | --- | --- | --- | --- | --- | --- | --- | --- | --- | --- | --- | --- | --- | --- | --- | --- | --- | --- | --- | --- | --- | --- | --- | --- | --- | --- | --- | --- | --- | --- | --- | --- | --- | --- | --- | --- | --- | --- | --- | --- |
|  |  | | | No. of  cut-offs exceeded | | | | | | Tendency with increasing | | | | | | | | | | | |  | | | | | No. of  cut-offs exceeded | | | | | | | | | |  | Tendency with increasing | | | | | | | | | | | | |  | | No. of  cut-offs exceeded | | | | | | | | |
| [population value] | | General results | | | md | lrg | | | | *j* | | | | | *N* | | | | $\delta_{Tc}$ | | | | General results | | | | | md | | | lrg | | | | | | Range | *j* | | | | *N* | | | | $\delta_{Tc}$ | | | | | | General results | | <.91 | | | | | >.98 | | |
| Residual Variances | | | | | | | | | | | | | | | | | | | | | | | | | | | | | | | | | | | | | | | | | | | | | | | | | | | | | | | | | | | |  |  |
| $Var\left( E_{11} \right)$  [2.30] | | - | | 0 | | | 0 | | | | > | | | D | | | | IA *N* | | | | | | Acceptable for $\delta_{TC}=2.8$ + *j* ≥ 3 + *N* ≥ 250 or *j* ≥ 3+ N = 1,000 | | | | 10 | | | | | | 3 | | | [0.02; 0.29] | | | | > | | | | > | | | s | | Most problems for $\delta_{Tc}=2.8$ + j = 6 | | | | | 4 | | | 1 | |  |  |
| $Var(E_{12})$  [1.06] | | | Upward bias solely for small conditions | | 2 | | | 0 | | | | > | | | | > | | | | D | | | | | Acceptable for $\delta_{TC}=2.8$ + j ≥ 3 + *N* ≥ 250 or *j* ≥ 3+ *N* = 1,000 | | | | 11 | | | 1 | | | [0.02; 0.30] | | | | > | | | | > | | | | s | | Diffuse pattern | | | | | 0 | | | 4 | | |  |  |
| $Var(E_{21})$  [1.42] | | | Upward bias solely for *j* = 4 + *N* = 125 | | 0 | | | 0 | | | | > | | | | D | | | | IA *N* | | | | | Acceptable for $\delta_{TC}=2.8$ + *j* ≥ 3 + *N* ≥ 250 or *j* ≥ 3+ *N* = 1,000 | | | | 11 | | | 3 | | | [0.00; 0.07] | | | | > | | | | > | | | | s | | Diffuse pattern | | | | | 0 | | | 3 | | |  |  |
| $Var(E_{22})$  [0.64] | | | Downward bias solely for *j* = 2 + *N* = 250 | | 2 | | | | 0 | | | | > | | | | D | | | | IA | | | | | Acceptable for *j* ≥ 4+ *N* = 1,000 | | | | 15 | | | 4 | | | [0.00; 0.09] | | | | > | | | | > | | | | s | | | Problems for $\delta_{Tc}=6$ + j = 2 or small N | | | | | 0 | | | 4 | |  |

| Param. | *peb* | | | | | | | | | | | | | *seb* | | | | | | | *MSE* | | | | | | | 95% coverage | | | | | | |
| --- | --- | --- | --- | --- | --- | --- | --- | --- | --- | --- | --- | --- | --- | --- | --- | --- | --- | --- | --- | --- | --- | --- | --- | --- | --- | --- | --- | --- | --- | --- | --- | --- | --- | --- |
|  |  | | | No. of  cut-offs exceeded | | | | Tendency with increasing | | | | | |  | | No. of  cut-offs exceeded | | | | |  | Tendency with increasing | | | | | |  | | No. of  cut-offs exceeded | | | | |
| [population value] | | General results | | | md | lrg | | *j* | | *N* | | $\delta_{Tc}$ | | General results | | md | | lrg | | | Range | *j* | | *N* | | $\delta_{Tc}$ | | | General results | | <.91 | | >.98 | |
| Scale parameters of the latent trait variables, intercepts and trait loadings | | | | | | | | | | | | | | | | | | | | | | | | | | | | | | | | | |  |
| $\mu_{T1}$  [14.32] | | | - | | 0 | | 0 | | - | | - | | - | | Acceptable for *j* = 6 + *N* ≥ 500 and *j* = 5 + *N* = 1,000 | | 15 | | 7 | [0.05; 1.65] | | | > | | > | | s | Problems for $\delta_{Tc}=2.8$ + small j + small N | | | | 3 | 0 | |
| $\mu_{T2}$  [17.79] | | | - | | 0 | | 0 | | - | | - | | - | | Diffuse pattern | | 16 | | 5 | [0.05; 5.56] | | | > | | > | | < | Problems for small N | | | | 5 | 2 | |
| $\alpha_{21}$  [-1.02] | | | Bias for small *N* | | 4 | | 0 | | D | | D | | > | | Acceptable for *j* ≥ 3 + *N* = 1,000 | | 12 | | 4 | [0.05; 6.80] | | | > | | > | | > | Problems for $\delta_{Tc}=6$ + small N or small j | | | | 1 | 1 | |
| $\alpha_{22}$  [0.70] | | | Bias for small *N* | | 9 | | 2 | | > | | D | | D | | Acceptable for j ≥ 5 + *N* ≥ 500; very close to $\lambda_{22}$ | | 9 | | 4 | [0.11; 9.47] | | | > | | > | | > | Problems for small *N* | | | | 2 | 0 | |
| $\lambda_{21}$  [1.05] | | | - | | 0 | | 0 | | - | | - | | - | | Acceptable for *j* = 5, *N* ≥ 500 | | 12 | | 3 | [0.00; 0.02] | | | > | | > | | s | - | | | | 0 | 0 | |
| $\lambda_{22}$  [0.94] | | | - | | 0 | | 0 | | - | | - | | - | | Acceptable for *j* = 6 and *j* ≥ 5 + *N* ≥ 250, very close to $\alpha_{22}$ | | 8 | | 4 | [0.00; 0.02] | | | > | | > | | s | Problems for $\delta_{Tc}=6$ | | | | 0 | 14 | |
| *Note.* Medium bias : absolute *peb*/ *seb* between 0.10 and 0.30. Large bias: absolute *peb/ seb* > 0.30. Coverage was considered acceptable between .91 and .98. *c* = index for class; D = diffuse pattern; *E_kc_* = residual variable at occasion *k*; IA = interaction; *IST_2c_* = latent indicator-specific trait variable for second indicator; *j* = occasions; lrg = large; md = medium; *MSE* = mean squared error; *N* = sample size*; O_c_* = latent occasion-specific variable; Param. = parameter; *peb* = parameter estimation bias; s = similar (mean *MSE* differences between conditions < 0.100*); seb* = standard error bias; *T*_c_ = latent trait variable$; \alpha_{2c}$ = intercept for the second indicator; $\lambda_{2c}$ = trait loading for the second indicator;  $\mu_{Tc}$ = location parameter for the latent trait variable; $\sigma_{Tc}^{2}$ = scale parameter for the latent trait variable; < = increasing tendency; > = decreasing tendency. | | | | | | | | | | | | | | | | | | | | | | | | | | | | | | | | | | |

Table S 4. *Bivariate Spearman’s ρ Correlations Between Absolute Parameter Estimation Bias Values of Different Parameters Across Conditions*

|  |  | |  |  |  | |  |  | |  | |  | |  |  | |  | | | | | Variances/ Scale parameters | | | | | | | | | |
| --- | --- | --- | --- | --- | --- | --- | --- | --- | --- | --- | --- | --- | --- | --- | --- | --- | --- | --- | --- | --- | --- | --- | --- | --- | --- | --- | --- | --- | --- | --- | --- |
| Param. | | $\nu_{1}$ | | $\nu_{2}$ | | $\alpha_{21}$ | | | $\alpha_{22}$ | | Logit | | $\mu_{T1}$ | | | $\mu_{T2}$ | | $\delta_{T1}$ | $\delta_{T2}$ | $\lambda_{21}$ | $\lambda_{22}$ | *E_11_* | *E_12_* | *E_21_* | *E_22_* | *IST_21_* | *IST_22_* | *O_1_* | *O_2_* | $\sigma_{T1}^{2}$ | $\sigma_{T2}^{2}$ |
| $\nu_{1}$ | | **1** | |  | |  | | |  | |  | |  | | |  | |  |  |  |  |  |  |  |  |  |  |  |  |  |  |
| $\nu_{2}$ | | .15 | | **1** | |  | | |  | |  | |  | | |  | |  |  |  |  |  |  |  |  |  |  |  |  |  |  |
| $\alpha_{21}$ | | .15 | | .14 | | **1** | | |  | |  | |  | | |  | |  |  |  |  |  |  |  |  |  |  |  |  |  |  |
| $\alpha_{22}$ | | .14 | | .18 | | .24 | | | **1** | |  | |  | | |  | |  |  |  |  |  |  |  |  |  |  |  |  |  |  |
| Logit | | .15 | | .14 | | .15 | | | .14 | | **1** | |  | | |  | |  |  |  |  |  |  |  |  |  |  |  |  |  |  |
| $\mu_{T1}$ | | .12 | | .14 | | .14 | | | .16 | | .17 | | **1** | | |  | |  |  |  |  |  |  |  |  |  |  |  |  |  |  |
| $\mu_{T2}$ | | .14 | | .17 | | .11 | | | .15 | | .17 | | .13 | | | **1** | |  |  |  |  |  |  |  |  |  |  |  |  |  |  |
| $\delta_{T1}$ | | .14 | | .16 | | .23 | | | .24 | | .14 | | **.56** | | | .07 | | **1** |  |  |  |  |  |  |  |  |  |  |  |  |  |
| $\delta_{T2}$ | | .15 | | .16 | | .18 | | | .20 | | .17 | | .12 | | | **.47** | | .17 | **1** |  |  |  |  |  |  |  |  |  |  |  |  |
| $\lambda_{21}$ | | .15 | | .14 | | **.96** | | | .25 | | .15 | | .14 | | | .10 | | .25 | .18 | **1** |  |  |  |  |  |  |  |  |  |  |  |
| $\lambda_{22}$ | | .14 | | .17 | | .25 | | | **.97** | | .14 | | .15 | | | .13 | | .26 | .21 | .27 | **1** |  |  |  |  |  |  |  |  |  |  |
| $Var(E_{11})$ | | .13 | | .13 | | .11 | | | .12 | | .12 | | .13 | | | .15 | | .13 | .13 | .10 | .12 | **1** |  |  |  |  |  |  |  |  |  |
| $Var(E_{12})$ | | .12 | | .13 | | .14 | | | .15 | | .13 | | .13 | | | .15 | | .12 | .15 | .13 | .14 | .15 | **1** |  |  |  |  |  |  |  |  |
| $Var(E_{21})$ | | .18 | | .15 | | .15 | | | .16 | | .15 | | .13 | | | .18 | | .15 | .16 | .15 | .16 | .19 | .15 | **1** |  |  |  |  |  |  |  |
| $Var(E_{22})$ | | .13 | | .17 | | .13 | | | .18 | | .16 | | .14 | | | .16 | | .14 | .16 | .13 | .18 | .13 | .16 | .18 | **1** |  |  |  |  |  |  |
| $Var({IST}_{21})$ | | .13 | | .15 | | .18 | | | .16 | | .15 | | .14 | | | .17 | | .14 | .16 | .17 | .15 | .14 | .14 | .16 | .18 | **1** |  |  |  |  |  |
| $Var({IST}_{22})$ | | .14 | | .17 | | .15 | | | .18 | | .15 | | .13 | | | .17 | | .13 | .15 | .14 | .17 | .12 | .13 | .16 | .19 | .17 | **1** |  |  |  |  |
| $Var(O_{1})$ | | .20 | | .15 | | .17 | | | .15 | | .20 | | .14 | | | .18 | | .15 | .17 | .17 | .14 | .16 | .13 | .24 | .15 | .17 | .17 | **1** |  |  |  |
| $Var(O_{2})$ | | .13 | | .14 | | .15 | | | .16 | | .26 | | .12 | | | .16 | | .12 | .15 | .15 | .16 | .12 | .14 | .17 | .21 | .17 | .20 | .18 | **1** |  |  |
| $\sigma_{T1}^{2}$ | | .11 | | .11 | | .05 | | | .10 | | .12 | | **.40** | | | .12 | | .28 | .09 | .04 | .09 | .07 | .13 | .11 | .13 | .12 | .11 | .11 | .11 | **1** |  |
| $\sigma_{T2}^{2}$ | | .11 | | .12 | | .05 | | | .07 | | .15 | | .10 | | | **.39** | | .05 | **.37** | .05 | .06 | .11 | .12 | .14 | .12 | .12 | .12 | .16 | .12 | .11 | **1** |
| *Note.* Bivariate correlations > .30 are printed in bold. *c* = index for class; *E_jc_* = residual variable at occasion *j* ; *IST_2c_* = latent indicator-specific trait variable for the second indicator ; *O_c_* = latent occasion-specific variable ; Param. = parameter; *T*_c_ = latent trait variable ; $\alpha_{2c}$ = intercept for the second indicator ; $\lambda_{2c}$ = trait loading for the second indicator ; $\nu_{c}$ = degrees of freedom parameter ; $\delta_{Tc}$= skewness parameter for the trait factor ; $\mu_{Tc}$ = location parameter for the latent trait variable; $\sigma_{Tc}^{2}$ = scale parameter for the latent trait variable. | | | | | | | | | | | | | | | | | | | | | | | | | | | | | | | |

**Table S 5.** *Bivariate Spearman’s ρ Correlations Between Absolute Standard Error Bias Values of Different Parameters Across Conditions*

|  |  | |  |  |  | |  |  | |  | |  | |  |  | |  | | | | | Variances/ Scale parameters | | | | | | | | | |
| --- | --- | --- | --- | --- | --- | --- | --- | --- | --- | --- | --- | --- | --- | --- | --- | --- | --- | --- | --- | --- | --- | --- | --- | --- | --- | --- | --- | --- | --- | --- | --- |
| Param. | | $\nu_{1}$ | | $\nu_{2}$ | | $\alpha_{21}$ | | | $\alpha_{22}$ | | Logit | | $\mu_{T1}$ | | | $\mu_{T2}$ | | $\delta_{T1}$ | $\delta_{T2}$ | $\lambda_{21}$ | $\lambda_{22}$ | *E_11_* | *E_12_* | *E_21_* | *E_22_* | *IST_21_* | *IST_22_* | *O_1_* | *O_2_* | $\sigma_{T1}^{2}$ | $\sigma_{T2}^{2}$ |
| $\nu_{1}$ | | **1** | |  | |  | | |  | |  | |  | | |  | |  |  |  |  |  |  |  |  |  |  |  |  |  |  |
| $\nu_{2}$ | | **.31** | | **1** | |  | | |  | |  | |  | | |  | |  |  |  |  |  |  |  |  |  |  |  |  |  |  |
| $\alpha_{21}$ | | .19 | | **.34** | | **1** | | |  | |  | |  | | |  | |  |  |  |  |  |  |  |  |  |  |  |  |  |  |
| $\alpha_{22}$ | | .19 | | **.31** | | .20 | | | **1** | |  | |  | | |  | |  |  |  |  |  |  |  |  |  |  |  |  |  |  |
| Logit | | .18 | | .27 | | .21 | | | .22 | | **1** | |  | | |  | |  |  |  |  |  |  |  |  |  |  |  |  |  |  |
| $\mu_{T1}$ | | .16 | | .24 | | .17 | | | .16 | | .17 | | **1** | | |  | |  |  |  |  |  |  |  |  |  |  |  |  |  |  |
| $\mu_{T2}$ | | .12 | | .22 | | .13 | | | .14 | | .18 | | .13 | | | **1** | |  |  |  |  |  |  |  |  |  |  |  |  |  |  |
| $\delta_{T1}$ | | .18 | | .27 | | .22 | | | .18 | | .20 | | **.62** | | | .14 | | **1** |  |  |  |  |  |  |  |  |  |  |  |  |  |
| $\delta_{T2}$ | | .16 | | .26 | | .18 | | | .14 | | .22 | | .15 | | | **.75** | | .18 | **1** |  |  |  |  |  |  |  |  |  |  |  |  |
| $\lambda_{21}$ | | .19 | | **.34** | | **.96** | | | .20 | | .21 | | .16 | | | .12 | | .21 | .17 | **1** |  |  |  |  |  |  |  |  |  |  |  |
| $\lambda_{22}$ | | .18 | | .30 | | .20 | | | **.96** | | .22 | | .15 | | | .13 | | .17 | .12 | .19 | **1** |  |  |  |  |  |  |  |  |  |  |
| $Var(E_{11})$ | | .18 | | .26 | | .17 | | | .17 | | .20 | | .16 | | | .12 | | .17 | .15 | .17 | .16 | **1** |  |  |  |  |  |  |  |  |  |
| $Var(E_{12})$ | | .17 | | .28 | | .16 | | | .19 | | .17 | | .13 | | | .11 | | .14 | .15 | .16 | .19 | .19 | **1** |  |  |  |  |  |  |  |  |
| $Var(E_{21})$ | | .18 | | .27 | | .19 | | | .19 | | .25 | | .16 | | | .12 | | .17 | .14 | .19 | .18 | .26 | .16 | **1** |  |  |  |  |  |  |  |
| $Var(E_{22})$ | | .18 | | .29 | | .18 | | | .21 | | .27 | | .14 | | | .14 | | .16 | .16 | .19 | .20 | .15 | .20 | .20 | **1** |  |  |  |  |  |  |
| $Var({IST}_{21})$ | | .17 | | .26 | | .22 | | | .19 | | .18 | | .15 | | | .12 | | .18 | .14 | .22 | .18 | .20 | .14 | .20 | .14 | **1** |  |  |  |  |  |
| $Var({IST}_{22})$ | | .17 | | .26 | | .17 | | | .24 | | .18 | | .13 | | | .13 | | .14 | .14 | .17 | .23 | .15 | .17 | .15 | .21 | .18 | **1** |  |  |  |  |
| $Var(O_{1})$ | | .20 | | .27 | | .21 | | | .20 | | **.36** | | .18 | | | .16 | | .19 | .20 | .20 | .19 | .27 | .14 | **.37** | .19 | .20 | .15 | **1** |  |  |  |
| $Var(O_{2})$ | | .18 | | .28 | | .23 | | | .19 | | **.43** | | .20 | | | .12 | | .21 | .14 | .23 | .19 | .17 | .19 | .20 | .24 | .18 | .19 | .24 | **1** |  |  |
| $\sigma_{T1}^{2}$ | | .16 | | .27 | | .18 | | | .18 | | .21 | | **.47** | | | .12 | | **.31** | .12 | .18 | .18 | .16 | .14 | .18 | .15 | .18 | .14 | .21 | .21 | **1** |  |
| $\sigma_{T2}^{2}$ | | .07 | | .07 | | .04 | | | .08 | | .17 | | .06 | | | **.38** | | .04 | **.40** | .04 | .08 | .10 | .07 | .09 | .09 | .05 | .08 | .17 | .07 | .07 | **1** |
| *Note.* Bivariate correlations > .30 are printed in bold. *c* = index for class; *E_jc_* = residual variable at occasion *j;* *IST_2c_* = latent indicator-specific trait variable for the second indicator; *O_c_* = latent occasion-specific variable; Param. = parameter; *T*_c_ = latent trait variable; $\alpha_{2c}$ = intercept for the second indicator; $\lambda_{2c}$ = trait loading for the second indicator; $\nu_{c}$ = degrees of freedom parameter; $\delta_{Tc}$= skewness parameter for the trait factor; $\mu_{Tc}$ = location parameter for the latent trait variable; $\sigma_{Tc}^{2}$ = scale parameter for the latent trait variable. | | | | | | | | | | | | | | | | | | | | | | | | | | | | | | | |

Table S 6. *Results for the Simulation Study for the Skew t Mixture Latent State-Trait Model with* $\boldsymbol{\delta}_{\boldsymbol{Tc}}\boldsymbol{=6}$*, 2 Occasions and 500 Observations*

| Parameter | Pop. | Estimate | *SD* | *SE* | *peb* | *seb* | 95% coverage | *MSE* | included reps | converged reps |
| --- | --- | --- | --- | --- | --- | --- | --- | --- | --- | --- |
| $\nu_{1}$ | 5.00 | 5.41 | 0.91 | 1.06 | 0.08 | **0.16** | 0.98 | 1.01 | 85 | 484 |
| $\nu_{2}$ | 5.00 | 27.27 | 94.22 | 38.04 | **4.45** | **-0.60** | 1.00 | 9372.38 | 85 | 484 |
| $\alpha_{21}$ | -1.02 | -1.02 | 0.69 | 0.80 | 0.00 | **0.16** | 1.00 | 0.47 | 85 | 484 |
| $\alpha_{22}$ | 0.70 | 0.84 | 1.55 | 1.66 | 0.20 | 0.07 | 0.906 | 2.42 | 85 | 484 |
| Logit | 1.15 | 1.14 | 0.34 | 0.62 | -0.01 | **0.83** | 0.99 | 0.12 | 85 | 484 |
| $\mu_{T1}$ | 14.32 | 14.44 | 0.59 | 0.83 | 0.01 | **0.41** | 0.94 | 0.36 | 85 | 484 |
| $\mu_{T2}$ | 17.79 | 17.89 | 0.98 | 1.97 | 0.01 | **1.01** | 0.98 | 0.97 | 85 | 484 |
| $\delta_{T1}$ | 6.00 | 5.97 | 0.75 | 1.07 | 0.00 | **0.42** | 0.98 | 0.57 | 85 | 484 |
| $\delta_{T2}$ | 6.00 | 5.58 | 1.26 | 2.47 | -0.07 | **0.97** | 0.99 | 1.76 | 85 | 484 |
| $\lambda_{21}$ | 1.05 | 1.05 | 0.04 | 0.04 | 0.00 | 0.18 | 0.98 | 0.00 | 85 | 484 |
| $\lambda_{22}$ | 0.94 | 0.93 | 0.07 | 0.07 | -0.01 | 0.08 | 1.00 | 0.00 | 85 | 484 |
| $Var(E_{11})$ | 2.30 | 2.43 | 0.30 | 0.43 | 0.06 | **0.43** | 0.96 | 0.11 | 85 | 484 |
| $Var(E_{12})$ | 1.06 | 1.03 | 0.42 | 0.51 | -0.03 | 0.23 | 0.96 | 0.18 | 85 | 484 |
| $Var(E_{21})$ | 1.42 | 1.50 | 0.21 | 0.31 | 0.05 | **0.44** | 0.99 | 0.05 | 85 | 484 |
| $Var(E_{22})$ | 0.64 | 0.57 | 0.25 | 0.37 | **-0.11** | **0.47** | 1.00 | 0.07 | 85 | 484 |
| $Var({IST}_{21})$ | 0.63 | 0.56 | 0.24 | 0.38 | **-0.12** | **0.60** | 0.98 | 0.06 | 85 | 484 |
| $Var({IST}_{22})$ | 0.33 | 0.45 | 0.30 | 0.50 | **0.37** | **0.69** | 0.94 | 0.10 | 85 | 484 |
| $Var(O_{1})$ | 9.97 | 10.18 | 1.16 | 2.12 | 0.02 | **0.83** | 0.87 | 1.39 | 85 | 484 |
| $Var(O_{2})$ | 0.78 | 0.77 | 0.36 | 0.66 | -0.02 | **0.82** | 0.96 | 0.13 | 85 | 484 |
| $\sigma_{T1}^{2}$ | 1.59 | 2.34 | 1.43 | 2.59 | **0.47** | **0.81** | 0.94 | 2.61 | 85 | 484 |
| $\sigma_{T2}^{2}$ | 0.64 | 2.93 | 3.20 | 7.99 | **3.58** | **1.50** | 0.88 | 15.47 | 85 | 484 |
| *Note*. Absolute *peb* and *seb* values exceeding the cut-offs for at least medium bias (> 0.10) are printed in bold. *c =* index for class; $\alpha_{2c}$= intercept for the second indicator; $\delta_{Tc}$= skewness *;*$E_{jc}$= residual variable for occasion *j*; Estimate = mean of estimates of all included replications; $IST_{2c}$ = indicator-specific trait factor*;*$\lambda_{2c}$ = trait loading*;MSE* = mean squared error;$O_{c}$= occasion-specific factor*;peb* = parameter estimation bias; Pop. = population value; reps = replications; *SD* = standard deviation of parameter estimates; *SE* = standard error; *seb* = standard error bias; *T*_c_ = latent trait variable*;* $\nu_{c}$= degrees of freedom parameter*;* $\mu_{Tc}$ = location parameter for the latent trait variable; $\sigma_{Tc}^{2}$ = scale parameter for the latent trait variable. | | | | | | | | | | |

Table S 7. *Results for the Simulation Study for the Skew t Mixture Latent State-Trait Model with* $\boldsymbol{\delta}_{\boldsymbol{Tc}}\boldsymbol{=6}$*, 2 Occasions and 1,000 Observations*

| Parameter | Pop. | Estimate | *SD* | *SE* | *peb* | *seb* | 95% coverage | *MSE* | included reps | converged reps |
| --- | --- | --- | --- | --- | --- | --- | --- | --- | --- | --- |
| $\nu_{1}$ | 5.00 | 5.17 | 0.59 | 0.66 | 0.03 | **0.12** | 0.99 | 0.38 | 175 | 494 |
| $\nu_{2}$ | 5.00 | 15.86 | 93.67 | 10.54 | **2.17** | **-0.89** | 0.99 | 8892.00 | 175 | 494 |
| $\alpha_{21}$ | -1.02 | -1.06 | 0.51 | 0.56 | 0.04 | 0.08 | 0.97 | 0.27 | 175 | 494 |
| $\alpha_{22}$ | 0.70 | 0.72 | 0.83 | 1.00 | 0.03 | **0.20** | 0.931 | 0.69 | 175 | 494 |
| Logit | 1.15 | 1.12 | 0.25 | 0.38 | -0.03 | **0.52** | 0.98 | 0.06 | 175 | 494 |
| $\mu_{T1}$ | 14.32 | 14.42 | 0.41 | 0.50 | 0.01 | **0.22** | 0.95 | 0.18 | 175 | 494 |
| $\mu_{T2}$ | 17.79 | 17.90 | 0.67 | 0.96 | 0.01 | **0.43** | 0.95 | 0.46 | 175 | 494 |
| $\delta_{T1}$ | 6.00 | 5.89 | 0.60 | 0.70 | -0.02 | **0.16** | 0.98 | 0.38 | 175 | 494 |
| $\delta_{T2}$ | 6.00 | 5.70 | 0.82 | 1.06 | -0.05 | **0.30** | 0.98 | 0.75 | 175 | 494 |
| $\lambda_{21}$ | 1.05 | 1.05 | 0.03 | 0.03 | 0.00 | 0.06 | 0.95 | 0.00 | 175 | 494 |
| $\lambda_{22}$ | 0.94 | 0.94 | 0.04 | 0.04 | 0.00 | **0.18** | 0.99 | 0.00 | 175 | 494 |
| $Var(E_{11})$ | 2.30 | 2.35 | 0.22 | 0.26 | 0.02 | **0.20** | 0.97 | 0.05 | 175 | 494 |
| $Var(E_{12})$ | 1.06 | 1.05 | 0.28 | 0.31 | -0.01 | **0.12** | 0.97 | 0.08 | 175 | 494 |
| $Var(E_{21})$ | 1.42 | 1.44 | 0.15 | 0.19 | 0.02 | **0.29** | 0.96 | 0.02 | 175 | 494 |
| $Var(E_{22})$ | 0.64 | 0.64 | 0.20 | 0.24 | -0.01 | **0.14** | 0.99 | 0.04 | 175 | 494 |
| $Var({IST}_{21})$ | 0.63 | 0.60 | 0.22 | 0.24 | -0.05 | 0.08 | 0.99 | 0.05 | 175 | 494 |
| $Var({IST}_{22})$ | 0.33 | 0.38 | 0.26 | 0.31 | **0.16** | **0.18** | 0.96 | 0.07 | 175 | 494 |
| $Var(O_{1})$ | 9.97 | 10.18 | 0.93 | 1.35 | 0.02 | **0.45** | 0.96 | 0.91 | 175 | 494 |
| $Var(O_{2})$ | 0.78 | 0.81 | 0.35 | 0.42 | 0.03 | **0.18** | 0.98 | 0.12 | 175 | 494 |
| $\sigma_{T1}^{2}$ | 1.59 | 1.98 | 1.20 | 1.59 | **0.25** | **0.33** | 0.96 | 1.60 | 175 | 494 |
| $\sigma_{T2}^{2}$ | 0.64 | 2.16 | 2.38 | 3.59 | **2.38** | **0.51** | 0.97 | 7.97 | 175 | 494 |
| *Note*. Absolute *peb* and *seb* values exceeding the cut-offs for at least medium bias (> 0.10) are printed in bold. *c* = index for class; $\alpha_{2c}$= intercept for the second indicator; $\delta_{Tc}$= skewness*;*$E_{jc}$= residual variable for occasion *j*; Estimate = mean of estimates of all included replications; $IST_{2c}$ = indicator-specific trait factor*;*$\lambda_{2c}$ = trait loading*;MSE* = mean squared error;$O_{c}$= occasion-specific factor*;peb* = parameter estimation bias; Pop. = population value; reps = replications; *SD* = standard deviation of parameter estimates; *SE* = standard error; *seb* = standard error bias; *T*_c_ = latent trait variable*;* $\nu_{c}$= degrees of freedom parameter; $\mu_{Tc}$ = location parameter for the latent trait variable; $\sigma_{Tc}^{2}$ = scale parameter for the latent trait variable. | | | | | | | | | | |

Table S 8. *Results for the Simulation Study for the Skew t Mixture Latent State-Trait Model with* $\boldsymbol{\delta}_{\boldsymbol{Tc}}\boldsymbol{=6}$*, 3 Occasions and 250 Observations*

| Parameter | Pop. | Estimate | *SD* | *SE* | *peb* | *seb* | 95% coverage | *MSE* | included reps | converged reps |
| --- | --- | --- | --- | --- | --- | --- | --- | --- | --- | --- |
| $\nu_{1}$ | 5.00 | 5.53 | 1.74 | 1.22 | **0.11** | **-0.30** | 0.95 | 3.31 | 103 | 494 |
| $\nu_{2}$ | 5.00 | 108.38 | 452.66 | 126.48 | **20.68** | **-0.72** | 0.93 | 215588.89 | 103 | 494 |
| $\alpha_{21}$ | -1.02 | -1.02 | 0.68 | 0.76 | 0.00 | **0.13** | 0.97 | 0.46 | 103 | 494 |
| $\alpha_{22}$ | 0.70 | 0.72 | 1.27 | 1.41 | 0.03 | **0.11** | 0.91 | 1.61 | 103 | 494 |
| Logit | 1.15 | 1.12 | 0.31 | 0.42 | -0.03 | **0.35** | 0.95 | 0.10 | 103 | 494 |
| $\mu_{T1}$ | 14.32 | 14.58 | 0.63 | 0.78 | 0.02 | **0.24** | 0.92 | 0.46 | 103 | 494 |
| $\mu_{T2}$ | 17.79 | 18.38 | 1.31 | 1.42 | 0.03 | 0.08 | 0.95 | 2.05 | 103 | 494 |
| $\delta_{T1}$ | 6.00 | 5.83 | 0.85 | 1.05 | -0.03 | **0.24** | 0.95 | 0.75 | 103 | 494 |
| $\delta_{T2}$ | 6.00 | 5.15 | 1.66 | 1.57 | **-0.14** | -0.05 | 0.96 | 3.48 | 103 | 494 |
| $\lambda_{21}$ | 1.05 | 1.05 | 0.04 | 0.04 | 0.00 | **0.13** | 0.93 | 0.00 | 103 | 494 |
| $\lambda_{22}$ | 0.94 | 0.94 | 0.05 | 0.06 | 0.00 | **0.13** | 1.00 | 0.00 | 103 | 494 |
| $Var(E_{11})$ | 2.30 | 2.35 | 0.34 | 0.42 | 0.02 | **0.24** | 0.93 | 0.12 | 103 | 494 |
| $Var(E_{12})$ | 1.06 | 1.04 | 0.39 | 0.41 | -0.01 | 0.05 | 0.98 | 0.15 | 103 | 494 |
| $Var(E_{21})$ | 1.42 | 1.46 | 0.20 | 0.24 | 0.03 | **0.25** | 0.98 | 0.04 | 103 | 494 |
| $Var(E_{22})$ | 0.64 | 0.61 | 0.17 | 0.22 | -0.05 | **0.34** | 0.98 | 0.03 | 103 | 494 |
| $Var({IST}_{21})$ | 0.63 | 0.60 | 0.25 | 0.28 | -0.04 | **0.10** | 0.91 | 0.06 | 103 | 494 |
| $Var({IST}_{22})$ | 0.33 | 0.39 | 0.29 | 0.32 | **0.18** | 0.08 | 0.95 | 0.09 | 103 | 494 |
| $Var(O_{1})$ | 9.97 | 10.36 | 1.48 | 1.65 | 0.04 | **0.11** | 0.96 | 2.35 | 103 | 494 |
| $Var(O_{2})$ | 0.78 | 0.83 | 0.34 | 0.41 | 0.06 | **0.22** | 0.97 | 0.12 | 103 | 494 |
| $\sigma_{T1}^{2}$ | 1.59 | 2.31 | 1.52 | 2.35 | **0.45** | **0.54** | 0.96 | 2.84 | 103 | 494 |
| $\sigma_{T2}^{2}$ | 0.64 | 3.12 | 3.51 | 4.06 | **3.87** | **0.16** | 0.96 | 18.48 | 103 | 494 |
| *Note*. Absolute *peb* and *seb* values exceeding the cut-offs for at least medium bias (> 0.10) are printed in bold. *c* = index for class; $\alpha_{2c}$= intercept for the second indicator; $\delta_{Tc}$= skewness*;*$E_{jc}$= residual variable for occasion *j*; Estimate = mean of estimates of all included replications; $IST_{2c}$ = indicator-specific trait factor*;*$\lambda_{2c}$ = trait loading*;MSE* = mean squared error;$O_{c}$= occasion-specific factor*;peb* = parameter estimation bias; Pop. = population value; reps = replications; *SD* = standard deviation of parameter estimates; *SE* = standard error; *seb* = standard error bias; *T*_c_ = latent trait variable*;*$\nu_{c}$= degrees of freedom parameter; $\mu_{Tc}$ = location parameter for the latent trait variable; $\sigma_{Tc}^{2}$ = scale parameter for the latent trait variable. | | | | | | | | | | |

**Table S 9.** *Results for the Simulation Study for the Skew t Mixture Latent State-Trait Model with* $\delta_{Tc}=6$*, 3 Occasions and 500 Observations*

| Parameter | Pop. | Estimate | *SD* | *SE* | *peb* | *seb* | 95% coverage | *MSE* | included reps | converged reps |
| --- | --- | --- | --- | --- | --- | --- | --- | --- | --- | --- |
| $\nu_{1}$ | 5.00 | 5.25 | 0.68 | 0.82 | 0.05 | 0.21 | 0.99 | 0.53 | 206 | 500 |
| $\nu_{2}$ | 5.00 | 7.30 | 9.92 | 10.02 | **0.46** | 0.01 | 0.98 | 103.71 | 206 | 500 |
| $\alpha_{21}$ | -1.02 | -1.05 | 0.45 | 0.74 | 0.03 | **0.65** | 0.95 | 0.20 | 206 | 500 |
| $\alpha_{22}$ | 0.70 | 0.69 | 0.76 | 1.29 | -0.01 | **0.71** | 0.96 | 0.57 | 206 | 500 |
| Logit | 1.15 | 1.12 | 0.22 | 0.56 | -0.03 | **1.56** | 0.98 | 0.05 | 206 | 500 |
| $\mu_{T1}$ | 14.32 | 14.41 | 0.40 | 0.64 | 0.01 | **0.57** | 0.94 | 0.17 | 206 | 500 |
| $\mu_{T2}$ | 17.79 | 17.90 | 0.77 | 2.68 | 0.01 | **2.47** | 0.96 | 0.61 | 206 | 500 |
| $\delta_{T1}$ | 6.00 | 5.94 | 0.60 | 0.80 | -0.01 | **0.34** | 0.97 | 0.36 | 206 | 500 |
| $\delta_{T2}$ | 6.00 | 5.72 | 0.99 | 3.61 | -0.05 | **2.63** | 0.95 | 1.07 | 206 | 500 |
| $\lambda_{21}$ | 1.05 | 1.05 | 0.02 | 0.04 | 0.00 | **0.68** | 0.94 | 0.00 | 206 | 500 |
| $\lambda_{22}$ | 0.94 | 0.94 | 0.03 | 0.06 | 0.00 | **0.69** | 1.00 | 0.00 | 206 | 500 |
| $Var(E_{11})$ | 2.30 | 2.32 | 0.26 | 0.29 | 0.01 | 0.14 | 0.98 | 0.07 | 206 | 500 |
| $Var(E_{12})$ | 1.06 | 1.07 | 0.25 | 0.31 | 0.01 | 0.22 | 0.99 | 0.06 | 206 | 500 |
| $Var(E_{21})$ | 1.42 | 1.44 | 0.13 | 0.15 | 0.02 | 0.17 | 0.94 | 0.02 | 206 | 500 |
| $Var(E_{22})$ | 0.64 | 0.63 | 0.13 | 0.19 | -0.01 | **0.49** | 0.98 | 0.02 | 206 | 500 |
| $Var({IST}_{21})$ | 0.63 | 0.62 | 0.18 | 0.23 | -0.01 | 0.26 | 0.98 | 0.03 | 206 | 500 |
| $Var({IST}_{22})$ | 0.33 | 0.33 | 0.18 | 0.28 | -0.01 | **0.59** | 0.98 | 0.03 | 206 | 500 |
| $Var(O_{1})$ | 9.97 | 10.21 | 0.99 | 1.65 | 0.02 | **0.66** | 0.98 | 1.05 | 206 | 500 |
| $Var(O_{2})$ | 0.78 | 0.81 | 0.26 | 0.51 | 0.04 | **0.96** | 0.97 | 0.07 | 206 | 500 |
| $\sigma_{T1}^{2}$ | 1.59 | 1.77 | 1.19 | 3.03 | **0.11** | **1.54** | 0.96 | 1.45 | 206 | 500 |
| $\sigma_{T2}^{2}$ | 0.64 | 2.14 | 2.51 | 12.15 | **2.34** | **3.84** | 0.98 | 8.55 | 206 | 500 |
| *Note*. Absolute *peb* and *seb* values exceeding the cut-offs for at least medium bias (> 0.10) are printed in bold*. c =* index for class; $\alpha_{2c}$= intercept for the second indicator; $\delta_{Tc}$= skewness*;*$E_{jc}$= residual variable for occasion *j*; Estimate = mean of estimates of all included replications; $IST_{2c}$ = indicator-specific trait factor*;*$\lambda_{2c}$ = trait loading*;MSE* = mean squared error;$O_{c}$= occasion-specific factor*;peb* = parameter estimation bias; Pop. = population value; reps = replications; *SD* = standard deviation of parameter estimates; *SE* = standard error; *seb* = standard error bias; *T*_c_ = latent trait variable*;* $\nu_{c}$= degrees of freedom parameter; $\mu_{Tc}$ = location parameter for the latent trait variable; $\sigma_{Tc}^{2}$ = scale parameter for the latent trait variable. | | | | | | | | | | |

Table S 10. *Results for the Simulation Study for the Skew t Mixture Latent State-Trait Model with* $\boldsymbol{\delta}_{\boldsymbol{Tc}}\boldsymbol{=6}$*, 3 Occasions and 1,000 Observations*

| Parameter | Pop. | Estimate | *SD* | *SE* | *peb* | *seb* | 95% coverage | *MSE* | included reps | converged reps |
| --- | --- | --- | --- | --- | --- | --- | --- | --- | --- | --- |
| $\nu_{1}$ | 5.00 | 5.14 | 0.45 | 0.48 | 0.03 | 0.07 | 0.98 | 0.22 | 309 | 499 |
| $\nu_{2}$ | 5.00 | 5.53 | 4.91 | 2.22 | **0.11** | **-0.55** | 0.96 | 24.40 | 309 | 499 |
| $\alpha_{21}$ | -1.02 | -1.06 | 0.31 | 0.34 | 0.03 | 0.09 | 0.95 | 0.10 | 309 | 499 |
| $\alpha_{22}$ | 0.70 | 0.67 | 0.51 | 0.56 | -0.04 | **0.10** | 0.95 | 0.26 | 309 | 499 |
| Logit | 1.15 | 1.14 | 0.18 | 0.19 | -0.01 | 0.08 | 0.97 | 0.03 | 309 | 499 |
| $\mu_{T1}$ | 14.32 | 14.38 | 0.31 | 0.35 | 0.00 | **0.15** | 0.95 | 0.10 | 309 | 499 |
| $\mu_{T2}$ | 17.79 | 17.90 | 0.45 | 0.60 | 0.01 | **0.35** | 0.95 | 0.21 | 309 | 499 |
| $\delta_{T1}$ | 6.00 | 5.95 | 0.44 | 0.49 | -0.01 | **0.13** | 0.95 | 0.19 | 309 | 499 |
| $\delta_{T2}$ | 6.00 | 5.84 | 0.55 | 0.74 | -0.03 | **0.35** | 0.97 | 0.33 | 309 | 499 |
| $\lambda_{21}$ | 1.05 | 1.05 | 0.02 | 0.02 | 0.00 | 0.09 | 0.92 | 0.00 | 309 | 499 |
| $\lambda_{22}$ | 0.94 | 0.94 | 0.02 | 0.02 | 0.00 | **0.10** | 0.99 | 0.00 | 309 | 499 |
| $Var(E_{11})$ | 2.30 | 2.31 | 0.19 | 0.19 | 0.01 | 0.00 | 0.96 | 0.04 | 309 | 499 |
| $Var(E_{12})$ | 1.06 | 1.08 | 0.19 | 0.20 | 0.02 | 0.05 | 0.97 | 0.04 | 309 | 499 |
| $Var(E_{21})$ | 1.42 | 1.43 | 0.09 | 0.10 | 0.01 | **0.12** | 0.92 | 0.01 | 309 | 499 |
| $Var(E_{22})$ | 0.64 | 0.65 | 0.09 | 0.10 | 0.01 | **0.12** | 0.97 | 0.01 | 309 | 499 |
| $Var({IST}_{21})$ | 0.63 | 0.63 | 0.13 | 0.13 | 0.00 | -0.01 | 0.98 | 0.02 | 309 | 499 |
| $Var({IST}_{22})$ | 0.33 | 0.33 | 0.13 | 0.14 | -0.01 | 0.04 | 0.96 | 0.02 | 309 | 499 |
| $Var(O_{1})$ | 9.97 | 10.10 | 0.67 | 0.75 | 0.01 | **0.12** | 0.96 | 0.46 | 309 | 499 |
| $Var(O_{2})$ | 0.78 | 0.78 | 0.19 | 0.19 | 0.00 | 0.02 | 0.96 | 0.03 | 309 | 499 |
| $\sigma_{T1}^{2}$ | 1.59 | 1.72 | 0.92 | 1.07 | 0.08 | **0.17** | 0.97 | 0.86 | 309 | 499 |
| $\sigma_{T2}^{2}$ | 0.64 | 1.63 | 1.77 | 2.30 | **1.54** | **0.30** | 0.96 | 4.10 | 309 | 499 |
| *Note*. Absolute *peb* and *seb* values exceeding the cut-offs for at least medium bias (> 0.10) are printed in bold*. c =* index for class; $\alpha_{2c}$= intercept for the second indicator; $\delta_{Tc}$= skewness*; E_jc_* = residual variable at occasion *j*; Estimate = mean of estimates of all included replications; $IST_{2c}$ = indicator-specific trait factor*;*$\lambda_{2c}$ = trait loading*;MSE* = mean squared error;$O_{c}$= occasion-specific factor*;peb* = parameter estimation bias; Pop. = population value; reps = replications; *SD* = standard deviation of parameter estimates; *SE* = standard error; *seb* = standard error bias; *T*_c_ = latent trait variable*;*$\nu_{c}$= degrees of freedom parameter; $\mu_{Tc}$ = location parameter for the latent trait variable; $\sigma_{Tc}^{2}$ = scale parameter for the latent trait variable. | | | | | | | | | | |

Table S 11. *Results for the Simulation Study for the Skew t Mixture Latent State-Trait Model with* $\boldsymbol{\delta}_{\boldsymbol{Tc}}\boldsymbol{=6}$*, 4 Occasions and 125 Observations*

| Parameter | Pop. | Estimate | *SD* | *SE* | *peb* | *seb* | 95% coverage | *MSE* | included reps | converged reps |
| --- | --- | --- | --- | --- | --- | --- | --- | --- | --- | --- |
| $\nu_{1}$ | 5.00 | 5.45 | 1.48 | 1.41 | 0.09 | -0.05 | 0.96 | 2.40 | 84 | 496 |
| $\nu_{2}$ | 5.00 | 130.78 | 434.60 | 293.14 | **25.16** | **-0.33** | 0.92 | 204695.10 | 84 | 496 |
| $\alpha_{21}$ | -1.02 | -1.02 | 0.93 | 0.95 | 0.00 | 0.02 | 0.92 | 0.87 | 84 | 496 |
| $\alpha_{22}$ | 0.70 | 0.79 | 1.33 | 1.69 | **0.13** | **0.28** | 0.93 | 1.77 | 84 | 496 |
| Logit | 1.15 | 1.09 | 0.33 | 0.49 | -0.06 | **0.52** | 0.95 | 0.11 | 84 | 496 |
| $\mu_{T1}$ | 14.32 | 14.69 | 0.77 | 1.05 | 0.03 | **0.36** | 0.92 | 0.73 | 84 | 496 |
| $\mu_{T2}$ | 17.79 | 18.68 | 2.13 | 2.08 | 0.05 | -0.03 | 0.90 | 5.34 | 84 | 496 |
| $\delta_{T1}$ | 6.00 | 5.69 | 1.04 | 1.40 | -0.05 | **0.34** | 0.96 | 1.18 | 84 | 496 |
| $\delta_{T2}$ | 6.00 | 4.88 | 2.32 | 2.21 | -**0.19** | -0.05 | 0.99 | 6.66 | 84 | 496 |
| $\lambda_{21}$ | 1.05 | 1.05 | 0.05 | 0.05 | 0.00 | 0.04 | 0.96 | 0.00 | 84 | 496 |
| $\lambda_{22}$ | 0.94 | 0.94 | 0.06 | 0.07 | -0.01 | **0.27** | 0.99 | 0.00 | 84 | 496 |
| $Var(E_{11})$ | 2.30 | 2.25 | 0.48 | 0.55 | -0.02 | **0.15** | 0.92 | 0.23 | 84 | 496 |
| $Var(E_{12})$ | 1.06 | 1.17 | 0.49 | 0.61 | **0.11** | **0.24** | 0.95 | 0.25 | 84 | 496 |
| $Var(E_{21})$ | 1.42 | 1.44 | 0.23 | 0.25 | 0.02 | 0.09 | 0.98 | 0.05 | 84 | 496 |
| $Var(E_{22})$ | 0.64 | 0.68 | 0.26 | 0.24 | 0.05 | -0.05 | 0.98 | 0.07 | 84 | 496 |
| $Var({IST}_{21})$ | 0.63 | 0.62 | 0.27 | 0.32 | -0.01 | **0.16** | 0.93 | 0.08 | 84 | 496 |
| $Var({IST}_{22})$ | 0.33 | 0.37 | 0.30 | 0.35 | **0.11** | **0.17** | 0.92 | 0.09 | 84 | 496 |
| $Var(O_{1})$ | 9.97 | 10.35 | 1.59 | 2.05 | 0.04 | **0.29** | 0.88 | 2.68 | 84 | 496 |
| $Var(O_{2})$ | 0.78 | 0.81 | 0.30 | 0.50 | 0.04 | **0.66** | 0.93 | 0.09 | 84 | 496 |
| $\sigma_{T1}^{2}$ | 1.59 | 2.82 | 2.20 | 3.17 | **0.77** | **0.45** | 0.94 | 6.33 | 84 | 496 |
| $\sigma_{T2}^{2}$ | 0.64 | 4.53 | 5.00 | 4.74 | **6.09** | -0.05 | 0.89 | 40.17 | 84 | 496 |
| *Note*. Absolute *peb* and *seb* values exceeding the cut-offs for at least medium bias (> 0.10) are printed in bold*. c =* index for class; $\alpha_{2c}$= intercept for the second indicator; $\delta_{Tc}$= skewness*; E_jc_* = residual variable at occasion *j*; Estimate = mean of estimates of all included replications; $IST_{2c}$ = indicator-specific trait factor*;*$\lambda_{2c}$ = trait loading*;MSE* = mean squared error;$O_{c}$= occasion-specific factor*;peb* = parameter estimation bias; Pop. = population value; reps = replications; *SD* = standard deviation of parameter estimates; *SE* = standard error; *seb* = standard error bias; *T*_c_ = latent trait variable*;*$\nu_{c}$= degrees of freedom parameter*;* $\mu_{Tc}$ = location parameter for the latent trait variable; $\sigma_{Tc}^{2}$ = scale parameter for the latent trait variable. | | | | | | | | | | |

Table S 12. *Results for the Simulation Study for the Skew t Mixture Latent State-Trait Model with* $\boldsymbol{\delta}_{\boldsymbol{Tc}}\boldsymbol{=6}$*, 4 Occasions and 250 Observations*

| Parameter | Pop. | Estimate | *SD* | *SE* | *peb* | *seb* | 95% coverage | *MSE* | included reps | converged reps |  |
| --- | --- | --- | --- | --- | --- | --- | --- | --- | --- | --- | --- |
| $\nu_{1}$ | 5.00 | 5.20 | 0.93 | 0.90 | 0.04 | -0.03 | 0.96 | 0.90 | 188 | 498 |  |
| $\nu_{2}$ | 5.00 | 7.27 | 5.75 | 7.75 | **0.45** | **0.35** | 0.98 | 38.23 | 188 | 498 |  |
| $\alpha_{21}$ | -1.02 | -1.00 | 0.56 | 0.71 | -0.02 | **0.26** | 0.95 | 0.32 | 188 | 498 |  |
| $\alpha_{22}$ | 0.70 | 0.77 | 0.89 | 1.01 | 0.10 | **0.13** | 0.91 | 0.79 | 188 | 498 |  |
| Logit | 1.15 | 1.13 | 0.25 | 0.35 | -0.02 | **0.39** | 0.94 | 0.06 | 188 | 498 |  |
| $\mu_{T1}$ | 14.32 | 14.47 | 0.56 | 0.65 | 0.01 | **0.16** | 0.95 | 0.33 | 188 | 498 |  |
| $\mu_{T2}$ | 17.79 | 18.20 | 1.03 | 1.33 | 0.02 | **0.29** | 0.95 | 1.22 | 188 | 498 |  |
| $\delta_{T1}$ | 6.00 | 5.83 | 0.83 | 0.94 | -0.03 | **0.13** | 0.95 | 0.72 | 188 | 498 |  |
| $\delta_{T2}$ | 6.00 | 5.52 | 1.19 | 1.63 | -0.08 | **0.37** | 0.95 | 1.65 | 188 | 498 |  |
| $\lambda_{21}$ | 1.05 | 1.05 | 0.03 | 0.04 | 0.00 | **0.26** | 0.95 | 0.00 | 188 | 498 |  |
| $\lambda_{22}$ | 0.94 | 0.94 | 0.04 | 0.04 | 0.00 | **0.10** | 1.00 | 0.00 | 188 | 498 |  |
| $Var(E_{11})$ | 2.30 | 2.29 | 0.33 | 0.37 | 0.00 | **0.12** | 0.98 | 0.11 | 188 | 498 |  |
| $Var(E_{12})$ | 1.06 | 1.09 | 0.35 | 0.38 | 0.03 | 0.09 | 0.96 | 0.12 | 188 | 498 |  |
| $Var(E_{21})$ | 1.42 | 1.44 | 0.16 | 0.17 | 0.01 | 0.08 | 0.95 | 0.03 | 188 | 498 |  |
| $Var(E_{22})$ | 0.64 | 0.64 | 0.13 | 0.16 | 0.01 | **0.24** | 0.94 | 0.02 | 188 | 498 |  |
| $Var({IST}_{21})$ | 0.63 | 0.62 | 0.20 | 0.22 | -0.02 | **0.11** | 0.96 | 0.04 | 188 | 498 |  |
| $Var({IST}_{22})$ | 0.33 | 0.33 | 0.18 | 0.24 | 0.01 | **0.36** | 0.95 | 0.03 | 188 | 498 |  |
| $Var(O_{1})$ | 9.97 | 10.10 | 1.16 | 1.27 | 0.01 | **0.10** | 0.95 | 1.36 | 188 | 498 |  |
| $Var(O_{2})$ | 0.78 | 0.78 | 0.24 | 0.30 | 0.00 | **0.23** | 0.96 | 0.06 | 188 | 498 |  |
| $\sigma_{T1}^{2}$ | 1.59 | 2.16 | 1.45 | 1.83 | **0.36** | **0.26** | 0.96 | 2.43 | 188 | 498 |  |
| $\sigma_{T2}^{2}$ | 0.64 | 2.67 | 2.91 | 3.66 | **3.17** | **0.26** | 0.94 | 12.59 | 188 | 498 |  |
| *Note*. Absolute *peb* and *seb* values exceeding the cut-offs for at least medium bias (> 0.10) are printed in bold*. c =* index for class; $\alpha_{2c}$= intercept for the second indicator; $\delta_{Tc}$= skewness*; E_jc_* = residual variable at occasion *j*; Estimate = mean of estimates of all included replications; $IST_{2c}$ = indicator-specific trait factor*;*$\lambda_{2c}$ = trait loading*;MSE* = mean squared error;$O_{c}$= occasion-specific factor*;peb* = parameter estimation bias; Pop. = population value; reps = replications; *SD* = standard deviation of parameter estimates; *SE* = standard error; *seb* = standard error bias; *T*_c_ = latent trait variable*;*$\nu_{c}$= degrees of freedom parameter; $\mu_{Tc}$ = location parameter for the latent trait variable; $\sigma_{Tc}^{2}$ = scale parameter for the latent trait variable. | | | | | | | | | | | |

Table S 13. *Results for the Simulation Study for the Skew t Mixture Latent State-Trait Model with* $\boldsymbol{\delta}_{\boldsymbol{Tc}}\boldsymbol{=6}$*, 4 Occasions and 500 Observations*

| Parameter | Pop. | Estimate | *SD* | *SE* | *peb* | *seb* | 95% coverage | *MSE* | included reps | converged reps |  |
| --- | --- | --- | --- | --- | --- | --- | --- | --- | --- | --- | --- |
| $\nu_{1}$ | 5.00 | 5.18 | 0.62 | 0.88 | 0.04 | **0.41** | 0.98 | 0.42 | 281 | 498 |  |
| $\nu_{2}$ | 5.00 | 5.68 | 2.37 | 2.69 | **0.14** | **0.14** | 0.97 | 6.10 | 281 | 498 |  |
| $\alpha_{21}$ | -1.02 | -1.01 | 0.34 | 0.51 | 0.00 | **0.48** | 0.95 | 0.12 | 281 | 498 |  |
| $\alpha_{22}$ | 0.70 | 0.70 | 0.58 | 1.30 | 0.01 | **1.23** | 0.94 | 0.34 | 281 | 498 |  |
| Logit | 1.15 | 1.13 | 0.17 | 0.51 | -0.02 | **2.01** | 0.97 | 0.03 | 281 | 498 |  |
| $\mu_{T1}$ | 14.32 | 14.36 | 0.35 | 0.47 | 0.00 | **0.36** | 0.94 | 0.12 | 281 | 498 |  |
| $\mu_{T2}$ | 17.79 | 17.99 | 0.60 | 1.67 | 0.01 | **1.77** | 0.95 | 0.40 | 281 | 498 |  |
| $\delta_{T1}$ | 6.00 | 5.98 | 0.54 | 0.64 | 0.00 | **0.19** | 0.95 | 0.29 | 281 | 498 |  |
| $\delta_{T2}$ | 6.00 | 5.70 | 0.74 | 1.09 | -0.05 | **0.47** | 0.95 | 0.64 | 281 | 498 |  |
| $\lambda_{21}$ | 1.05 | 1.05 | 0.02 | 0.02 | 0.00 | **0.29** | 0.98 | 0.00 | 281 | 498 |  |
| $\lambda_{22}$ | 0.94 | 0.94 | 0.03 | 0.05 | 0.00 | **1.12** | 1.00 | 0.00 | 281 | 498 |  |
| $Var(E_{11})$ | 2.30 | 2.32 | 0.24 | 0.32 | 0.01 | **0.31** | 0.98 | 0.06 | 281 | 498 |  |
| $Var(E_{12})$ | 1.06 | 1.08 | 0.23 | 0.37 | 0.02 | **0.59** | 0.95 | 0.05 | 281 | 498 |  |
| $Var(E_{21})$ | 1.42 | 1.43 | 0.10 | 0.21 | 0.01 | **1.08** | 0.95 | 0.01 | 281 | 498 |  |
| $Var(E_{22})$ | 0.64 | 0.64 | 0.10 | 0.12 | 0.00 | **0.22** | 0.97 | 0.01 | 281 | 498 |  |
| $Var({IST}_{21})$ | 0.63 | 0.63 | 0.13 | 0.18 | 0.00 | **0.38** | 0.98 | 0.02 | 281 | 498 |  |
| $Var({IST}_{22})$ | 0.33 | 0.33 | 0.12 | 0.23 | 0.00 | **0.89** | 0.98 | 0.02 | 281 | 498 |  |
| $Var(O_{1})$ | 9.97 | 10.10 | 0.77 | 1.66 | 0.01 | **1.17** | 0.95 | 0.60 | 281 | 498 |  |
| $Var(O_{2})$ | 0.78 | 0.80 | 0.16 | 0.43 | 0.02 | **1.63** | 0.98 | 0.03 | 281 | 498 |  |
| $\sigma_{T1}^{2}$ | 1.59 | 1.74 | 1.00 | 1.39 | **0.10** | **0.38** | 0.98 | 1.03 | 281 | 498 |  |
| $\sigma_{T2}^{2}$ | 0.64 | 1.80 | 2.01 | 4.06 | **1.81** | **1.02** | 0.95 | 5.39 | 281 | 498 |  |
| *Note*. Absolute *peb* and *seb* values exceeding the cut-offs for at least medium bias (> 0.10) are printed in bold*. c =* index for class; $\alpha_{2c}$= intercept for the second indicator; $\delta_{Tc}$= skewness*; E_jc_* = residual variable at occasion *j*; Estimate = mean of estimates of all included replications; $IST_{2c}$ = indicator-specific trait factor*;*$\lambda_{2c}$ = trait loading*;MSE* = mean squared error;$O_{c}$= occasion-specific factor*;peb* = parameter estimation bias; Pop. = population value; reps = replications; *SD* = standard deviation of parameter estimates; *SE* = standard error; *seb* = standard error bias; *T*_c_ = latent trait variable*;*$\nu_{c}$= degrees of freedom parameter; $\mu_{Tc}$ = location parameter for the latent trait variable; $\sigma_{Tc}^{2}$ = scale parameter for the latent trait variable. | | | | | | | | | | | |

Table S 14. *Results for the Simulation Study for the Skew t Mixture Latent State-Trait Model with* $\boldsymbol{\delta}_{\boldsymbol{Tc}}\boldsymbol{=6}$*, 4 Occasions and 1,000 Observations*

| Parameter | Pop. | Estimate | *SD* | *SE* | *peb* | *seb* | 95% coverage | *MSE* | included reps | converged reps |  |
| --- | --- | --- | --- | --- | --- | --- | --- | --- | --- | --- | --- |
| $\nu_{1}$ | 5.00 | 5.09 | 0.41 | 0.42 | 0.02 | 0.03 | 0.98 | 0.18 | 375 | 500 |  |
| $\nu_{2}$ | 5.00 | 5.28 | 1.33 | 1.27 | 0.06 | -0.04 | 0.98 | 1.85 | 375 | 500 |  |
| $\alpha_{21}$ | -1.02 | -1.02 | 0.28 | 0.28 | 0.00 | 0.01 | 0.96 | 0.08 | 375 | 500 |  |
| $\alpha_{22}$ | 0.70 | 0.70 | 0.39 | 0.42 | 0.00 | 0.06 | 0.94 | 0.16 | 375 | 500 |  |
| Logit | 1.15 | 1.13 | 0.12 | 0.15 | -0.02 | **0.21** | 0.94 | 0.02 | 375 | 500 |  |
| $\mu_{T1}$ | 14.32 | 14.32 | 0.26 | 0.28 | 0.00 | 0.08 | 0.96 | 0.07 | 375 | 500 |  |
| $\mu_{T2}$ | 17.79 | 17.88 | 0.33 | 0.43 | 0.00 | **0.30** | 0.95 | 0.12 | 375 | 500 |  |
| $\delta_{T1}$ | 6.00 | 6.00 | 0.39 | 0.40 | 0.00 | 0.01 | 0.94 | 0.15 | 375 | 500 |  |
| $\delta_{T2}$ | 6.00 | 5.87 | 0.46 | 0.60 | -0.02 | **0.29** | 0.96 | 0.23 | 375 | 500 |  |
| $\lambda_{21}$ | 1.05 | 1.05 | 0.01 | 0.02 | 0.00 | 0.01 | 0.97 | 0.00 | 375 | 500 |  |
| $\lambda_{22}$ | 0.94 | 0.94 | 0.02 | 0.02 | 0.00 | 0.06 | 0.95 | 0.00 | 375 | 500 |  |
| $Var(E_{11})$ | 2.30 | 2.31 | 0.17 | 0.18 | 0.00 | 0.03 | 0.97 | 0.03 | 375 | 500 |  |
| $Var(E_{12})$ | 1.06 | 1.07 | 0.15 | 0.16 | 0.00 | 0.02 | 0.95 | 0.02 | 375 | 500 |  |
| $Var(E_{21})$ | 1.42 | 1.43 | 0.08 | 0.08 | 0.01 | 0.04 | 0.93 | 0.01 | 375 | 500 |  |
| $Var(E_{22})$ | 0.64 | 0.64 | 0.07 | 0.07 | 0.01 | 0.04 | 0.96 | 0.00 | 375 | 500 |  |
| $Var({IST}_{21})$ | 0.63 | 0.63 | 0.09 | 0.10 | 0.00 | 0.05 | 0.97 | 0.01 | 375 | 500 |  |
| $Var({IST}_{22})$ | 0.33 | 0.33 | 0.09 | 0.09 | -0.01 | 0.02 | 0.94 | 0.01 | 375 | 500 |  |
| $Var(O_{1})$ | 9.97 | 10.06 | 0.55 | 0.61 | 0.01 | **0.11** | 0.95 | 0.31 | 375 | 500 |  |
| $Var(O_{2})$ | 0.78 | 0.80 | 0.13 | 0.14 | 0.03 | 0.08 | 0.96 | 0.02 | 375 | 500 |  |
| $\sigma_{T1}^{2}$ | 1.59 | 1.63 | 0.81 | 0.84 | 0.02 | 0.04 | 0.95 | 0.66 | 375 | 500 |  |
| $\sigma_{T2}^{2}$ | 0.64 | 1.23 | 1.39 | 1.60 | **0.92** | **0.15** | 0.94 | 2.29 | 375 | 500 |  |
| *Note*. Absolute *peb* and *seb* values exceeding the cut-offs for at least medium bias (> 0.10) are printed in bold*. c =* index for class; $\alpha_{2c}$= intercept for the second indicator; $\delta_{Tc}$= skewness*; E_jc_* = residual variable at occasion *j*; Estimate = mean of estimates of all included replications; $IST_{2c}$ = indicator-specific trait factor*;*$\lambda_{2c}$ = trait loading*;MSE* = mean squared error;$O_{c}$= occasion-specific factor*;peb* = parameter estimation bias; Pop. = population value; reps = replications; *SD* = standard deviation of parameter estimates; *SE* = standard error; *seb* = standard error bias; *T*_c_ = latent trait variable*;*$\nu_{c}$= degrees of freedom parameter; $\mu_{Tc}$ = location parameter for the latent trait variable; $\sigma_{Tc}^{2}$ = scale parameter for the latent trait variable. | | | | | | | | | | | |

Table S 15. *Results for the Simulation Study for the Skew t Mixture Latent State-Trait Model with* $\boldsymbol{\delta}_{\boldsymbol{Tc}}\boldsymbol{=6}$*, 5 Occasions and 125 Observations*

| Parameter | Pop. | Estimate | *SD* | *SE* | *peb* | *seb* | 95% coverage | *MSE* | included reps | converged reps |  |
| --- | --- | --- | --- | --- | --- | --- | --- | --- | --- | --- | --- |
| $\nu_{1}$ | 5.00 | 5.51 | 1.30 | 1.26 | 0.10 | -0.03 | 0.99 | 1.94 | 127 | 492 |  |
| $\nu_{2}$ | 5.00 | 33.58 | 239.92 | 61.40 | **5.72** | **-0.74** | 0.91 | 58377.82 | 127 | 492 |  |
| $\alpha_{21}$ | -1.02 | -1.04 | 0.65 | 0.75 | 0.02 | **0.16** | 0.91 | 0.42 | 127 | 492 |  |
| $\alpha_{22}$ | 0.70 | 0.71 | 1.11 | 1.26 | 0.01 | **0.13** | 0.91 | 1.24 | 127 | 492 |  |
| Logit | 1.15 | 1.13 | 0.31 | 0.37 | -0.02 | **0.22** | 0.93 | 0.09 | 127 | 492 |  |
| $\mu_{T1}$ | 14.32 | 14.67 | 0.70 | 0.91 | 0.02 | **0.30** | 0.91 | 0.61 | 127 | 492 |  |
| $\mu_{T2}$ | 17.79 | 18.85 | 2.11 | 1.70 | 0.06 | **-0.19** | 0.90 | 5.56 | 127 | 492 |  |
| $\delta_{T1}$ | 6.00 | 5.65 | 0.93 | 1.18 | -0.06 | **0.27** | 0.91 | 0.98 | 127 | 492 |  |
| $\delta_{T2}$ | 6.00 | 4.66 | 2.53 | 2.00 | -0.22 | **-0.21** | 0.95 | 8.18 | 127 | 492 |  |
| $\lambda_{21}$ | 1.05 | 1.05 | 0.03 | 0.04 | 0.00 | **0.15** | 0.94 | 0.00 | 127 | 492 |  |
| $\lambda_{22}$ | 0.94 | 0.94 | 0.05 | 0.05 | 0.00 | 0.09 | 0.98 | 0.00 | 127 | 492 |  |
| $Var(E_{11})$ | 2.30 | 2.36 | 0.53 | 0.49 | 0.03 | -0.07 | 0.91 | 0.28 | 127 | 492 |  |
| $Var(E_{12})$ | 1.06 | 1.08 | 0.48 | 0.44 | 0.02 | -0.10 | 0.95 | 0.24 | 127 | 492 |  |
| $Var(E_{21})$ | 1.42 | 1.45 | 0.20 | 0.21 | 0.02 | 0.05 | 0.98 | 0.04 | 127 | 492 |  |
| $Var(E_{22})$ | 0.64 | 0.63 | 0.17 | 0.17 | -0.02 | -0.01 | 0.99 | 0.03 | 127 | 492 |  |
| $Var({IST}_{21})$ | 0.63 | 0.61 | 0.27 | 0.24 | -0.03 | **-0.11** | 0.86 | 0.07 | 127 | 492 |  |
| $Var({IST}_{22})$ | 0.33 | 0.33 | 0.25 | 0.23 | 0.01 | -0.04 | 0.98 | 0.06 | 127 | 492 |  |
| $Var(O_{1})$ | 9.97 | 10.21 | 1.58 | 1.58 | 0.02 | 0.00 | 0.93 | 2.55 | 127 | 492 |  |
| $Var(O_{2})$ | 0.78 | 0.79 | 0.26 | 0.34 | 0.02 | **0.29** | 0.94 | 0.07 | 127 | 492 |  |
| $\sigma_{T1}^{2}$ | 1.59 | 2.49 | 2.06 | 2.62 | **0.57** | **0.27** | 0.98 | 5.07 | 127 | 492 |  |
| $\sigma_{T2}^{2}$ | 0.64 | 3.58 | 4.67 | 3.87 | **4.60** | **-0.17** | 0.91 | 30.44 | 127 | 492 |  |
| *Note*. Absolute *peb* and *seb* values exceeding the cut-offs for at least medium bias (> 0.10) are printed in bold*. c =* index for class; $\alpha_{2c}$= intercept for the second indicator; $\delta_{Tc}$= skewness*; E_jc_* = residual variable at occasion *j*; Estimate = mean of estimates of all included replications; $IST_{2c}$ = indicator-specific trait factor*;*$\lambda_{2c}$ = trait loading*;MSE* = mean squared error;$O_{c}$= occasion-specific factor*;peb* = parameter estimation bias; Pop. = population value; reps = replications; *SD* = standard deviation of parameter estimates; *SE* = standard error; *seb* = standard error bias; *T*_c_ = latent trait variable*;*$\nu_{c}$= degrees of freedom parameter; $\mu_{Tc}$ = location parameter for the latent trait variable; $\sigma_{Tc}^{2}$ = scale parameter for the latent trait variable. | | | | | | | | | | | |

Table S 16. *Results for the Simulation Study for the Skew t Mixture Latent State-Trait Model with* $\boldsymbol{\delta}_{\boldsymbol{Tc}}\boldsymbol{=6}$*, 5 Occasions and 250 Observations*

| Parameter | Pop. | Estimate | *SD* | *SE* | *peb* | *seb* | 95% coverage | *MSE* | included reps | converged reps |  |
| --- | --- | --- | --- | --- | --- | --- | --- | --- | --- | --- | --- |
| $\nu_{1}$ | 5.00 | 5.24 | 0.83 | 0.80 | 0.05 | -0.03 | 0.95 | 0.74 | 222 | 497 |  |
| $\nu_{2}$ | 5.00 | 6.47 | 5.70 | 3.96 | **0.29** | **-0.30** | 0.92 | 34.59 | 222 | 497 |  |
| $\alpha_{21}$ | -1.02 | -1.08 | 0.54 | 0.52 | 0.06 | -0.03 | 0.95 | 0.30 | 222 | 497 |  |
| $\alpha_{22}$ | 0.70 | 0.75 | 0.75 | 0.80 | 0.07 | 0.08 | 0.93 | 0.56 | 222 | 497 |  |
| Logit | 1.15 | 1.12 | 0.23 | 0.25 | -0.03 | **0.10** | 0.95 | 0.05 | 222 | 497 |  |
| $\mu_{T1}$ | 14.32 | 14.47 | 0.52 | 0.58 | 0.01 | **0.12** | 0.95 | 0.30 | 222 | 497 |  |
| $\mu_{T2}$ | 17.79 | 18.18 | 0.88 | 0.98 | 0.02 | **0.11** | 0.91 | 0.93 | 222 | 497 |  |
| $\delta_{T1}$ | 6.00 | 5.81 | 0.70 | 0.79 | -0.03 | **0.12** | 0.94 | 0.53 | 222 | 497 |  |
| $\delta_{T2}$ | 6.00 | 5.56 | 1.08 | 1.23 | -0.07 | **0.14** | 0.94 | 1.36 | 222 | 497 |  |
| $\lambda_{21}$ | 1.05 | 1.05 | 0.03 | 0.03 | 0.00 | -0.05 | 0.94 | 0.00 | 222 | 497 |  |
| $\lambda_{22}$ | 0.94 | 0.94 | 0.03 | 0.03 | 0.00 | 0.06 | 1.00 | 0.00 | 222 | 497 |  |
| $Var(E_{11})$ | 2.30 | 2.32 | 0.34 | 0.33 | 0.01 | -0.03 | 0.95 | 0.12 | 222 | 497 |  |
| $Var(E_{12})$ | 1.06 | 1.07 | 0.30 | 0.30 | 0.01 | -0.02 | 0.95 | 0.09 | 222 | 497 |  |
| $Var(E_{21})$ | 1.42 | 1.44 | 0.15 | 0.13 | 0.01 | -0.07 | 0.95 | 0.02 | 222 | 497 |  |
| $Var(E_{22})$ | 0.64 | 0.64 | 0.11 | 0.11 | 0.00 | 0.03 | 0.96 | 0.01 | 222 | 497 |  |
| $Var({IST}_{21})$ | 0.63 | 0.61 | 0.18 | 0.17 | -0.03 | -0.08 | 0.95 | 0.03 | 222 | 497 |  |
| $Var({IST}_{22})$ | 0.33 | 0.32 | 0.16 | 0.16 | -0.03 | 0.02 | 0.95 | 0.03 | 222 | 497 |  |
| $Var(O_{1})$ | 9.97 | 10.18 | 1.00 | 1.04 | 0.02 | 0.04 | 0.94 | 1.04 | 222 | 497 |  |
| $Var(O_{2})$ | 0.78 | 0.80 | 0.21 | 0.22 | 0.03 | 0.07 | 0.94 | 0.04 | 222 | 497 |  |
| $\sigma_{T1}^{2}$ | 1.59 | 2.00 | 1.37 | 1.71 | **0.26** | **0.25** | 0.94 | 2.05 | 222 | 497 |  |
| $\sigma_{T2}^{2}$ | 0.64 | 2.24 | 2.58 | 2.69 | **2.50** | 0.04 | 0.94 | 9.21 | 222 | 497 |  |
| *Note*. Absolute *peb* and *seb* values exceeding the cut-offs for at least medium bias (> 0.10) are printed in bold*. c =* index for class; $\alpha_{2c}$= intercept for the second indicator; $\delta_{Tc}$= skewness*; E_jc_* = residual variable at occasion *j*; Estimate = mean of estimates of all included replications; $IST_{2c}$ = indicator-specific trait factor*;*$\lambda_{2c}$ = trait loading*;MSE* = mean squared error;$O_{c}$= occasion-specific factor*;peb* = parameter estimation bias; Pop. = population value; reps = replications; *SD* = standard deviation of parameter estimates; *SE* = standard error; *seb* = standard error bias; *T*_c_ = latent trait variable*;*$\nu_{c}$= degrees of freedom parameter; $\mu_{Tc}$ = location parameter for the latent trait variable; $\sigma_{Tc}^{2}$ = scale parameter for the latent trait variable. | | | | | | | | | | | |

Table S 17. *Results for the Simulation Study for the Skew t Mixture Latent State-Trait Model with* $\boldsymbol{\delta}_{\boldsymbol{Tc}}\boldsymbol{=6}$*, 5 Occasions and 500 Observations*

| Parameter | Pop. | Estimate | *SD* | *SE* | *peb* | *seb* | 95% coverage | *MSE* | included reps | converged reps |  |
| --- | --- | --- | --- | --- | --- | --- | --- | --- | --- | --- | --- |
| $\nu_{1}$ | 5.00 | 5.13 | 0.54 | 0.59 | 0.03 | 0.09 | 0.97 | 0.31 | 319 | 499 |  |
| $\nu_{2}$ | 5.00 | 5.36 | 1.62 | 1.86 | 0.07 | **0.15** | 0.94 | 2.74 | 319 | 499 |  |
| $\alpha_{21}$ | -1.02 | -1.04 | 0.37 | 0.37 | 0.02 | 0.01 | 0.96 | 0.13 | 319 | 499 |  |
| $\alpha_{22}$ | 0.70 | 0.75 | 0.50 | 0.54 | 0.07 | 0.08 | 0.95 | 0.25 | 319 | 499 |  |
| Logit | 1.15 | 1.14 | 0.16 | 0.18 | -0.01 | **0.16** | 0.95 | 0.03 | 319 | 499 |  |
| $\mu_{T1}$ | 14.32 | 14.38 | 0.35 | 0.38 | 0.00 | 0.08 | 0.96 | 0.13 | 319 | 499 |  |
| $\mu_{T2}$ | 17.79 | 17.98 | 0.52 | 0.56 | 0.01 | 0.09 | 0.91 | 0.30 | 319 | 499 |  |
| $\delta_{T1}$ | 6.00 | 5.93 | 0.48 | 0.54 | -0.01 | **0.12** | 0.94 | 0.24 | 319 | 499 |  |
| $\delta_{T2}$ | 6.00 | 5.78 | 0.73 | 0.75 | -0.04 | 0.02 | 0.95 | 0.58 | 319 | 499 |  |
| $\lambda_{21}$ | 1.05 | 1.05 | 0.02 | 0.02 | 0.00 | 0.01 | 0.96 | 0.00 | 319 | 499 |  |
| $\lambda_{22}$ | 0.94 | 0.94 | 0.02 | 0.02 | 0.00 | 0.07 | 0.95 | 0.00 | 319 | 499 |  |
| $Var(E_{11})$ | 2.30 | 2.34 | 0.23 | 0.23 | 0.02 | 0.02 | 0.95 | 0.05 | 319 | 499 |  |
| $Var(E_{12})$ | 1.06 | 1.07 | 0.21 | 0.21 | 0.01 | -0.03 | 0.97 | 0.05 | 319 | 499 |  |
| $Var(E_{21})$ | 1.42 | 1.43 | 0.09 | 0.09 | 0.01 | 0.01 | 0.92 | 0.01 | 319 | 499 |  |
| $Var(E_{22})$ | 0.64 | 0.64 | 0.07 | 0.08 | 0.01 | **0.13** | 0.95 | 0.01 | 319 | 499 |  |
| $Var({IST}_{21})$ | 0.63 | 0.63 | 0.13 | 0.12 | 0.00 | -0.08 | 0.96 | 0.02 | 319 | 499 |  |
| $Var({IST}_{22})$ | 0.33 | 0.32 | 0.11 | 0.11 | -0.03 | 0.08 | 0.93 | 0.01 | 319 | 499 |  |
| $Var(O_{1})$ | 9.97 | 10.12 | 0.68 | 0.73 | 0.01 | 0.08 | 0.94 | 0.48 | 319 | 499 |  |
| $Var(O_{2})$ | 0.78 | 0.79 | 0.14 | 0.16 | 0.02 | **0.12** | 0.97 | 0.02 | 319 | 499 |  |
| $\sigma_{T1}^{2}$ | 1.59 | 1.71 | 1.00 | 1.06 | 0.08 | 0.06 | 0.94 | 1.02 | 319 | 499 |  |
| $\sigma_{T2}^{2}$ | 0.64 | 1.60 | 2.01 | 1.70 | **1.50** | **-0.15** | 0.93 | 4.96 | 319 | 499 |  |
| *Note*. Absolute *peb* and *seb* values exceeding the cut-offs for at least medium bias (> 0.10) are printed in bold*. c =* index for class; $\alpha_{2c}$= intercept for the second indicator; $\delta_{Tc}$= skewness*; E_jc_* = residual variable at occasion *j*; Estimate = mean of estimates of all included replications; $IST_{2c}$ = indicator-specific trait factor*;*$\lambda_{2c}$ = trait loading*;MSE* = mean squared error;$O_{c}$= occasion-specific factor*;peb* = parameter estimation bias; Pop. = population value; reps = replications; *SD* = standard deviation of parameter estimates; *SE* = standard error; *seb* = standard error bias; *T*_c_ = latent trait variable*;*$\nu_{c}$= degrees of freedom parameter; $\mu_{Tc}$ = location parameter for the latent trait variable; $\sigma_{Tc}^{2}$ = scale parameter for the latent trait variable. | | | | | | | | | | | |

Table S 18. *Results for the Simulation Study for the Skew t Mixture Latent State-Trait Model with* $\boldsymbol{\delta}_{\boldsymbol{Tc}}\boldsymbol{=6}$*, 5 Occasions and 1,000 Observations*

| Parameter | Pop. | Estimate | *SD* | *SE* | *peb* | *seb* | 95% coverage | *MSE* | included reps | converged reps |  |
| --- | --- | --- | --- | --- | --- | --- | --- | --- | --- | --- | --- |
| $\nu_{1}$ | 5.00 | 5.08 | 0.37 | 0.37 | 0.02 | 0.01 | 0.95 | 0.14 | 398 | 500 |  |
| $\nu_{2}$ | 5.00 | 5.09 | 0.97 | 0.96 | 0.02 | -0.01 | 0.96 | 0.95 | 398 | 500 |  |
| $\alpha_{21}$ | -1.02 | -1.04 | 0.25 | 0.24 | 0.02 | -0.02 | 0.96 | 0.06 | 398 | 500 |  |
| $\alpha_{22}$ | 0.70 | 0.70 | 0.36 | 0.35 | 0.01 | -0.03 | 0.97 | 0.13 | 398 | 500 |  |
| Logit | 1.15 | 1.14 | 0.11 | 0.12 | -0.01 | 0.06 | 0.95 | 0.01 | 398 | 500 |  |
| $\mu_{T1}$ | 14.32 | 14.34 | 0.24 | 0.25 | 0.00 | 0.05 | 0.95 | 0.06 | 398 | 500 |  |
| $\mu_{T2}$ | 17.79 | 17.86 | 0.34 | 0.38 | 0.00 | **0.13** | 0.94 | 0.12 | 398 | 500 |  |
| $\delta_{T1}$ | 6.00 | 5.99 | 0.34 | 0.35 | 0.00 | 0.04 | 0.92 | 0.11 | 398 | 500 |  |
| $\delta_{T2}$ | 6.00 | 5.91 | 0.48 | 0.54 | -0.01 | **0.12** | 0.95 | 0.24 | 398 | 500 |  |
| $\lambda_{21}$ | 1.05 | 1.05 | 0.01 | 0.01 | 0.00 | -0.01 | 0.96 | 0.00 | 398 | 500 |  |
| $\lambda_{22}$ | 0.94 | 0.94 | 0.02 | 0.02 | 0.00 | -0.03 | 0.92 | 0.00 | 398 | 500 |  |
| $Var(E_{11})$ | 2.30 | 2.32 | 0.16 | 0.16 | 0.01 | 0.01 | 0.93 | 0.03 | 398 | 500 |  |
| $Var(E_{12})$ | 1.06 | 1.07 | 0.14 | 0.14 | 0.01 | 0.03 | 0.96 | 0.02 | 398 | 500 |  |
| $Var(E_{21})$ | 1.42 | 1.43 | 0.06 | 0.07 | 0.00 | 0.04 | 0.93 | 0.00 | 398 | 500 |  |
| $Var(E_{22})$ | 0.64 | 0.64 | 0.05 | 0.05 | 0.00 | 0.03 | 0.96 | 0.00 | 398 | 500 |  |
| $Var({IST}_{21})$ | 0.63 | 0.63 | 0.08 | 0.08 | 0.00 | 0.01 | 0.96 | 0.01 | 398 | 500 |  |
| $Var({IST}_{22})$ | 0.33 | 0.32 | 0.08 | 0.07 | -0.02 | -0.04 | 0.94 | 0.01 | 398 | 500 |  |
| $Var(O_{1})$ | 9.97 | 10.03 | 0.47 | 0.49 | 0.01 | 0.04 | 0.94 | 0.22 | 398 | 500 |  |
| $Var(O_{2})$ | 0.78 | 0.79 | 0.10 | 0.10 | 0.01 | 0.06 | 0.96 | 0.01 | 398 | 500 |  |
| $\sigma_{T1}^{2}$ | 1.59 | 1.57 | 0.74 | 0.72 | -0.01 | -0.02 | 0.95 | 0.54 | 398 | 500 |  |
| $\sigma_{T2}^{2}$ | 0.64 | 1.08 | 1.29 | 1.25 | **0.68** | -0.03 | 0.94 | 1.85 | 398 | 500 |  |
| *Note*. Absolute *peb* and *seb* values exceeding the cut-offs for at least medium bias (> 0.10) are printed in bold*. c =* index for class; $\alpha_{2c}$= intercept for the second indicator; $\delta_{Tc}$= skewness*; E_jc_* = residual variable at occasion *j*; Estimate = mean of estimates of all included replications; $IST_{2c}$ = indicator-specific trait factor*;*$\lambda_{2c}$ = trait loading*;MSE* = mean squared error;$O_{c}$= occasion-specific factor*;peb* = parameter estimation bias; Pop. = population value; reps = replications; *SD* = standard deviation of parameter estimates; *SE* = standard error; *seb* = standard error bias; *T*_c_ = latent trait variable*;*$\nu_{c}$= degrees of freedom parameter; $\mu_{Tc}$ = location parameter for the latent trait variable; $\sigma_{Tc}^{2}$ = scale parameter for the latent trait variable. | | | | | | | | | | | |

Table S 19. *Results for the Simulation Study for the Skew t Mixture Latent State-Trait Model with* $\boldsymbol{\delta}_{\boldsymbol{Tc}}\boldsymbol{=6}$*, 6 Occasions and 125 Observations*

| Parameter | Pop. | Estimate | *SD* | *SE* | *peb* | *seb* | 95% coverage | *MSE* | included reps | converged reps |  |
| --- | --- | --- | --- | --- | --- | --- | --- | --- | --- | --- | --- |
| $\nu_{1}$ | 5.00 | 5.38 | 1.18 | 1.27 | 0.08 | 0.07 | 0.93 | 1.54 | 136 | 494 |  |
| $\nu_{2}$ | 5.00 | 17.60 | 123.37 | 32.25 | **2.52** | **-0.74** | 0.90 | 15378.27 | 136 | 494 |  |
| $\alpha_{21}$ | -1.02 | -1.02 | 0.64 | 0.74 | 0.00 | **0.15** | 0.94 | 0.41 | 136 | 494 |  |
| $\alpha_{22}$ | 0.70 | 0.56 | 1.21 | 1.24 | **-0.21** | 0.03 | 0.93 | 1.48 | 136 | 494 |  |
| Logit | 1.15 | 1.13 | 0.26 | 0.39 | -0.02 | **0.50** | 0.97 | 0.07 | 136 | 494 |  |
| $\mu_{T1}$ | 14.32 | 14.57 | 0.89 | 0.75 | 0.02 | -0.15 | 0.98 | 0.85 | 136 | 494 |  |
| $\mu_{T2}$ | 17.79 | 18.71 | 1.80 | 1.82 | 0.05 | 0.01 | 0.93 | 4.07 | 136 | 494 |  |
| $\delta_{T1}$ | 6.00 | 5.80 | 1.17 | 1.09 | -0.03 | -0.07 | 0.86 | 1.41 | 136 | 494 |  |
| $\delta_{T2}$ | 6.00 | 4.92 | 2.29 | 1.93 | **-0.18** | **-0.16** | 0.96 | 6.40 | 136 | 494 |  |
| $\lambda_{21}$ | 1.05 | 1.05 | 0.03 | 0.04 | 0.00 | **0.12** | 0.95 | 0.00 | 136 | 494 |  |
| $\lambda_{22}$ | 0.94 | 0.95 | 0.05 | 0.05 | 0.01 | 0.01 | 0.99 | 0.00 | 136 | 494 |  |
| $Var(E_{11})$ | 2.30 | 2.42 | 0.47 | 0.52 | 0.05 | **0.11** | 0.93 | 0.24 | 136 | 494 |  |
| $Var(E_{12})$ | 1.06 | 1.16 | 0.40 | 0.45 | 0.09 | **0.11** | 0.99 | 0.17 | 136 | 494 |  |
| $Var(E_{21})$ | 1.42 | 1.44 | 0.16 | 0.19 | 0.01 | **0.18** | 0.96 | 0.03 | 136 | 494 |  |
| $Var(E_{22})$ | 0.64 | 0.66 | 0.15 | 0.17 | 0.03 | **0.14** | 0.95 | 0.02 | 136 | 494 |  |
| $Var({IST}_{21})$ | 0.63 | 0.61 | 0.20 | 0.21 | -0.03 | 0.05 | 0.89 | 0.04 | 136 | 494 |  |
| $Var({IST}_{22})$ | 0.33 | 0.30 | 0.19 | 0.20 | **-0.10** | **0.10** | 0.96 | 0.04 | 136 | 494 |  |
| $Var(O_{1})$ | 9.97 | 10.16 | 1.26 | 1.50 | 0.02 | **0.19** | 0.91 | 1.63 | 136 | 494 |  |
| $Var(O_{2})$ | 0.78 | 0.80 | 0.28 | 0.34 | 0.02 | **0.21** | 0.98 | 0.08 | 136 | 494 |  |
| $\sigma_{T1}^{2}$ | 1.59 | 2.30 | 2.02 | 2.12 | **0.45** | 0.05 | 0.92 | 4.59 | 136 | 494 |  |
| $\sigma_{T2}^{2}$ | 0.64 | 3.00 | 3.79 | 2.92 | **3.69** | -**0.23** | 0.90 | 19.95 | 136 | 494 |  |
| *Note*. Absolute *peb* and *seb* values exceeding the cut-offs for at least medium bias (> 0.10) are printed in bold*. c =* index for class; $\alpha_{2c}$= intercept for the second indicator; $\delta_{Tc}$= skewness*; E_jc_* = residual variable at occasion *j*; Estimate = mean of estimates of all included replications; $IST_{2c}$ = indicator-specific trait factor*;*$\lambda_{2c}$ = trait loading*;MSE* = mean squared error;$O_{c}$= occasion-specific factor*;peb* = parameter estimation bias; Pop. = population value; reps = replications; *SD* = standard deviation of parameter estimates; *SE* = standard error; *seb* = standard error bias; *T*_c_ = latent trait variable*;*$\nu_{c}$= degrees of freedom parameter; $\mu_{Tc}$ = location parameter for the latent trait variable; $\sigma_{Tc}^{2}$ = scale parameter for the latent trait variable. | | | | | | | | | | | |

Table S 20. *Results for the Simulation Study for the Skew t Mixture Latent State-Trait Model with* $\boldsymbol{\delta}_{\boldsymbol{Tc}}\boldsymbol{=6}$*, 6 Occasions and 250 Observations*

| Parameter | Pop. | Estimate | *SD* | *SE* | *peb* | *seb* | 95% coverage | *MSE* | included reps | converged reps |  |
| --- | --- | --- | --- | --- | --- | --- | --- | --- | --- | --- | --- |
| $\nu_{1}$ | 5.00 | 5.19 | 0.74 | 0.79 | 0.04 | 0.07 | 0.96 | 0.58 | 251 | 498 |  |
| $\nu_{2}$ | 5.00 | 8.83 | 47.39 | 11.62 | **0.77** | **-0.75** | 0.96 | 2260.45 | 251 | 498 |  |
| $\alpha_{21}$ | -1.02 | -1.04 | 0.41 | 0.58 | 0.02 | **0.40** | 0.96 | 0.17 | 251 | 498 |  |
| $\alpha_{22}$ | 0.70 | 0.75 | 0.75 | 0.76 | 0.07 | 0.00 | 0.95 | 0.57 | 251 | 498 |  |
| Logit | 1.15 | 1.15 | 0.19 | 0.31 | 0.00 | **0.61** | 0.97 | 0.04 | 251 | 498 |  |
| $\mu_{T1}$ | 14.32 | 14.41 | 0.49 | 0.59 | 0.01 | **0.20** | 0.96 | 0.25 | 251 | 498 |  |
| $\mu_{T2}$ | 17.79 | 18.08 | 0.74 | 0.94 | 0.02 | **0.28** | 0.96 | 0.63 | 251 | 498 |  |
| $\delta_{T1}$ | 6.00 | 5.97 | 0.67 | 1.02 | 0.00 | **0.52** | 0.90 | 0.45 | 251 | 498 |  |
| $\delta_{T2}$ | 6.00 | 5.65 | 1.01 | 1.39 | -0.06 | **0.37** | 0.95 | 1.15 | 251 | 498 |  |
| $\lambda_{21}$ | 1.05 | 1.05 | 0.02 | 0.03 | 0.00 | **0.39** | 0.94 | 0.00 | 251 | 498 |  |
| $\lambda_{22}$ | 0.94 | 0.94 | 0.03 | 0.03 | 0.00 | -0.01 | 0.98 | 0.00 | 251 | 498 |  |
| $Var(E_{11})$ | 2.30 | 2.32 | 0.32 | 0.36 | 0.01 | **0.12** | 0.95 | 0.10 | 251 | 498 |  |
| $Var(E_{12})$ | 1.06 | 1.09 | 0.26 | 0.28 | 0.03 | 0.06 | 0.96 | 0.07 | 251 | 498 |  |
| $Var(E_{21})$ | 1.42 | 1.43 | 0.12 | 0.12 | 0.01 | 0.03 | 0.96 | 0.01 | 251 | 498 |  |
| $Var(E_{22})$ | 0.64 | 0.65 | 0.10 | 0.11 | 0.01 | **0.17** | 0.97 | 0.01 | 251 | 498 |  |
| $Var({IST}_{21})$ | 0.63 | 0.61 | 0.13 | 0.15 | -0.03 | 0.09 | 0.96 | 0.02 | 251 | 498 |  |
| $Var({IST}_{22})$ | 0.33 | 0.31 | 0.13 | 0.14 | -0.08 | 0.02 | 0.96 | 0.02 | 251 | 498 |  |
| $Var(O_{1})$ | 9.97 | 10.06 | 0.86 | 1.08 | 0.01 | **0.25** | 0.92 | 0.75 | 251 | 498 |  |
| $Var(O_{2})$ | 0.78 | 0.77 | 0.17 | 0.24 | -0.01 | **0.47** | 0.96 | 0.03 | 251 | 498 |  |
| $\sigma_{T1}^{2}$ | 1.59 | 1.81 | 1.27 | 1.49 | 0.14 | **0.17** | 0.96 | 1.66 | 251 | 498 |  |
| $\sigma_{T2}^{2}$ | 0.64 | 1.75 | 2.30 | 2.05 | **1.73** | **-0.11** | 0.92 | 6.54 | 251 | 498 |  |
| *Note*. Absolute *peb* and *seb* values exceeding the cut-offs for at least medium bias (> 0.10) are printed in bold*. c =* index for class; $\alpha_{2c}$= intercept for the second indicator; $\delta_{Tc}$= skewness*; E_jc_* = residual variable at occasion *j*; Estimate = mean of estimates of all included replications; $IST_{2c}$ = indicator-specific trait factor*;*$\lambda_{2c}$ = trait loading*;MSE* = mean squared error;$O_{c}$= occasion-specific factor*;peb* = parameter estimation bias; Pop. = population value; reps = replications; *SD* = standard deviation of parameter estimates; *SE* = standard error; *seb* = standard error bias; *T*_c_ = latent trait variable*;*$\nu_{c}$= degrees of freedom parameter; $\mu_{Tc}$ = location parameter for the latent trait variable; $\sigma_{Tc}^{2}$ = scale parameter for the latent trait variable. | | | | | | | | | | | |

Table S 21. *Results for the Simulation Study for the Skew t Mixture Latent State-Trait Model with* $\boldsymbol{\delta}_{\boldsymbol{Tc}}\boldsymbol{=6}$*, 6 Occasions and 500 Observations*

| Parameter | Pop. | Estimate | *SD* | *SE* | *peb* | *seb* | 95% coverage | *MSE* | included reps | converged reps |  |
| --- | --- | --- | --- | --- | --- | --- | --- | --- | --- | --- | --- |
| $\nu_{1}$ | 5.00 | 5.10 | 0.50 | 0.51 | 0.02 | 0.02 | 0.94 | 0.26 | 342 | 500 |  |
| $\nu_{2}$ | 5.00 | 5.23 | 1.73 | 1.34 | 0.05 | **-0.22** | 0.98 | 3.04 | 342 | 500 |  |
| $\alpha_{21}$ | -1.02 | -1.02 | 0.29 | 0.32 | 0.00 | **0.11** | 0.96 | 0.08 | 342 | 500 |  |
| $\alpha_{22}$ | 0.70 | 0.69 | 0.49 | 0.48 | -0.01 | -0.02 | 0.93 | 0.24 | 342 | 500 |  |
| Logit | 1.15 | 1.14 | 0.14 | 0.15 | -0.01 | 0.09 | 0.95 | 0.02 | 342 | 500 |  |
| $\mu_{T1}$ | 14.32 | 14.35 | 0.35 | 0.34 | 0.00 | -0.05 | 0.97 | 0.13 | 342 | 500 |  |
| $\mu_{T2}$ | 17.79 | 17.90 | 0.43 | 0.49 | 0.01 | 0.15 | 0.95 | 0.20 | 342 | 500 |  |
| $\delta_{T1}$ | 6.00 | 6.01 | 0.47 | 0.47 | 0.00 | 0.00 | 0.93 | 0.22 | 342 | 500 |  |
| $\delta_{T2}$ | 6.00 | 5.86 | 0.59 | 0.69 | -0.02 | **0.17** | 0.96 | 0.37 | 342 | 500 |  |
| $\lambda_{21}$ | 1.05 | 1.05 | 0.02 | 0.02 | 0.00 | 0.09 | 0.96 | 0.00 | 342 | 500 |  |
| $\lambda_{22}$ | 0.94 | 0.94 | 0.02 | 0.02 | 0.00 | -0.02 | 0.96 | 0.00 | 342 | 500 |  |
| $Var(E_{11})$ | 2.30 | 2.31 | 0.21 | 0.22 | 0.01 | 0.05 | 0.95 | 0.05 | 342 | 500 |  |
| $Var(E_{12})$ | 1.06 | 1.09 | 0.20 | 0.19 | 0.03 | -0.02 | 0.96 | 0.04 | 342 | 500 |  |
| $Var(E_{21})$ | 1.42 | 1.43 | 0.08 | 0.08 | 0.00 | 0.06 | 0.95 | 0.01 | 342 | 500 |  |
| $Var(E_{22})$ | 0.64 | 0.65 | 0.07 | 0.07 | 0.01 | 0.07 | 0.95 | 0.00 | 342 | 500 |  |
| $Var({IST}_{21})$ | 0.63 | 0.63 | 0.10 | 0.10 | -0.01 | 0.06 | 0.96 | 0.01 | 342 | 500 |  |
| $Var({IST}_{22})$ | 0.33 | 0.33 | 0.09 | 0.10 | -0.01 | 0.01 | 0.97 | 0.01 | 342 | 500 |  |
| $Var(O_{1})$ | 9.97 | 10.08 | 0.57 | 0.63 | 0.01 | 0.10 | 0.94 | 0.34 | 342 | 500 |  |
| $Var(O_{2})$ | 0.78 | 0.79 | 0.12 | 0.13 | 0.01 | 0.08 | 0.94 | 0.01 | 342 | 500 |  |
| $\sigma_{T1}^{2}$ | 1.59 | 1.70 | 0.96 | 0.95 | 0.07 | -0.01 | 0.97 | 0.93 | 342 | 500 |  |
| $\sigma_{T2}^{2}$ | 0.64 | 1.14 | 1.37 | 1.33 | **0.78** | -0.03 | 0.92 | 2.13 | 342 | 500 |  |
| *Note*. Absolute *peb* and *seb* values exceeding the cut-offs for at least medium bias (> 0.10) are printed in bold*. c =* index for class; $\alpha_{2c}$= intercept for the second indicator; $\delta_{Tc}$= skewness*; E_jc_* = residual variable at occasion *j*; Estimate = mean of estimates of all included replications; $IST_{2c}$ = indicator-specific trait factor*;*$\lambda_{2c}$ = trait loading*;MSE* = mean squared error;$O_{c}$= occasion-specific factor*;peb* = parameter estimation bias; Pop. = population value; reps = replications; *SD* = standard deviation of parameter estimates; *SE* = standard error; *seb* = standard error bias; *T*_c_ = latent trait variable*;*$\nu_{c}$= degrees of freedom parameter; $\mu_{Tc}$ = location parameter for the latent trait variable; $\sigma_{Tc}^{2}$ = scale parameter for the latent trait variable. | | | | | | | | | | | |

Table S 22. *Results for the Simulation Study for the Skew t Mixture Latent State-Trait Model with* $\boldsymbol{\delta}_{\boldsymbol{Tc}}\boldsymbol{=6}$*, 6 Occasions and 1,000 Observations*

| Parameter | Pop. | Estimate | *SD* | *SE* | *peb* | *seb* | 95% coverage | *MSE* | included reps | converged reps |  |
| --- | --- | --- | --- | --- | --- | --- | --- | --- | --- | --- | --- |
| $\nu_{1}$ | 5.00 | 5.08 | 0.35 | 0.35 | 0.02 | 0.01 | 0.94 | 0.13 | 417 | 500 |  |
| $\nu_{2}$ | 5.00 | 5.06 | 0.82 | 0.87 | 0.01 | 0.07 | 0.96 | 0.67 | 417 | 500 |  |
| $\alpha_{21}$ | -1.02 | -1.02 | 0.22 | 0.22 | 0.00 | 0.02 | 0.95 | 0.05 | 417 | 500 |  |
| $\alpha_{22}$ | 0.70 | 0.71 | 0.33 | 0.33 | 0.01 | -0.02 | 0.94 | 0.11 | 417 | 500 |  |
| Logit | 1.15 | 1.15 | 0.10 | 0.11 | 0.00 | 0.09 | 0.96 | 0.01 | 417 | 500 |  |
| $\mu_{T1}$ | 14.32 | 14.32 | 0.24 | 0.23 | 0.00 | -0.03 | 0.96 | 0.06 | 417 | 500 |  |
| $\mu_{T2}$ | 17.79 | 17.85 | 0.28 | 0.33 | 0.00 | **0.19** | 0.96 | 0.08 | 417 | 500 |  |
| $\delta_{T1}$ | 6.00 | 6.01 | 0.32 | 0.33 | 0.00 | 0.00 | 0.93 | 0.11 | 417 | 500 |  |
| $\delta_{T2}$ | 6.00 | 5.95 | 0.42 | 0.48 | -0.01 | **0.16** | 0.95 | 0.18 | 417 | 500 |  |
| $\lambda_{21}$ | 1.05 | 1.05 | 0.01 | 0.01 | 0.00 | 0.01 | 0.97 | 0.00 | 417 | 500 |  |
| $\lambda_{22}$ | 0.94 | 0.94 | 0.01 | 0.01 | 0.00 | -0.01 | 0.92 | 0.00 | 417 | 500 |  |
| $Var(E_{11})$ | 2.30 | 2.31 | 0.15 | 0.16 | 0.01 | 0.02 | 0.94 | 0.02 | 417 | 500 |  |
| $Var(E_{12})$ | 1.06 | 1.07 | 0.13 | 0.13 | 0.01 | -0.01 | 0.94 | 0.02 | 417 | 500 |  |
| $Var(E_{21})$ | 1.42 | 1.42 | 0.05 | 0.06 | 0.00 | 0.08 | 0.94 | 0.00 | 417 | 500 |  |
| $Var(E_{22})$ | 0.64 | 0.64 | 0.05 | 0.05 | 0.00 | 0.03 | 0.95 | 0.00 | 417 | 500 |  |
| $Var({IST}_{21})$ | 0.63 | 0.63 | 0.07 | 0.07 | 0.00 | 0.04 | 0.96 | 0.00 | 417 | 500 |  |
| $Var({IST}_{22})$ | 0.33 | 0.33 | 0.07 | 0.07 | -0.01 | 0.00 | 0.95 | 0.00 | 417 | 500 |  |
| $Var(O_{1})$ | 9.97 | 10.03 | 0.42 | 0.44 | 0.01 | 0.03 | 0.94 | 0.18 | 417 | 500 |  |
| $Var(O_{2})$ | 0.78 | 0.79 | 0.09 | 0.09 | 0.01 | 0.01 | 0.95 | 0.01 | 417 | 500 |  |
| $\sigma_{T1}^{2}$ | 1.59 | 1.62 | 0.69 | 0.66 | 0.02 | -0.05 | 0.95 | 0.48 | 417 | 500 |  |
| $\sigma_{T2}^{2}$ | 0.64 | 0.85 | 0.77 | 0.98 | **0.33** | **0.26** | 0.94 | 0.64 | 417 | 500 |  |
| *Note*. Absolute *peb* and *seb* values exceeding the cut-offs for at least medium bias (> 0.10) are printed in bold*. c =* index for class; $\alpha_{2c}$= intercept for the second indicator; $\delta_{Tc}$= skewness*; E_jc_* = residual variable at occasion *j*; Estimate = mean of estimates of all included replications; $IST_{2c}$ = indicator-specific trait factor*;*$\lambda_{2c}$ = trait loading*;MSE* = mean squared error;$O_{c}$= occasion-specific factor*;peb* = parameter estimation bias; Pop. = population value; reps = replications; *SD* = standard deviation of parameter estimates; *SE* = standard error; *seb* = standard error bias; *T*_c_ = latent trait variable*;*$\nu_{c}$= degrees of freedom parameter; $\mu_{Tc}$ = location parameter for the latent trait variable; $\sigma_{Tc}^{2}$ = scale parameter for the latent trait variable. | | | | | | | | | | | |

Table S 23. *Results for the Simulation Study for the Skew t Mixture Latent State-Trait Model with* $\boldsymbol{\delta}_{\boldsymbol{Tc}}\boldsymbol{=2.8}$*, 2 Occasions and 500 Observations*

| Parameter | Pop. | Estimate | *SD* | *SE* | *peb* | *seb* | 95% coverage | *MSE* | included reps | converged reps |  |
| --- | --- | --- | --- | --- | --- | --- | --- | --- | --- | --- | --- |
| $\nu_{1}$ | 5.00 | 5.53 | 1.07 | 1.17 | **0.11** | 0.09 | 0.98 | 1.43 | 112 | 492 |  |
| $\nu_{2}$ | 5.00 | 64.78 | 245.87 | 64.96 | **11.96** | **-0.74** | 0.92 | 64026.70 | 112 | 492 |  |
| $\alpha_{21}$ | -1.02 | -1.00 | 1.28 | 1.65 | -0.02 | **0.29** | 0.97 | 1.64 | 112 | 492 |  |
| $\alpha_{22}$ | 0.70 | 1.04 | 2.74 | 3.76 | **0.49** | **0.37** | 0.96 | 7.64 | 112 | 492 |  |
| Logit | 1.15 | 1.18 | 0.31 | 0.65 | 0.02 | **1.07** | 0.96 | 0.10 | 112 | 492 |  |
| $\mu_{T1}$ | 14.32 | 14.51 | 0.80 | 1.18 | 0.01 | **0.47** | 0.90 | 0.68 | 112 | 492 |  |
| $\mu_{T2}$ | 17.79 | 18.16 | 1.19 | 1.40 | 0.02 | **0.18** | 0.98 | 1.55 | 112 | 492 |  |
| $\delta_{T1}$ | 2.80 | 2.68 | 0.92 | 1.58 | -0.04 | **0.71** | 1.00 | 0.87 | 112 | 492 |  |
| $\delta_{T2}$ | 2.80 | 2.06 | 1.51 | 1.88 | **-0.27** | **0.24** | 0.97 | 2.84 | 112 | 492 |  |
| $\lambda_{21}$ | 1.05 | 1.05 | 0.08 | 0.10 | 0.00 | **0.31** | 0.96 | 0.01 | 112 | 492 |  |
| $\lambda_{22}$ | 0.94 | 0.92 | 0.14 | 0.19 | -0.02 | **0.36** | 0.98 | 0.02 | 112 | 492 |  |
| $Var(E_{11})$ | 2.30 | 2.39 | 0.31 | 0.38 | 0.04 | **0.24** | 0.99 | 0.10 | 112 | 492 |  |
| $Var(E_{12})$ | 1.06 | 1.04 | 0.37 | 0.47 | -0.02 | **0.29** | 0.99 | 0.13 | 112 | 492 |  |
| $Var(E_{21})$ | 1.42 | 1.47 | 0.24 | 0.28 | 0.03 | **0.20** | 0.94 | 0.06 | 112 | 492 |  |
| $Var(E_{22})$ | 0.64 | 0.56 | 0.25 | 0.36 | **-0.12** | **0.46** | 0.97 | 0.07 | 112 | 492 |  |
| $Var({IST}_{21})$ | 0.63 | 0.57 | 0.28 | 0.33 | **-0.10** | **0.21** | 0.91 | 0.08 | 112 | 492 |  |
| $Var({IST}_{22})$ | 0.33 | 0.42 | 0.28 | 0.44 | **0.28** | **0.58** | 0.97 | 0.09 | 112 | 492 |  |
| $Var(O_{1})$ | 9.97 | 10.11 | 1.25 | 1.92 | 0.01 | **0.53** | 0.90 | 1.59 | 112 | 492 |  |
| $Var(O_{2})$ | 0.78 | 0.71 | 0.34 | 0.69 | -0.09 | **1.05** | 0.97 | 0.12 | 112 | 492 |  |
| $\sigma_{T1}^{2}$ | 1.59 | 2.18 | 1.31 | 2.15 | **0.37** | **0.64** | 0.96 | 2.06 | 112 | 492 |  |
| $\sigma_{T2}^{2}$ | 0.64 | 2.10 | 1.93 | 3.10 | **2.29** | **0.60** | 0.90 | 5.87 | 112 | 492 |  |
| *Note*. Absolute *peb* and *seb* values exceeding the cut-offs for at least medium bias (> 0.10) are printed in bold*. c =* index for class; $\alpha_{2c}$= intercept for the second indicator; $\delta_{Tc}$= skewness*; E_jc_* = residual variable at occasion *j*; Estimate = mean of estimates of all included replications; $IST_{2c}$ = indicator-specific trait factor*;*$\lambda_{2c}$ = trait loading*;MSE* = mean squared error;$O_{c}$= occasion-specific factor*;peb* = parameter estimation bias; Pop. = population value; reps = replications; *SD* = standard deviation of parameter estimates; *SE* = standard error; *seb* = standard error bias; *T*_c_ = latent trait variable*;*$\nu_{c}$= degrees of freedom parameter; $\mu_{Tc}$ = location parameter for the latent trait variable; $\sigma_{Tc}^{2}$ = scale parameter for the latent trait variable. | | | | | | | | | | | |

Table S 24. *Results for the Simulation Study for the Skew t Mixture Latent State-Trait Model with* $\boldsymbol{\delta}_{\boldsymbol{Tc}}\boldsymbol{=2.8}$*, 2 Occasions and 1,000 Observations*

| Parameter | Pop. | Estimate | *SD* | *SE* | *peb* | *seb* | 95% coverage | *MSE* | included reps | converged reps |  |
| --- | --- | --- | --- | --- | --- | --- | --- | --- | --- | --- | --- |
| $\nu_{1}$ | 5.00 | 5.21 | 0.58 | 0.65 | 0.04 | **0.12** | 0.98 | 0.38 | 237 | 494 |  |
| $\nu_{2}$ | 5.00 | 10.41 | 50.25 | 102.88 | **1.08** | **1.05** | 0.98 | 2553.92 | 237 | 494 |  |
| $\alpha_{21}$ | -1.02 | -1.15 | 1.02 | 1.14 | **0.12** | **0.12** | 0.96 | 1.05 | 237 | 494 |  |
| $\alpha_{22}$ | 0.70 | 0.80 | 1.48 | 1.88 | **0.14** | **0.27** | 0.95 | 2.19 | 237 | 494 |  |
| Logit | 1.15 | 1.13 | 0.22 | 0.37 | -0.02 | **0.68** | 0.97 | 0.05 | 237 | 494 |  |
| $\mu_{T1}$ | 14.32 | 14.38 | 0.48 | 0.61 | 0.00 | **0.26** | 0.96 | 0.24 | 237 | 494 |  |
| $\mu_{T2}$ | 17.79 | 17.97 | 0.60 | 0.73 | 0.01 | **0.23** | 0.97 | 0.39 | 237 | 494 |  |
| $\delta_{T1}$ | 2.80 | 2.76 | 0.61 | 0.80 | -0.02 | **0.31** | 0.99 | 0.38 | 237 | 494 |  |
| $\delta_{T2}$ | 2.80 | 2.43 | 0.84 | 1.13 | **-0.13** | **0.33** | 0.97 | 0.85 | 237 | 494 |  |
| $\lambda_{21}$ | 1.05 | 1.06 | 0.06 | 0.07 | 0.01 | **0.13** | 0.95 | 0.00 | 237 | 494 |  |
| $\lambda_{22}$ | 0.94 | 0.94 | 0.07 | 0.09 | 0.00 | **0.27** | 0.98 | 0.01 | 237 | 494 |  |
| $Var(E_{11})$ | 2.30 | 2.34 | 0.21 | 0.25 | 0.02 | **0.18** | 0.95 | 0.05 | 237 | 494 |  |
| $Var(E_{12})$ | 1.06 | 1.06 | 0.24 | 0.29 | 0.00 | **0.24** | 0.97 | 0.06 | 237 | 494 |  |
| $Var(E_{21})$ | 1.42 | 1.44 | 0.14 | 0.17 | 0.02 | **0.20** | 0.93 | 0.02 | 237 | 494 |  |
| $Var(E_{22})$ | 0.64 | 0.62 | 0.17 | 0.21 | -0.04 | **0.25** | 0.97 | 0.03 | 237 | 494 |  |
| $Var({IST}_{21})$ | 0.63 | 0.60 | 0.21 | 0.23 | -0.04 | **0.11** | 0.98 | 0.05 | 237 | 494 |  |
| $Var({IST}_{22})$ | 0.33 | 0.36 | 0.20 | 0.29 | 0.09 | **0.43** | 0.97 | 0.04 | 237 | 494 |  |
| $Var(O_{1})$ | 9.97 | 10.12 | 0.86 | 1.17 | 0.01 | **0.35** | 0.95 | 0.76 | 237 | 494 |  |
| $Var(O_{2})$ | 0.78 | 0.78 | 0.28 | 0.36 | 0.00 | **0.29** | 0.98 | 0.08 | 237 | 494 |  |
| $\sigma_{T1}^{2}$ | 1.59 | 1.76 | 0.94 | 1.31 | **0.11** | **0.38** | 0.97 | 0.92 | 237 | 494 |  |
| $\sigma_{T2}^{2}$ | 0.64 | 1.32 | 1.35 | 2.11 | **1.07** | **0.57** | 0.95 | 2.28 | 237 | 494 |  |
| *Note*. Absolute *peb* and *seb* values exceeding the cut-offs for at least medium bias (> 0.10) are printed in bold*. c =* index for class; $\alpha_{2c}$= intercept for the second indicator; $\delta_{Tc}$= skewness*; E_jc_* = residual variable at occasion *j*; Estimate = mean of estimates of all included replications; $IST_{2c}$ = indicator-specific trait factor*;*$\lambda_{2c}$ = trait loading*;MSE* = mean squared error;$O_{c}$= occasion-specific factor*;peb* = parameter estimation bias; Pop. = population value; reps = replications; *SD* = standard deviation of parameter estimates; *SE* = standard error; *seb* = standard error bias; *T*_c_ = latent trait variable*;*$\nu_{c}$= degrees of freedom parameter; $\mu_{Tc}$ = location parameter for the latent trait variable; $\sigma_{Tc}^{2}$ = scale parameter for the latent trait variable. | | | | | | | | | | | |

Table S 25. *Results for the Simulation Study for the Skew t Mixture Latent State-Trait Model with* $\boldsymbol{\delta}_{\boldsymbol{Tc}}\boldsymbol{=2.8}$*, 3 Occasions and 125 Observations*

| Parameter | Pop. | Estimate | *SD* | *SE* | *peb* | *seb* | 95% coverage | *MSE* | included reps | converged reps |  |
| --- | --- | --- | --- | --- | --- | --- | --- | --- | --- | --- | --- |
| $\nu_{1}$ | 5.00 | 6.10 | 2.20 | 1.94 | **0.22** | **-0.12** | 0.93 | 6.07 | 82 | 490 |  |
| $\nu_{2}$ | 5.00 | 204.46 | 611.14 | 302.48 | **39.89** | **-0.51** | 0.91 | 413269.51 | 82 | 490 |  |
| $\alpha_{21}$ | -1.02 | -1.02 | 2.61 | 2.92 | 0.00 | **0.12** | 0.93 | 6.80 | 82 | 490 |  |
| $\alpha_{22}$ | 0.70 | 0.74 | 3.08 | 3.23 | 0.06 | 0.05 | 0.91 | 9.47 | 82 | 490 |  |
| Logit | 1.15 | 1.01 | 0.36 | 0.58 | **-0.13** | **0.60** | 0.98 | 0.15 | 82 | 490 |  |
| $\mu_{T1}$ | 14.32 | 14.79 | 1.20 | 1.49 | 0.03 | **0.24** | 0.84 | 1.65 | 82 | 490 |  |
| $\mu_{T2}$ | 17.79 | 18.48 | 1.57 | 1.92 | 0.04 | **0.23** | 1.00 | 2.93 | 82 | 490 |  |
| $\delta_{T1}$ | 2.80 | 2.30 | 1.44 | 1.68 | **-0.18** | **0.17** | 0.95 | 2.31 | 82 | 490 |  |
| $\delta_{T2}$ | 2.80 | 1.63 | 1.97 | 2.32 | **-0.42** | **0.18** | 0.99 | 5.23 | 82 | 490 |  |
| $\lambda_{21}$ | 1.05 | 1.05 | 0.16 | 0.18 | 0.00 | **0.13** | 0.91 | 0.02 | 82 | 490 |  |
| $\lambda_{22}$ | 0.94 | 0.94 | 0.15 | 0.16 | 0.00 | 0.08 | 0.96 | 0.02 | 82 | 490 |  |
| $Var(E_{11})$ | 2.30 | 2.41 | 0.53 | 0.61 | 0.05 | **0.15** | 0.96 | 0.29 | 82 | 490 |  |
| $Var(E_{12})$ | 1.06 | 1.04 | 0.54 | 0.70 | -0.02 | **0.28** | 0.98 | 0.30 | 82 | 490 |  |
| $Var(E_{21})$ | 1.42 | 1.49 | 0.26 | 0.32 | 0.05 | **0.25** | 0.94 | 0.07 | 82 | 490 |  |
| $Var(E_{22})$ | 0.64 | 0.64 | 0.30 | 0.31 | 0.01 | 0.03 | 0.91 | 0.09 | 82 | 490 |  |
| $Var({IST}_{21})$ | 0.63 | 0.59 | 0.35 | 0.43 | -0.06 | **0.24** | 0.91 | 0.12 | 82 | 490 |  |
| $Var({IST}_{22})$ | 0.33 | 0.41 | 0.29 | 0.50 | **0.24** | **0.76** | 0.95 | 0.09 | 82 | 490 |  |
| $Var(O_{1})$ | 9.97 | 10.87 | 1.92 | 2.66 | 0.09 | **0.39** | 0.89 | 4.52 | 82 | 490 |  |
| $Var(O_{2})$ | 0.78 | 0.82 | 0.43 | 0.53 | 0.05 | **0.21** | 0.93 | 0.19 | 82 | 490 |  |
| $\sigma_{T1}^{2}$ | 1.59 | 2.37 | 1.77 | 2.92 | **0.49** | **0.65** | 0.98 | 3.73 | 82 | 490 |  |
| $\sigma_{T2}^{2}$ | 0.64 | 2.25 | 2.03 | 3.40 | **2.51** | **0.67** | 0.89 | 6.72 | 82 | 490 |  |
| *Note*. Absolute *peb* and *seb* values exceeding the cut-offs for at least medium bias (> 0.10) are printed in bold*. c =* index for class; $\alpha_{2c}$= intercept for the second indicator; $\delta_{Tc}$= skewness*; E_jc_* = residual variable at occasion *j*; Estimate = mean of estimates of all included replications; $IST_{2c}$ = indicator-specific trait factor*;*$\lambda_{2c}$ = trait loading*;MSE* = mean squared error;$O_{c}$= occasion-specific factor*;peb* = parameter estimation bias; Pop. = population value; reps = replications; *SD* = standard deviation of parameter estimates; *SE* = standard error; *seb* = standard error bias; *T*_c_ = latent trait variable*;*$\nu_{c}$= degrees of freedom parameter; $\mu_{Tc}$ = location parameter for the latent trait variable; $\sigma_{Tc}^{2}$ = scale parameter for the latent trait variable. | | | | | | | | | | | |

Table S 26. *Results for the Simulation Study for the Skew t Mixture Latent State-Trait Model with* $\boldsymbol{\delta}_{\boldsymbol{Tc}}\boldsymbol{=2.8}$*, 3 Occasions and 250 Observations*

| Parameter | Pop. | Estimate | *SD* | *SE* | *peb* | *seb* | 95% coverage | *MSE* | included reps | converged reps |  |
| --- | --- | --- | --- | --- | --- | --- | --- | --- | --- | --- | --- |
| $\nu_{1}$ | 5.00 | 5.46 | 1.05 | 1.10 | 0.09 | 0.04 | 0.95 | 1.32 | 169 | 497 |  |
| $\nu_{2}$ | 5.00 | 53.12 | 235.03 | 75.37 | **9.62** | **-0.68** | 0.88 | 57554.23 | 169 | 497 |  |
| $\alpha_{21}$ | -1.02 | -1.11 | 1.32 | 1.63 | 0.09 | **0.23** | 0.92 | 1.74 | 169 | 497 |  |
| $\alpha_{22}$ | 0.70 | 0.85 | 2.33 | 2.59 | **0.22** | **0.11** | 0.93 | 5.46 | 169 | 497 |  |
| Logit | 1.15 | 1.12 | 0.30 | 0.41 | -0.03 | **0.38** | 0.99 | 0.09 | 169 | 497 |  |
| $\mu_{T1}$ | 14.32 | 14.58 | 0.81 | 0.91 | 0.02 | **0.13** | 0.91 | 0.72 | 169 | 497 |  |
| $\mu_{T2}$ | 17.79 | 18.33 | 1.10 | 1.17 | 0.03 | 0.07 | 0.97 | 1.49 | 169 | 497 |  |
| $\delta_{T1}$ | 2.80 | 2.56 | 1.02 | 1.11 | -0.08 | 0.09 | 0.97 | 1.09 | 169 | 497 |  |
| $\delta_{T2}$ | 2.80 | 2.01 | 1.45 | 1.59 | **-0.28** | 0.10 | 0.98 | 2.73 | 169 | 497 |  |
| $\lambda_{21}$ | 1.05 | 1.06 | 0.08 | 0.10 | 0.00 | **0.22** | 0.91 | 0.01 | 169 | 497 |  |
| $\lambda_{22}$ | 0.94 | 0.93 | 0.11 | 0.13 | -0.01 | **0.11** | 0.96 | 0.01 | 169 | 497 |  |
| $Var(E_{11})$ | 2.30 | 2.33 | 0.41 | 0.39 | 0.01 | -0.04 | 0.93 | 0.17 | 169 | 497 |  |
| $Var(E_{12})$ | 1.06 | 1.07 | 0.37 | 0.44 | 0.01 | **0.18** | 0.95 | 0.14 | 169 | 497 |  |
| $Var(E_{21})$ | 1.42 | 1.47 | 0.19 | 0.23 | 0.04 | **0.22** | 0.98 | 0.04 | 169 | 497 |  |
| $Var(E_{22})$ | 0.64 | 0.63 | 0.20 | 0.24 | -0.01 | **0.23** | 0.91 | 0.04 | 169 | 497 |  |
| $Var({IST}_{21})$ | 0.63 | 0.57 | 0.24 | 0.28 | -0.09 | **0.16** | 0.91 | 0.06 | 169 | 497 |  |
| $Var({IST}_{22})$ | 0.33 | 0.34 | 0.25 | 0.31 | 0.02 | **0.28** | 0.96 | 0.06 | 169 | 497 |  |
| $Var(O_{1})$ | 9.97 | 10.34 | 1.30 | 1.72 | 0.04 | **0.32** | 0.93 | 1.84 | 169 | 497 |  |
| $Var(O_{2})$ | 0.78 | 0.76 | 0.31 | 0.39 | -0.02 | **0.26** | 0.95 | 0.10 | 169 | 497 |  |
| $\sigma_{T1}^{2}$ | 1.59 | 1.96 | 1.34 | 1.78 | **0.23** | **0.33** | 0.96 | 1.93 | 169 | 497 |  |
| $\sigma_{T2}^{2}$ | 0.64 | 1.69 | 1.71 | 2.91 | **1.64** | **0.70** | 0.93 | 4.04 | 169 | 497 |  |
| *Note*. Absolute *peb* and *seb* values exceeding the cut-offs for at least medium bias (> 0.10) are printed in bold*. c =* index for class; $\alpha_{2c}$= intercept for the second indicator; $\delta_{Tc}$= skewness*; E_jc_* = residual variable at occasion *j*; Estimate = mean of estimates of all included replications; $IST_{2c}$ = indicator-specific trait factor*;*$\lambda_{2c}$ = trait loading*;MSE* = mean squared error;$O_{c}$= occasion-specific factor*;peb* = parameter estimation bias; Pop. = population value; reps = replications; *SD* = standard deviation of parameter estimates; *SE* = standard error; *seb* = standard error bias; *T*_c_ = latent trait variable*;*$\nu_{c}$= degrees of freedom parameter; $\mu_{Tc}$ = location parameter for the latent trait variable; $\sigma_{Tc}^{2}$ = scale parameter for the latent trait variable. | | | | | | | | | | | |

Table S 27. *Results for the Simulation Study for the Skew t Mixture Latent State-Trait Model with* $\boldsymbol{\delta}_{\boldsymbol{Tc}}\boldsymbol{=2.8}$*, 3 Occasions and 500 Observations*

| Parameter | Pop. | Estimate | *SD* | *SE* | *peb* | *seb* | 95% coverage | *MSE* | included reps | converged reps |  |
| --- | --- | --- | --- | --- | --- | --- | --- | --- | --- | --- | --- |
| $\nu_{1}$ | 5.00 | 5.27 | 0.72 | 0.71 | 0.05 | -0.02 | 0.98 | 0.60 | 265 | 497 |  |
| $\nu_{2}$ | 5.00 | 14.44 | 99.39 | 9.97 | **1.89** | **-0.90** | 0.97 | 9967.58 | 265 | 497 |  |
| $\alpha_{21}$ | -1.02 | -1.13 | 1.02 | 1.06 | **0.11** | 0.04 | 0.96 | 1.05 | 265 | 497 |  |
| $\alpha_{22}$ | 0.70 | 0.66 | 1.35 | 1.45 | -0.05 | 0.08 | 0.94 | 1.81 | 265 | 497 |  |
| Logit | 1.15 | 1.12 | 0.22 | 0.28 | -0.03 | **0.25** | 0.98 | 0.05 | 265 | 497 |  |
| $\mu_{T1}$ | 14.32 | 14.44 | 0.53 | 0.58 | 0.01 | **0.11** | 0.95 | 0.29 | 265 | 497 |  |
| $\mu_{T2}$ | 17.79 | 17.96 | 0.57 | 0.68 | 0.01 | **0.20** | 0.96 | 0.36 | 265 | 497 |  |
| $\delta_{T1}$ | 2.80 | 2.67 | 0.67 | 0.74 | -0.05 | **0.10** | 0.97 | 0.47 | 265 | 497 |  |
| $\delta_{T2}$ | 2.80 | 2.50 | 0.79 | 0.94 | **-0.11** | **0.19** | 0.97 | 0.72 | 265 | 497 |  |
| $\lambda_{21}$ | 1.05 | 1.06 | 0.06 | 0.07 | 0.01 | 0.04 | 0.95 | 0.00 | 265 | 497 |  |
| $\lambda_{22}$ | 0.94 | 0.94 | 0.07 | 0.07 | 0.00 | 0.07 | 0.98 | 0.00 | 265 | 497 |  |
| $Var(E_{11})$ | 2.30 | 2.31 | 0.25 | 0.26 | 0.00 | 0.05 | 0.94 | 0.06 | 265 | 497 |  |
| $Var(E_{12})$ | 1.06 | 1.08 | 0.24 | 0.27 | 0.02 | **0.13** | 0.97 | 0.06 | 265 | 497 |  |
| $Var(E_{21})$ | 1.42 | 1.43 | 0.12 | 0.14 | 0.01 | **0.15** | 0.92 | 0.02 | 265 | 497 |  |
| $Var(E_{22})$ | 0.64 | 0.65 | 0.13 | 0.14 | 0.02 | **0.12** | 0.97 | 0.02 | 265 | 497 |  |
| $Var({IST}_{21})$ | 0.63 | 0.62 | 0.17 | 0.18 | -0.02 | 0.06 | 0.98 | 0.03 | 265 | 497 |  |
| $Var({IST}_{22})$ | 0.33 | 0.33 | 0.18 | 0.20 | 0.00 | 0.08 | 0.97 | 0.03 | 265 | 497 |  |
| $Var(O_{1})$ | 9.97 | 10.24 | 0.91 | 1.05 | 0.03 | **0.16** | 0.96 | 0.90 | 265 | 497 |  |
| $Var(O_{2})$ | 0.78 | 0.80 | 0.25 | 0.29 | 0.03 | **0.12** | 0.97 | 0.06 | 265 | 497 |  |
| $\sigma_{T1}^{2}$ | 1.59 | 1.75 | 0.98 | 1.21 | **0.10** | **0.23** | 0.97 | 0.99 | 265 | 497 |  |
| $\sigma_{T2}^{2}$ | 0.64 | 1.33 | 1.32 | 1.69 | **1.08** | **0.28** | 0.97 | 2.22 | 265 | 497 |  |
| *Note*. Absolute *peb* and *seb* values exceeding the cut-offs for at least medium bias (> 0.10) are printed in bold*. c =* index for class; $\alpha_{2c}$= intercept for the second indicator; $\delta_{Tc}$= skewness*; E_jc_* = residual variable at occasion *j*; Estimate = mean of estimates of all included replications; $IST_{2c}$ = indicator-specific trait factor*;*$\lambda_{2c}$ = trait loading*;MSE* = mean squared error;$O_{c}$= occasion-specific factor*;peb* = parameter estimation bias; Pop. = population value; reps = replications; *SD* = standard deviation of parameter estimates; *SE* = standard error; *seb* = standard error bias; *T*_c_ = latent trait variable*;*$\nu_{c}$= degrees of freedom parameter; $\mu_{Tc}$ = location parameter for the latent trait variable; $\sigma_{Tc}^{2}$ = scale parameter for the latent trait variable. | | | | | | | | | | | |

Table S 28. *Results for the Simulation Study for the Skew t Mixture Latent State-Trait Model with* $\boldsymbol{\delta}_{\boldsymbol{Tc}}\boldsymbol{=2.8}$*, 3 Occasions and 1,000 Observations*

| Parameter | Pop. | Estimate | *SD* | *SE* | *peb* | *seb* | 95% coverage | *MSE* | included reps | converged reps |  |
| --- | --- | --- | --- | --- | --- | --- | --- | --- | --- | --- | --- |
| $\nu_{1}$ | 5.00 | 5.14 | 0.46 | 0.47 | 0.03 | 0.03 | 0.96 | 0.23 | 391 | 500 |  |
| $\nu_{2}$ | 5.00 | 5.27 | 1.84 | 1.86 | 0.05 | 0.01 | 0.96 | 3.48 | 391 | 500 |  |
| $\alpha_{21}$ | -1.02 | -1.07 | 0.63 | 0.67 | 0.05 | 0.08 | 0.96 | 0.39 | 391 | 500 |  |
| $\alpha_{22}$ | 0.70 | 0.68 | 0.89 | 0.96 | -0.03 | 0.07 | 0.97 | 0.80 | 391 | 500 |  |
| Logit | 1.15 | 1.13 | 0.16 | 0.19 | -0.02 | **0.18** | 0.97 | 0.03 | 391 | 500 |  |
| $\mu_{T1}$ | 14.32 | 14.36 | 0.36 | 0.37 | 0.00 | 0.04 | 0.96 | 0.13 | 391 | 500 |  |
| $\mu_{T2}$ | 17.79 | 17.90 | 0.38 | 0.40 | 0.01 | 0.06 | 0.95 | 0.16 | 391 | 500 |  |
| $\delta_{T1}$ | 2.80 | 2.75 | 0.45 | 0.48 | -0.02 | 0.06 | 0.96 | 0.20 | 391 | 500 |  |
| $\delta_{T2}$ | 2.80 | 2.64 | 0.54 | 0.59 | -0.06 | 0.08 | 0.98 | 0.32 | 391 | 500 |  |
| $\lambda_{21}$ | 1.05 | 1.05 | 0.04 | 0.04 | 0.00 | 0.08 | 0.95 | 0.00 | 391 | 500 |  |
| $\lambda_{22}$ | 0.94 | 0.94 | 0.04 | 0.05 | 0.00 | 0.07 | 0.96 | 0.00 | 391 | 500 |  |
| $Var(E_{11})$ | 2.30 | 2.30 | 0.18 | 0.18 | 0.00 | -0.02 | 0.96 | 0.03 | 391 | 500 |  |
| $Var(E_{12})$ | 1.06 | 1.09 | 0.17 | 0.19 | 0.03 | 0.06 | 0.95 | 0.03 | 391 | 500 |  |
| $Var(E_{21})$ | 1.42 | 1.42 | 0.09 | 0.10 | 0.00 | 0.08 | 0.91 | 0.01 | 391 | 500 |  |
| $Var(E_{22})$ | 0.64 | 0.65 | 0.09 | 0.09 | 0.01 | **0.11** | 0.95 | 0.01 | 391 | 500 |  |
| $Var({IST}_{21})$ | 0.63 | 0.63 | 0.12 | 0.13 | 0.00 | 0.01 | 0.97 | 0.02 | 391 | 500 |  |
| $Var({IST}_{22})$ | 0.33 | 0.32 | 0.12 | 0.13 | -0.02 | 0.06 | 0.96 | 0.01 | 391 | 500 |  |
| $Var(O_{1})$ | 9.97 | 10.09 | 0.57 | 0.70 | 0.01 | **0.22** | 0.95 | 0.34 | 391 | 500 |  |
| $Var(O_{2})$ | 0.78 | 0.79 | 0.17 | 0.18 | 0.01 | 0.09 | 0.95 | 0.03 | 391 | 500 |  |
| $\sigma_{T1}^{2}$ | 1.59 | 1.61 | 0.77 | 0.80 | 0.01 | 0.04 | 0.97 | 0.59 | 391 | 500 |  |
| $\sigma_{T2}^{2}$ | 0.64 | 0.99 | 0.98 | 1.18 | **0.55** | **0.21** | 0.96 | 1.08 | 391 | 500 |  |
| *Note*. Absolute *peb* and *seb* values exceeding the cut-offs for at least medium bias (> 0.10) are printed in bold*. c =* index for class; $\alpha_{2c}$= intercept for the second indicator; $\delta_{Tc}$= skewness*; E_jc_* = residual variable at occasion *j*; Estimate = mean of estimates of all included replications; $IST_{2c}$ = indicator-specific trait factor*;*$\lambda_{2c}$ = trait loading*;MSE* = mean squared error;$O_{c}$= occasion-specific factor*;peb* = parameter estimation bias; Pop. = population value; reps = replications; *SD* = standard deviation of parameter estimates; *SE* = standard error; *seb* = standard error bias; *T*_c_ = latent trait variable*;*$\nu_{c}$= degrees of freedom parameter; $\mu_{Tc}$ = location parameter for the latent trait variable; $\sigma_{Tc}^{2}$ = scale parameter for the latent trait variable. | | | | | | | | | | | |

Table S 29. *Results for the Simulation Study for the Skew t Mixture Latent State-Trait Model with* $\boldsymbol{\delta}_{\boldsymbol{Tc}}\boldsymbol{=2.8}$*, 4 Occasions and 125 Observations*

| Parameter | Pop. | Estimate | *SD* | *SE* | *peb* | *seb* | 95% coverage | *MSE* | included reps | converged reps |  |
| --- | --- | --- | --- | --- | --- | --- | --- | --- | --- | --- | --- |
| $\nu_{1}$ | 5.00 | 5.00 | 5.65 | 1.49 | 1.47 | **0.13** | -0.02 | 0.95 | 117 | 495 |  |
| $\nu_{2}$ | 5.00 | 5.00 | 65.50 | 263.64 | 123.23 | **12.10** | **-0.53** | 0.82 | 117 | 495 |  |
| $\alpha_{21}$ | -1.02 | -1.02 | -1.12 | 1.62 | 1.78 | 0.09 | 0.10 | 0.95 | 117 | 495 |  |
| $\alpha_{22}$ | 0.70 | 0.70 | 0.47 | 2.44 | 2.92 | **-0.33** | **0.20** | 0.91 | 117 | 495 |  |
| Logit | 1.15 | 1.15 | 1.08 | 0.30 | 0.41 | -0.07 | **0.38** | 0.97 | 117 | 495 |  |
| $\mu_{T1}$ | 14.32 | 14.32 | 14.72 | 0.98 | 1.04 | 0.03 | 0.07 | 0.94 | 117 | 495 |  |
| $\mu_{T2}$ | 17.79 | 17.79 | 18.67 | 1.30 | 1.59 | 0.05 | **0.22** | 0.91 | 117 | 495 |  |
| $\delta_{T1}$ | 2.80 | 2.80 | 2.34 | 1.17 | 1.27 | **-0.16** | 0.09 | 0.93 | 117 | 495 |  |
| $\delta_{T2}$ | 2.80 | 2.80 | 1.63 | 1.58 | 1.98 | **-0.42** | **0.25** | 0.97 | 117 | 495 |  |
| $\lambda_{21}$ | 1.05 | 1.05 | 1.06 | 0.10 | 0.11 | 0.01 | **0.11** | 0.95 | 117 | 495 |  |
| $\lambda_{22}$ | 0.94 | 0.94 | 0.95 | 0.12 | 0.14 | 0.01 | **0.19** | 0.97 | 117 | 495 |  |
| $Var(E_{11})$ | 2.30 | 2.30 | 2.26 | 0.45 | 0.48 | -0.02 | 0.08 | 0.95 | 117 | 495 |  |
| $Var(E_{12})$ | 1.06 | 1.06 | 1.17 | 0.52 | 0.49 | **0.10** | -0.05 | 0.96 | 117 | 495 |  |
| $Var(E_{21})$ | 1.42 | 1.42 | 1.41 | 0.19 | 0.23 | -0.01 | **0.20** | 0.95 | 117 | 495 |  |
| $Var(E_{22})$ | 0.64 | 0.64 | 0.66 | 0.20 | 0.20 | 0.02 | 0.03 | 0.92 | 117 | 495 |  |
| $Var({IST}_{21})$ | 0.63 | 0.63 | 0.58 | 0.27 | 0.29 | -0.08 | **0.11** | 0.83 | 117 | 495 |  |
| $Var({IST}_{22})$ | 0.33 | 0.33 | 0.35 | 0.28 | 0.31 | 0.07 | 0.10 | 0.97 | 117 | 495 |  |
| $Var(O_{1})$ | 9.97 | 9.97 | 10.11 | 1.50 | 1.70 | 0.01 | **0.14** | 0.93 | 117 | 495 |  |
| $Var(O_{2})$ | 0.78 | 0.78 | 0.80 | 0.34 | 0.40 | 0.02 | **0.19** | 0.95 | 117 | 495 |  |
| $\sigma_{T1}^{2}$ | 1.59 | 1.59 | 2.18 | 1.47 | 2.08 | **0.37** | **0.41** | 0.97 | 117 | 495 |  |
| $\sigma_{T2}^{2}$ | 0.64 | 0.64 | 1.81 | 1.54 | 2.09 | **1.82** | **0.35** | 0.92 | 117 | 495 |  |
| *Note*. Absolute *peb* and *seb* values exceeding the cut-offs for at least medium bias (> 0.10) are printed in bold*. c =* index for class; $\alpha_{2c}$= intercept for the second indicator; $\delta_{Tc}$= skewness*; E_jc_* = residual variable at occasion *j*; Estimate = mean of estimates of all included replications; $IST_{2c}$ = indicator-specific trait factor*;*$\lambda_{2c}$ = trait loading*;MSE* = mean squared error;$O_{c}$= occasion-specific factor*;peb* = parameter estimation bias; Pop. = population value; reps = replications; *SD* = standard deviation of parameter estimates; *SE* = standard error; *seb* = standard error bias; *T*_c_ = latent trait variable*;*$\nu_{c}$= degrees of freedom parameter; $\mu_{Tc}$ = location parameter for the latent trait variable; $\sigma_{Tc}^{2}$ = scale parameter for the latent trait variable. | | | | | | | | | | | |

Table S 30. *Results for the Simulation Study for the Skew t Mixture Latent State-Trait Model with* $\boldsymbol{\delta}_{\boldsymbol{Tc}}\boldsymbol{=2.8}$*, 4 Occasions and 250 Observations*

| Parameter | Pop. | Estimate | *SD* | *SE* | *peb* | *seb* | 95% coverage | *MSE* | included reps | converged reps |  |
| --- | --- | --- | --- | --- | --- | --- | --- | --- | --- | --- | --- |
| $\nu_{1}$ | 5.00 | 5.31 | 0.97 | 0.89 | 0.06 | -0.08 | 0.96 | 1.04 | 252 | 495 |  |
| $\nu_{2}$ | 5.00 | 11.54 | 67.97 | 10.49 | **1.31** | **-0.85** | 0.94 | 4663.14 | 252 | 495 |  |
| $\alpha_{21}$ | -1.02 | -0.98 | 1.14 | 1.16 | -0.04 | 0.02 | 0.96 | 1.30 | 252 | 495 |  |
| $\alpha_{22}$ | 0.70 | 0.60 | 1.63 | 1.82 | **-0.14** | **0.12** | 0.93 | 2.68 | 252 | 495 |  |
| Logit | 1.15 | 1.11 | 0.24 | 0.31 | -0.03 | **0.30** | 0.96 | 0.06 | 252 | 495 |  |
| $\mu_{T1}$ | 14.32 | 14.40 | 0.59 | 0.71 | 0.01 | **0.21** | 0.96 | 0.35 | 252 | 495 |  |
| $\mu_{T2}$ | 17.79 | 18.18 | 0.87 | 0.99 | 0.02 | **0.14** | 0.96 | 0.91 | 252 | 495 |  |
| $\delta_{T1}$ | 2.80 | 2.76 | 0.75 | 0.91 | -0.01 | **0.21** | 0.94 | 0.57 | 252 | 495 |  |
| $\delta_{T2}$ | 2.80 | 2.23 | 1.15 | 1.29 | **-0.20** | **0.12** | 0.95 | 1.65 | 252 | 495 |  |
| $\lambda_{21}$ | 1.05 | 1.05 | 0.07 | 0.07 | 0.00 | 0.03 | 0.95 | 0.00 | 252 | 495 |  |
| $\lambda_{22}$ | 0.94 | 0.94 | 0.08 | 0.09 | 0.00 | **0.11** | 0.98 | 0.01 | 252 | 495 |  |
| $Var(E_{11})$ | 2.30 | 2.32 | 0.33 | 0.36 | 0.01 | 0.07 | 0.92 | 0.11 | 252 | 495 |  |
| $Var(E_{12})$ | 1.06 | 1.10 | 0.33 | 0.33 | 0.04 | 0.00 | 0.95 | 0.11 | 252 | 495 |  |
| $Var(E_{21})$ | 1.42 | 1.45 | 0.15 | 0.16 | 0.02 | 0.06 | 0.96 | 0.02 | 252 | 495 |  |
| $Var(E_{22})$ | 0.64 | 0.64 | 0.13 | 0.15 | 0.01 | **0.12** | 0.95 | 0.02 | 252 | 495 |  |
| $Var({IST}_{21})$ | 0.63 | 0.61 | 0.19 | 0.20 | -0.02 | 0.06 | 0.93 | 0.04 | 252 | 495 |  |
| $Var({IST}_{22})$ | 0.33 | 0.32 | 0.16 | 0.21 | -0.04 | **0.26** | 0.95 | 0.03 | 252 | 495 |  |
| $Var(O_{1})$ | 9.97 | 10.11 | 1.08 | 1.22 | 0.01 | **0.13** | 0.94 | 1.18 | 252 | 495 |  |
| $Var(O_{2})$ | 0.78 | 0.80 | 0.24 | 0.30 | 0.03 | **0.23** | 0.96 | 0.06 | 252 | 495 |  |
| $\sigma_{T1}^{2}$ | 1.59 | 1.61 | 1.08 | 1.45 | 0.01 | **0.34** | 0.96 | 1.16 | 252 | 495 |  |
| $\sigma_{T2}^{2}$ | 0.64 | 1.62 | 1.52 | 2.10 | **1.53** | **0.38** | 0.94 | 3.28 | 252 | 495 |  |
| *Note*. Absolute *peb* and *seb* values exceeding the cut-offs for at least medium bias (> 0.10) are printed in bold*. c =* index for class; $\alpha_{2c}$= intercept for the second indicator; $\delta_{Tc}$= skewness*; E_jc_* = residual variable at occasion *j*; Estimate = mean of estimates of all included replications; $IST_{2c}$ = indicator-specific trait factor*;*$\lambda_{2c}$ = trait loading*;MSE* = mean squared error;$O_{c}$= occasion-specific factor*;peb* = parameter estimation bias; Pop. = population value; reps = replications; *SD* = standard deviation of parameter estimates; *SE* = standard error; *seb* = standard error bias; *T*_c_ = latent trait variable*;*$\nu_{c}$= degrees of freedom parameter; $\mu_{Tc}$ = location parameter for the latent trait variable; $\sigma_{Tc}^{2}$ = scale parameter for the latent trait variable. | | | | | | | | | | | |

Table S 31. *Results for the Simulation Study for the Skew t Mixture Latent State-Trait Model with* $\boldsymbol{\delta}_{\boldsymbol{Tc}}\boldsymbol{=2.8}$*, 4 Occasions and 500 Observations*

| Parameter | Pop. | Estimate | *SD* | *SE* | *peb* | *seb* | 95% coverage | *MSE* | included reps | converged reps |  |
| --- | --- | --- | --- | --- | --- | --- | --- | --- | --- | --- | --- |
| $\nu_{1}$ | 5.00 | 5.17 | 0.61 | 0.61 | 0.03 | -0.01 | 0.97 | 0.40 | 361 | 499 |  |
| $\nu_{2}$ | 5.00 | 8.42 | 50.58 | 5.38 | **0.68** | **-0.89** | 0.95 | 2570.09 | 361 | 499 |  |
| $\alpha_{21}$ | -1.02 | -1.04 | 0.72 | 0.77 | 0.02 | 0.06 | 0.95 | 0.52 | 361 | 499 |  |
| $\alpha_{22}$ | 0.70 | 0.71 | 1.06 | 1.14 | 0.01 | 0.07 | 0.93 | 1.13 | 361 | 499 |  |
| Logit | 1.15 | 1.13 | 0.17 | 0.21 | -0.02 | **0.24** | 0.96 | 0.03 | 361 | 499 |  |
| $\mu_{T1}$ | 14.32 | 14.33 | 0.38 | 0.45 | 0.00 | **0.18** | 0.94 | 0.15 | 361 | 499 |  |
| $\mu_{T2}$ | 17.79 | 17.99 | 0.54 | 0.53 | 0.01 | -0.02 | 0.95 | 0.33 | 361 | 499 |  |
| $\delta_{T1}$ | 2.80 | 2.79 | 0.50 | 0.59 | 0.00 | **0.18** | 0.95 | 0.25 | 361 | 499 |  |
| $\delta_{T2}$ | 2.80 | 2.52 | 0.74 | 0.73 | -0.10 | -0.02 | 0.96 | 0.63 | 361 | 499 |  |
| $\lambda_{21}$ | 1.05 | 1.05 | 0.04 | 0.05 | 0.00 | 0.06 | 0.97 | 0.00 | 361 | 499 |  |
| $\lambda_{22}$ | 0.94 | 0.94 | 0.05 | 0.06 | 0.00 | 0.07 | 0.97 | 0.00 | 361 | 499 |  |
| $Var(E_{11})$ | 2.30 | 2.32 | 0.23 | 0.24 | 0.01 | 0.06 | 0.94 | 0.05 | 361 | 499 |  |
| $Var(E_{12})$ | 1.06 | 1.08 | 0.22 | 0.22 | 0.02 | 0.00 | 0.96 | 0.05 | 361 | 499 |  |
| $Var(E_{21})$ | 1.42 | 1.44 | 0.10 | 0.11 | 0.01 | 0.07 | 0.93 | 0.01 | 361 | 499 |  |
| $Var(E_{22})$ | 0.64 | 0.65 | 0.09 | 0.10 | 0.01 | 0.06 | 0.96 | 0.01 | 361 | 499 |  |
| $Var({IST}_{21})$ | 0.63 | 0.62 | 0.13 | 0.13 | -0.01 | 0.06 | 0.94 | 0.02 | 361 | 499 |  |
| $Var({IST}_{22})$ | 0.33 | 0.33 | 0.12 | 0.13 | -0.01 | **0.10** | 0.96 | 0.01 | 361 | 499 |  |
| $Var(O_{1})$ | 9.97 | 10.13 | 0.75 | 0.80 | 0.02 | 0.07 | 0.94 | 0.59 | 361 | 499 |  |
| $Var(O_{2})$ | 0.78 | 0.80 | 0.17 | 0.19 | 0.02 | **0.14** | 0.97 | 0.03 | 361 | 499 |  |
| $\sigma_{T1}^{2}$ | 1.59 | 1.60 | 0.79 | 0.95 | 0.01 | **0.20** | 0.96 | 0.63 | 361 | 499 |  |
| $\sigma_{T2}^{2}$ | 0.64 | 1.14 | 1.12 | 1.34 | **0.79** | **0.19** | 0.94 | 1.51 | 361 | 499 |  |
| *Note*. Absolute *peb* and *seb* values exceeding the cut-offs for at least medium bias (> 0.10) are printed in bold*. c =* index for class; $\alpha_{2c}$= intercept for the second indicator; $\delta_{Tc}$= skewness*; E_jc_* = residual variable at occasion *j*; Estimate = mean of estimates of all included replications; $IST_{2c}$ = indicator-specific trait factor*;*$\lambda_{2c}$ = trait loading*;MSE* = mean squared error;$O_{c}$= occasion-specific factor*;peb* = parameter estimation bias; Pop. = population value; reps = replications; *SD* = standard deviation of parameter estimates; *SE* = standard error; *seb* = standard error bias; *T*_c_ = latent trait variable*;*$\nu_{c}$= degrees of freedom parameter; $\mu_{Tc}$ = location parameter for the latent trait variable; $\sigma_{Tc}^{2}$ = scale parameter for the latent trait variable. | | | | | | | | | | | |

Table S 32. *Results for the Simulation Study for the Skew t Mixture Latent State-Trait Model with* $\boldsymbol{\delta}_{\boldsymbol{Tc}}\boldsymbol{=2.8}$*, 4 Occasions and 1,000 Observations*

| Parameter | Pop. | Estimate | *SD* | *SE* | *peb* | *seb* | 95% coverage | *MSE* | included reps | converged reps |  |
| --- | --- | --- | --- | --- | --- | --- | --- | --- | --- | --- | --- |
| $\nu_{1}$ | 5.00 | 5.09 | 0.41 | 0.40 | 0.02 | -0.03 | 0.96 | 0.18 | 431 | 500 |  |
| $\nu_{2}$ | 5.00 | 5.27 | 1.38 | 1.32 | 0.05 | -0.05 | 0.98 | 1.98 | 431 | 500 |  |
| $\alpha_{21}$ | -1.02 | -1.03 | 0.49 | 0.51 | 0.01 | 0.04 | 0.95 | 0.24 | 431 | 500 |  |
| $\alpha_{22}$ | 0.70 | 0.71 | 0.71 | 0.74 | 0.02 | 0.04 | 0.94 | 0.50 | 431 | 500 |  |
| Logit | 1.15 | 1.14 | 0.12 | 0.13 | -0.01 | **0.11** | 0.95 | 0.01 | 431 | 500 |  |
| $\mu_{T1}$ | 14.32 | 14.31 | 0.28 | 0.29 | 0.00 | 0.04 | 0.96 | 0.08 | 431 | 500 |  |
| $\mu_{T2}$ | 17.79 | 17.87 | 0.30 | 0.32 | 0.00 | 0.08 | 0.95 | 0.09 | 431 | 500 |  |
| $\delta_{T1}$ | 2.80 | 2.80 | 0.37 | 0.37 | 0.00 | 0.00 | 0.95 | 0.14 | 431 | 500 |  |
| $\delta_{T2}$ | 2.80 | 2.68 | 0.41 | 0.46 | -0.04 | **0.12** | 0.95 | 0.18 | 431 | 500 |  |
| $\lambda_{21}$ | 1.05 | 1.05 | 0.03 | 0.03 | 0.00 | 0.03 | 0.95 | 0.00 | 431 | 500 |  |
| $\lambda_{22}$ | 0.94 | 0.94 | 0.04 | 0.04 | 0.00 | 0.04 | 0.94 | 0.00 | 431 | 500 |  |
| $Var(E_{11})$ | 2.30 | 2.31 | 0.17 | 0.16 | 0.00 | -0.02 | 0.95 | 0.03 | 431 | 500 |  |
| $Var(E_{12})$ | 1.06 | 1.07 | 0.15 | 0.15 | 0.01 | 0.00 | 0.95 | 0.02 | 431 | 500 |  |
| $Var(E_{21})$ | 1.42 | 1.43 | 0.07 | 0.07 | 0.00 | 0.02 | 0.93 | 0.01 | 431 | 500 |  |
| $Var(E_{22})$ | 0.64 | 0.64 | 0.06 | 0.07 | 0.01 | 0.03 | 0.95 | 0.00 | 431 | 500 |  |
| $Var({IST}_{21})$ | 0.63 | 0.63 | 0.09 | 0.09 | -0.01 | 0.04 | 0.98 | 0.01 | 431 | 500 |  |
| $Var({IST}_{22})$ | 0.33 | 0.33 | 0.09 | 0.09 | 0.01 | 0.02 | 0.97 | 0.01 | 431 | 500 |  |
| $Var(O_{1})$ | 9.97 | 10.05 | 0.53 | 0.54 | 0.01 | 0.01 | 0.96 | 0.29 | 431 | 500 |  |
| $Var(O_{2})$ | 0.78 | 0.80 | 0.12 | 0.12 | 0.02 | 0.01 | 0.96 | 0.02 | 431 | 500 |  |
| $\sigma_{T1}^{2}$ | 1.59 | 1.57 | 0.62 | 0.64 | -0.02 | 0.02 | 0.96 | 0.39 | 431 | 500 |  |
| $\sigma_{T2}^{2}$ | 0.64 | 0.91 | 0.81 | 0.90 | **0.43** | **0.12** | 0.95 | 0.73 | 431 | 500 |  |
| *Note*. Absolute *peb* and *seb* values exceeding the cut-offs for at least medium bias (> 0.10) are printed in bold*. c =* index for class; $\alpha_{2c}$= intercept for the second indicator; $\delta_{Tc}$= skewness*; E_jc_* = residual variable at occasion *j*; Estimate = mean of estimates of all included replications; $IST_{2c}$ = indicator-specific trait factor*;*$\lambda_{2c}$ = trait loading*;MSE* = mean squared error;$O_{c}$= occasion-specific factor*;peb* = parameter estimation bias; Pop. = population value; reps = replications; *SD* = standard deviation of parameter estimates; *SE* = standard error; *seb* = standard error bias; *T*_c_ = latent trait variable*;*$\nu_{c}$= degrees of freedom parameter; $\mu_{Tc}$ = location parameter for the latent trait variable; $\sigma_{Tc}^{2}$ = scale parameter for the latent trait variable. | | | | | | | | | | | |

Table S 33. *Results for the Simulation Study for the Skew t Mixture Latent State-Trait Model with* $\boldsymbol{\delta}_{\boldsymbol{Tc}}\boldsymbol{=2.8}$*, 5 Occasions and 125 Observations*

| Parameter | Pop. | Estimate | *SD* | *SE* | *peb* | *seb* | 95% coverage | *MSE* | included reps | converged reps |  |
| --- | --- | --- | --- | --- | --- | --- | --- | --- | --- | --- | --- |
| $\nu_{1}$ | 5.00 | 5.49 | 1.39 | 1.62 | 0.10 | **0.17** | 0.97 | 2.16 | 183 | 494 |  |
| $\nu_{2}$ | 5.00 | 51.69 | 282.87 | 125.07 | **9.34** | **-0.56** | 0.90 | 82198.28 | 183 | 494 |  |
| $\alpha_{21}$ | -1.02 | -1.17 | 1.29 | 1.74 | **0.15** | **0.35** | 0.95 | 1.68 | 183 | 494 |  |
| $\alpha_{22}$ | 0.70 | 0.70 | 2.07 | 5.89 | 0.00 | **1.84** | 0.93 | 4.30 | 183 | 494 |  |
| Logit | 1.15 | 1.16 | 0.30 | 0.75 | 0.00 | **1.47** | 0.96 | 0.09 | 183 | 494 |  |
| $\mu_{T1}$ | 14.32 | 14.61 | 0.74 | 1.29 | 0.02 | **0.73** | 0.96 | 0.64 | 183 | 494 |  |
| $\mu_{T2}$ | 17.79 | 18.45 | 1.30 | 1.41 | 0.04 | 0.09 | 0.92 | 2.12 | 183 | 494 |  |
| $\delta_{T1}$ | 2.80 | 2.51 | 0.91 | 1.58 | **-0.10** | **0.74** | 0.92 | 0.91 | 183 | 494 |  |
| $\delta_{T2}$ | 2.80 | 1.98 | 1.68 | 2.72 | **-0.29** | **0.62** | 0.96 | 3.49 | 183 | 494 |  |
| $\lambda_{21}$ | 1.05 | 1.06 | 0.08 | 0.10 | 0.01 | **0.24** | 0.93 | 0.01 | 183 | 494 |  |
| $\lambda_{22}$ | 0.94 | 0.94 | 0.10 | 0.28 | 0.00 | **1.77** | 0.97 | 0.01 | 183 | 494 |  |
| $Var(E_{11})$ | 2.30 | 2.32 | 0.50 | 0.79 | 0.01 | **0.57** | 0.95 | 0.25 | 183 | 494 |  |
| $Var(E_{12})$ | 1.06 | 1.12 | 0.42 | 0.53 | 0.05 | **0.27** | 0.97 | 0.18 | 183 | 494 |  |
| $Var(E_{21})$ | 1.42 | 1.46 | 0.17 | 0.29 | 0.03 | **0.66** | 0.98 | 0.03 | 183 | 494 |  |
| $Var(E_{22})$ | 0.64 | 0.64 | 0.17 | 0.20 | 0.00 | **0.21** | 0.96 | 0.03 | 183 | 494 |  |
| $Var({IST}_{21})$ | 0.63 | 0.58 | 0.24 | 0.37 | -0.08 | **0.57** | 0.90 | 0.06 | 183 | 494 |  |
| $Var({IST}_{22})$ | 0.33 | 0.33 | 0.23 | 0.49 | 0.00 | **1.16** | 0.95 | 0.05 | 183 | 494 |  |
| $Var(O_{1})$ | 9.97 | 10.18 | 1.40 | 2.72 | 0.02 | **0.94** | 0.92 | 2.01 | 183 | 494 |  |
| $Var(O_{2})$ | 0.78 | 0.78 | 0.25 | 0.49 | -0.01 | **0.96** | 0.96 | 0.06 | 183 | 494 |  |
| $\sigma_{T1}^{2}$ | 1.59 | 2.06 | 1.36 | 2.16 | **0.29** | **0.59** | 0.96 | 2.07 | 183 | 494 |  |
| $\sigma_{T2}^{2}$ | 0.64 | 1.47 | 1.48 | 6.32 | **1.29** | **3.27** | 0.92 | 2.87 | 183 | 494 |  |
| *Note*. Absolute *peb* and *seb* values exceeding the cut-offs for at least medium bias (> 0.10) are printed in bold*. c =* index for class; $\alpha_{2c}$= intercept for the second indicator; $\delta_{Tc}$= skewness*; E_jc_* = residual variable at occasion *j*; Estimate = mean of estimates of all included replications; $IST_{2c}$ = indicator-specific trait factor*;*$\lambda_{2c}$ = trait loading*;MSE* = mean squared error;$O_{c}$= occasion-specific factor*;peb* = parameter estimation bias; Pop. = population value; reps = replications; *SD* = standard deviation of parameter estimates; *SE* = standard error; *seb* = standard error bias; *T*_c_ = latent trait variable*;*$\nu_{c}$= degrees of freedom parameter; $\mu_{Tc}$ = location parameter for the latent trait variable; $\sigma_{Tc}^{2}$ = scale parameter for the latent trait variable. | | | | | | | | | | | |

Table S 34. *Results for the Simulation Study for the Skew t Mixture Latent State-Trait Model with* $\boldsymbol{\delta}_{\boldsymbol{Tc}}\boldsymbol{=2.8}$*, 5 Occasions and 250 Observations*

| Parameter | Pop. | Estimate | *SD* | *SE* | *peb* | *seb* | 95% coverage | *MSE* | included reps | converged reps |  |
| --- | --- | --- | --- | --- | --- | --- | --- | --- | --- | --- | --- |
| $\nu_{1}$ | 5.00 | 5.22 | 0.79 | 0.80 | 0.04 | 0.00 | 0.96 | 0.68 | 295 | 498 |  |
| $\nu_{2}$ | 5.00 | 9.35 | 59.24 | 8.95 | **0.87** | **-0.85** | 0.92 | 3528.46 | 295 | 498 |  |
| $\alpha_{21}$ | -1.02 | -1.17 | 0.98 | 0.97 | **0.14** | -0.01 | 0.95 | 0.97 | 295 | 498 |  |
| $\alpha_{22}$ | 0.70 | 0.74 | 1.33 | 1.41 | 0.05 | 0.06 | 0.92 | 1.76 | 295 | 498 |  |
| Logit | 1.15 | 1.12 | 0.24 | 0.24 | -0.03 | 0.02 | 0.95 | 0.06 | 295 | 498 |  |
| $\mu_{T1}$ | 14.32 | 14.46 | 0.51 | 0.59 | 0.01 | **0.16** | 0.95 | 0.28 | 295 | 498 |  |
| $\mu_{T2}$ | 17.79 | 18.08 | 0.75 | 0.64 | 0.02 | **-0.14** | 0.93 | 0.64 | 295 | 498 |  |
| $\delta_{T1}$ | 2.80 | 2.65 | 0.65 | 0.74 | -0.05 | **0.14** | 0.91 | 0.44 | 295 | 498 |  |
| $\delta_{T2}$ | 2.80 | 2.39 | 0.98 | 0.84 | **-0.15** | **-0.14** | 0.94 | 1.13 | 295 | 498 |  |
| $\lambda_{21}$ | 1.05 | 1.06 | 0.06 | 0.06 | 0.01 | -0.01 | 0.93 | 0.00 | 295 | 498 |  |
| $\lambda_{22}$ | 0.94 | 0.94 | 0.07 | 0.07 | 0.00 | 0.06 | 0.98 | 0.00 | 295 | 498 |  |
| $Var(E_{11})$ | 2.30 | 2.33 | 0.34 | 0.33 | 0.01 | -0.02 | 0.91 | 0.12 | 295 | 498 |  |
| $Var(E_{12})$ | 1.06 | 1.09 | 0.29 | 0.28 | 0.02 | -0.02 | 0.98 | 0.08 | 295 | 498 |  |
| $Var(E_{21})$ | 1.42 | 1.44 | 0.14 | 0.13 | 0.02 | -0.02 | 0.96 | 0.02 | 295 | 498 |  |
| $Var(E_{22})$ | 0.64 | 0.64 | 0.11 | 0.11 | 0.00 | 0.04 | 0.96 | 0.01 | 295 | 498 |  |
| $Var({IST}_{21})$ | 0.63 | 0.61 | 0.18 | 0.16 | -0.03 | -0.06 | 0.93 | 0.03 | 295 | 498 |  |
| $Var({IST}_{22})$ | 0.33 | 0.31 | 0.14 | 0.15 | -0.06 | 0.04 | 0.96 | 0.02 | 295 | 498 |  |
| $Var(O_{1})$ | 9.97 | 10.20 | 0.95 | 1.01 | 0.02 | 0.06 | 0.97 | 0.96 | 295 | 498 |  |
| $Var(O_{2})$ | 0.78 | 0.80 | 0.20 | 0.21 | 0.02 | 0.09 | 0.94 | 0.04 | 295 | 498 |  |
| $\sigma_{T1}^{2}$ | 1.59 | 1.75 | 1.02 | 1.17 | 0.10 | **0.15** | 0.96 | 1.06 | 295 | 498 |  |
| $\sigma_{T2}^{2}$ | 0.64 | 1.30 | 1.34 | 1.34 | **1.02** | 0.00 | 0.96 | 2.24 | 295 | 498 |  |
| *Note*. Absolute *peb* and *seb* values exceeding the cut-offs for at least medium bias (> 0.10) are printed in bold*. c =* index for class; $\alpha_{2c}$= intercept for the second indicator; $\delta_{Tc}$= skewness*; E_jc_* = residual variable at occasion *j*; Estimate = mean of estimates of all included replications; $IST_{2c}$ = indicator-specific trait factor*;*$\lambda_{2c}$ = trait loading*;MSE* = mean squared error;$O_{c}$= occasion-specific factor*;peb* = parameter estimation bias; Pop. = population value; reps = replications; *SD* = standard deviation of parameter estimates; *SE* = standard error; *seb* = standard error bias; *T*_c_ = latent trait variable*;*$\nu_{c}$= degrees of freedom parameter; $\mu_{Tc}$ = location parameter for the latent trait variable; $\sigma_{Tc}^{2}$ = scale parameter for the latent trait variable. | | | | | | | | | | | |

Table S 35. *Results for the Simulation Study for the Skew t Mixture Latent State-Trait Model with* $\boldsymbol{\delta}_{\boldsymbol{Tc}}\boldsymbol{=2.8}$*, 5 Occasions and 500 Observations*

| Parameter | Pop. | Estimate | *SD* | *SE* | *peb* | *seb* | 95% coverage | *MSE* | included reps | converged reps |  |
| --- | --- | --- | --- | --- | --- | --- | --- | --- | --- | --- | --- |
| $\nu_{1}$ | 5.00 | 5.11 | 0.54 | 0.54 | 0.02 | 0.00 | 0.98 | 0.31 | 405 | 500 |  |
| $\nu_{2}$ | 5.00 | 5.35 | 1.64 | 1.66 | 0.07 | 0.01 | 0.95 | 2.82 | 405 | 500 |  |
| $\alpha_{21}$ | -1.02 | -1.06 | 0.67 | 0.65 | 0.04 | -0.04 | 0.95 | 0.45 | 405 | 500 |  |
| $\alpha_{22}$ | 0.70 | 0.77 | 0.87 | 0.91 | **0.11** | 0.04 | 0.95 | 0.77 | 405 | 500 |  |
| Logit | 1.15 | 1.14 | 0.16 | 0.18 | -0.01 | **0.12** | 0.93 | 0.02 | 405 | 500 |  |
| $\mu_{T1}$ | 14.32 | 14.37 | 0.37 | 0.38 | 0.00 | 0.04 | 0.96 | 0.14 | 405 | 500 |  |
| $\mu_{T2}$ | 17.79 | 17.90 | 0.44 | 0.48 | 0.01 | 0.08 | 0.92 | 0.21 | 405 | 500 |  |
| $\delta_{T1}$ | 2.80 | 2.74 | 0.46 | 0.49 | -0.02 | 0.05 | 0.94 | 0.22 | 405 | 500 |  |
| $\delta_{T2}$ | 2.80 | 2.65 | 0.61 | 0.68 | -0.05 | **0.12** | 0.94 | 0.39 | 405 | 500 |  |
| $\lambda_{21}$ | 1.05 | 1.05 | 0.04 | 0.04 | 0.00 | -0.04 | 0.96 | 0.00 | 405 | 500 |  |
| $\lambda_{22}$ | 0.94 | 0.94 | 0.04 | 0.04 | 0.00 | 0.03 | 0.95 | 0.00 | 405 | 500 |  |
| $Var(E_{11})$ | 2.30 | 2.32 | 0.22 | 0.23 | 0.01 | 0.02 | 0.93 | 0.05 | 405 | 500 |  |
| $Var(E_{12})$ | 1.06 | 1.07 | 0.20 | 0.20 | 0.01 | 0.01 | 0.96 | 0.04 | 405 | 500 |  |
| $Var(E_{21})$ | 1.42 | 1.42 | 0.09 | 0.09 | 0.00 | 0.00 | 0.92 | 0.01 | 405 | 500 |  |
| $Var(E_{22})$ | 0.64 | 0.65 | 0.07 | 0.08 | 0.01 | **0.11** | 0.96 | 0.01 | 405 | 500 |  |
| $Var({IST}_{21})$ | 0.63 | 0.63 | 0.12 | 0.11 | -0.01 | -0.07 | 0.97 | 0.01 | 405 | 500 |  |
| $Var({IST}_{22})$ | 0.33 | 0.32 | 0.10 | 0.10 | -0.04 | 0.04 | 0.94 | 0.01 | 405 | 500 |  |
| $Var(O_{1})$ | 9.97 | 10.09 | 0.65 | 0.70 | 0.01 | 0.07 | 0.94 | 0.44 | 405 | 500 |  |
| $Var(O_{2})$ | 0.78 | 0.79 | 0.13 | 0.15 | 0.02 | **0.11** | 0.96 | 0.02 | 405 | 500 |  |
| $\sigma_{T1}^{2}$ | 1.59 | 1.61 | 0.76 | 0.80 | 0.02 | 0.05 | 0.94 | 0.58 | 405 | 500 |  |
| $\sigma_{T2}^{2}$ | 0.64 | 0.98 | 0.94 | 1.24 | **0.53** | **0.31** | 0.95 | 1.01 | 405 | 500 |  |
| *Note*. Absolute *peb* and *seb* values exceeding the cut-offs for at least medium bias (> 0.10) are printed in bold*. c =* index for class; $\alpha_{2c}$= intercept for the second indicator; $\delta_{Tc}$= skewness*; E_jc_* = residual variable at occasion *j*; Estimate = mean of estimates of all included replications; $IST_{2c}$ = indicator-specific trait factor*;*$\lambda_{2c}$ = trait loading*;MSE* = mean squared error;$O_{c}$= occasion-specific factor*;peb* = parameter estimation bias; Pop. = population value; reps = replications; *SD* = standard deviation of parameter estimates; *SE* = standard error; *seb* = standard error bias; *T*_c_ = latent trait variable*;*$\nu_{c}$= degrees of freedom parameter; $\mu_{Tc}$ = location parameter for the latent trait variable; $\sigma_{Tc}^{2}$ = scale parameter for the latent trait variable. | | | | | | | | | | | |

Table S 36. *Results for the Simulation Study for the Skew t Mixture Latent State-Trait Model with* $\boldsymbol{\delta}_{\boldsymbol{Tc}}\boldsymbol{=2.8}$*, 5 Occasions and 1,000 Observations*

| Parameter | Pop. | Estimate | *SD* | *SE* | *peb* | *seb* | 95% coverage | *MSE* | included reps | converged reps |  |
| --- | --- | --- | --- | --- | --- | --- | --- | --- | --- | --- | --- |
| $\nu_{1}$ | 5.00 | 5.07 | 0.36 | 0.37 | 0.01 | 0.01 | 0.97 | 0.14 | 466 | 500 |  |
| $\nu_{2}$ | 5.00 | 5.14 | 1.04 | 1.05 | 0.03 | 0.01 | 0.95 | 1.09 | 466 | 500 |  |
| $\alpha_{21}$ | -1.02 | -1.06 | 0.44 | 0.45 | 0.04 | 0.02 | 0.96 | 0.19 | 466 | 500 |  |
| $\alpha_{22}$ | 0.70 | 0.72 | 0.62 | 0.63 | 0.03 | 0.01 | 0.94 | 0.38 | 466 | 500 |  |
| Logit | 1.15 | 1.15 | 0.11 | 0.11 | -0.01 | 0.08 | 0.95 | 0.01 | 466 | 500 |  |
| $\mu_{T1}$ | 14.32 | 14.34 | 0.25 | 0.26 | 0.00 | 0.02 | 0.95 | 0.06 | 466 | 500 |  |
| $\mu_{T2}$ | 17.79 | 17.82 | 0.26 | 0.28 | 0.00 | 0.07 | 0.95 | 0.07 | 466 | 500 |  |
| $\delta_{T1}$ | 2.80 | 2.78 | 0.33 | 0.33 | -0.01 | 0.00 | 0.93 | 0.11 | 466 | 500 |  |
| $\delta_{T2}$ | 2.80 | 2.76 | 0.35 | 0.39 | -0.02 | **0.11** | 0.95 | 0.13 | 466 | 500 |  |
| $\lambda_{21}$ | 1.05 | 1.05 | 0.03 | 0.03 | 0.00 | 0.02 | 0.96 | 0.00 | 466 | 500 |  |
| $\lambda_{22}$ | 0.94 | 0.94 | 0.03 | 0.03 | 0.00 | 0.01 | 0.94 | 0.00 | 466 | 500 |  |
| $Var(E_{11})$ | 2.30 | 2.31 | 0.16 | 0.16 | 0.00 | 0.01 | 0.93 | 0.02 | 466 | 500 |  |
| $Var(E_{12})$ | 1.06 | 1.07 | 0.14 | 0.14 | 0.01 | -0.02 | 0.95 | 0.02 | 466 | 500 |  |
| $Var(E_{21})$ | 1.42 | 1.42 | 0.06 | 0.06 | 0.00 | 0.04 | 0.92 | 0.00 | 466 | 500 |  |
| $Var(E_{22})$ | 0.64 | 0.64 | 0.05 | 0.05 | 0.00 | 0.01 | 0.96 | 0.00 | 466 | 500 |  |
| $Var({IST}_{21})$ | 0.63 | 0.63 | 0.08 | 0.08 | 0.00 | 0.01 | 0.97 | 0.01 | 466 | 500 |  |
| $Var({IST}_{22})$ | 0.33 | 0.32 | 0.07 | 0.07 | -0.02 | -0.05 | 0.96 | 0.01 | 466 | 500 |  |
| $Var(O_{1})$ | 9.97 | 10.03 | 0.44 | 0.46 | 0.01 | 0.04 | 0.94 | 0.20 | 466 | 500 |  |
| $Var(O_{2})$ | 0.78 | 0.79 | 0.09 | 0.10 | 0.01 | 0.08 | 0.96 | 0.01 | 466 | 500 |  |
| $\sigma_{T1}^{2}$ | 1.59 | 1.58 | 0.56 | 0.55 | -0.01 | -0.02 | 0.95 | 0.32 | 466 | 500 |  |
| $\sigma_{T2}^{2}$ | 0.64 | 0.79 | 0.63 | 0.73 | **0.23** | **0.15** | 0.94 | 0.42 | 466 | 500 |  |
| *Note*. Absolute *peb* and *seb* values exceeding the cut-offs for at least medium bias (> 0.10) are printed in bold*. c =* index for class; $\alpha_{2c}$= intercept for the second indicator; $\delta_{Tc}$= skewness*; E_jc_* = residual variable at occasion *j*; Estimate = mean of estimates of all included replications; $IST_{2c}$ = indicator-specific trait factor*;*$\lambda_{2c}$ = trait loading*;MSE* = mean squared error;$O_{c}$= occasion-specific factor*;peb* = parameter estimation bias; Pop. = population value; reps = replications; *SD* = standard deviation of parameter estimates; *SE* = standard error; *seb* = standard error bias; *T*_c_ = latent trait variable*;*$\nu_{c}$= degrees of freedom parameter; $\mu_{Tc}$ = location parameter for the latent trait variable; $\sigma_{Tc}^{2}$ = scale parameter for the latent trait variable. | | | | | | | | | | | |

Table S 37. *Results for the Simulation Study for the Skew t Mixture Latent State-Trait Model with* $\boldsymbol{\delta}_{\boldsymbol{Tc}}\boldsymbol{=2.8}$*, 6 Occasions and 125 Observations*

| Parameter | Pop. | Estimate | *SD* | *SE* | *peb* | *seb* | 95% coverage | *MSE* | included reps | converged reps |  |
| --- | --- | --- | --- | --- | --- | --- | --- | --- | --- | --- | --- |
| $\nu_{1}$ | 5.00 | 5.35 | 1.09 | 1.07 | 0.07 | -0.02 | 0.94 | 1.32 | 214 | 496 |  |
| $\nu_{2}$ | 5.00 | 26.42 | 225.93 | 36.31 | **4.28** | **-0.84** | 0.90 | 51505.52 | 214 | 496 |  |
| $\alpha_{21}$ | -1.02 | -1.04 | 1.06 | 1.26 | 0.02 | **0.19** | 0.97 | 1.12 | 214 | 496 |  |
| $\alpha_{22}$ | 0.70 | 0.83 | 1.84 | 1.88 | **0.19** | 0.02 | 0.92 | 3.42 | 214 | 496 |  |
| Logit | 1.15 | 1.13 | 0.27 | 0.31 | -0.02 | **0.14** | 0.97 | 0.08 | 214 | 496 |  |
| $\mu_{T1}$ | 14.32 | 14.54 | 0.84 | 0.74 | 0.02 | **-0.13** | 0.96 | 0.76 | 214 | 496 |  |
| $\mu_{T2}$ | 17.79 | 18.30 | 1.11 | 1.02 | 0.03 | -0.09 | 0.96 | 1.50 | 214 | 496 |  |
| $\delta_{T1}$ | 2.80 | 2.57 | 1.01 | 0.91 | -0.08 | -0.10 | 0.85 | 1.06 | 214 | 496 |  |
| $\delta_{T2}$ | 2.80 | 2.09 | 1.49 | 1.28 | **-0.25** | **-0.14** | 0.96 | 2.74 | 214 | 496 |  |
| $\lambda_{21}$ | 1.05 | 1.05 | 0.06 | 0.08 | 0.00 | **0.18** | 0.93 | 0.00 | 214 | 496 |  |
| $\lambda_{22}$ | 0.94 | 0.93 | 0.09 | 0.09 | -0.01 | 0.02 | 0.96 | 0.01 | 214 | 496 |  |
| $Var(E_{11})$ | 2.30 | 2.38 | 0.44 | 0.46 | 0.03 | 0.04 | 0.90 | 0.20 | 214 | 496 |  |
| $Var(E_{12})$ | 1.06 | 1.10 | 0.37 | 0.39 | 0.04 | 0.05 | 0.96 | 0.14 | 214 | 496 |  |
| $Var(E_{21})$ | 1.42 | 1.45 | 0.16 | 0.17 | 0.02 | 0.07 | 0.95 | 0.03 | 214 | 496 |  |
| $Var(E_{22})$ | 0.64 | 0.66 | 0.13 | 0.15 | 0.03 | **0.13** | 0.94 | 0.02 | 214 | 496 |  |
| $Var({IST}_{21})$ | 0.63 | 0.62 | 0.18 | 0.21 | -0.01 | **0.13** | 0.90 | 0.03 | 214 | 496 |  |
| $Var({IST}_{22})$ | 0.33 | 0.30 | 0.17 | 0.18 | -0.10 | 0.06 | 0.95 | 0.03 | 214 | 496 |  |
| $Var(O_{1})$ | 9.97 | 10.24 | 1.18 | 1.26 | 0.03 | 0.07 | 0.92 | 1.46 | 214 | 496 |  |
| $Var(O_{2})$ | 0.78 | 0.77 | 0.23 | 0.26 | -0.01 | **0.11** | 0.94 | 0.05 | 214 | 496 |  |
| $\sigma_{T1}^{2}$ | 1.59 | 1.77 | 1.21 | 1.43 | **0.11** | **0.18** | 0.96 | 1.49 | 214 | 496 |  |
| $\sigma_{T2}^{2}$ | 0.64 | 1.54 | 1.61 | 1.69 | **1.41** | 0.05 | 0.92 | 3.41 | 214 | 496 |  |
| *Note*. Absolute *peb* and *seb* values exceeding the cut-offs for at least medium bias (> 0.10) are printed in bold*. c =* index for class; $\alpha_{2c}$= intercept for the second indicator; $\delta_{Tc}$= skewness*; E_jc_* = residual variable at occasion *j*; Estimate = mean of estimates of all included replications; $IST_{2c}$ = indicator-specific trait factor*;*$\lambda_{2c}$ = trait loading*;MSE* = mean squared error;$O_{c}$= occasion-specific factor*;peb* = parameter estimation bias; Pop. = population value; reps = replications; *SD* = standard deviation of parameter estimates; *SE* = standard error; *seb* = standard error bias; *T*_c_ = latent trait variable*;*$\nu_{c}$= degrees of freedom parameter; $\mu_{Tc}$ = location parameter for the latent trait variable; $\sigma_{Tc}^{2}$ = scale parameter for the latent trait variable. | | | | | | | | | | | |

Table S 38. *Results for the Simulation Study for the Skew t Mixture Latent State-Trait Model with* $\boldsymbol{\delta}_{\boldsymbol{Tc}}\boldsymbol{=2.8}$*, 6 Occasions and 250 Observations*

| Parameter | Pop. | Estimate | *SD* | *SE* | *peb* | *seb* | 95% coverage | *MSE* | included reps | converged reps |  |
| --- | --- | --- | --- | --- | --- | --- | --- | --- | --- | --- | --- |
| $\nu_{1}$ | 5.00 | 5.20 | 0.70 | 0.73 | 0.04 | 0.03 | 0.96 | 0.53 | 337 | 496 |  |
| $\nu_{2}$ | 5.00 | 6.28 | 7.38 | 3.48 | **0.26** | **-0.53** | 0.95 | 56.06 | 337 | 496 |  |
| $\alpha_{21}$ | -1.02 | -1.06 | 0.75 | 0.84 | 0.04 | **0.11** | 0.96 | 0.57 | 337 | 496 |  |
| $\alpha_{22}$ | 0.70 | 0.81 | 1.33 | 1.29 | **0.16** | -0.03 | 0.93 | 1.77 | 337 | 496 |  |
| Logit | 1.15 | 1.14 | 0.20 | 0.22 | -0.01 | **0.10** | 0.95 | 0.04 | 337 | 496 |  |
| $\mu_{T1}$ | 14.32 | 14.35 | 0.47 | 0.48 | 0.00 | 0.02 | 0.95 | 0.23 | 337 | 496 |  |
| $\mu_{T2}$ | 17.79 | 18.04 | 0.72 | 0.71 | 0.01 | -0.01 | 0.95 | 0.59 | 337 | 496 |  |
| $\delta_{T1}$ | 2.80 | 2.80 | 0.60 | 0.62 | 0.00 | 0.04 | 0.90 | 0.36 | 337 | 496 |  |
| $\delta_{T2}$ | 2.80 | 2.44 | 0.95 | 0.95 | **-0.13** | 0.00 | 0.96 | 1.03 | 337 | 496 |  |
| $\lambda_{21}$ | 1.05 | 1.05 | 0.05 | 0.05 | 0.00 | 0.09 | 0.94 | 0.00 | 337 | 496 |  |
| $\lambda_{22}$ | 0.94 | 0.93 | 0.07 | 0.06 | -0.01 | -0.03 | 0.94 | 0.00 | 337 | 496 |  |
| $Var(E_{11})$ | 2.30 | 2.34 | 0.32 | 0.32 | 0.02 | -0.01 | 0.90 | 0.10 | 337 | 496 |  |
| $Var(E_{12})$ | 1.06 | 1.07 | 0.27 | 0.27 | 0.01 | 0.01 | 0.96 | 0.07 | 337 | 496 |  |
| $Var(E_{21})$ | 1.42 | 1.42 | 0.11 | 0.12 | 0.00 | 0.04 | 0.96 | 0.01 | 337 | 496 |  |
| $Var(E_{22})$ | 0.64 | 0.65 | 0.09 | 0.10 | 0.01 | 0.06 | 0.95 | 0.01 | 337 | 496 |  |
| $Var({IST}_{21})$ | 0.63 | 0.62 | 0.13 | 0.14 | -0.01 | 0.06 | 0.96 | 0.02 | 337 | 496 |  |
| $Var({IST}_{22})$ | 0.33 | 0.32 | 0.13 | 0.13 | -0.04 | 0.05 | 0.97 | 0.02 | 337 | 496 |  |
| $Var(O_{1})$ | 9.97 | 10.09 | 0.81 | 0.87 | 0.01 | 0.07 | 0.91 | 0.68 | 337 | 496 |  |
| $Var(O_{2})$ | 0.78 | 0.79 | 0.17 | 0.18 | 0.01 | 0.08 | 0.95 | 0.03 | 337 | 496 |  |
| $\sigma_{T1}^{2}$ | 1.59 | 1.56 | 0.90 | 0.99 | -0.02 | 0.09 | 0.97 | 0.82 | 337 | 496 |  |
| $\sigma_{T2}^{2}$ | 0.64 | 1.18 | 1.17 | 1.43 | **0.85** | **0.22** | 0.92 | 1.67 | 337 | 496 |  |
| *Note*. Absolute *peb* and *seb* values exceeding the cut-offs for at least medium bias (> 0.10) are printed in bold*. c =* index for class; $\alpha_{2c}$= intercept for the second indicator; $\delta_{Tc}$= skewness*; E_jc_* = residual variable at occasion *j*; Estimate = mean of estimates of all included replications; $IST_{2c}$ = indicator-specific trait factor*;*$\lambda_{2c}$ = trait loading*;MSE* = mean squared error;$O_{c}$= occasion-specific factor*;peb* = parameter estimation bias; Pop. = population value; reps = replications; *SD* = standard deviation of parameter estimates; *SE* = standard error; *seb* = standard error bias; *T*_c_ = latent trait variable*;*$\nu_{c}$= degrees of freedom parameter; $\mu_{Tc}$ = location parameter for the latent trait variable; $\sigma_{Tc}^{2}$ = scale parameter for the latent trait variable. | | | | | | | | | | | |

Table S 39. *Results for the Simulation Study for the Skew t Mixture Latent State-Trait Model with* $\boldsymbol{\delta}_{\boldsymbol{Tc}}\boldsymbol{=2.8}$*, 6 Occasions and 500 Observations*

| Parameter | Pop. | Estimate | *SD* | *SE* | *peb* | *seb* | 95% coverage | *MSE* | included reps | converged reps |  |
| --- | --- | --- | --- | --- | --- | --- | --- | --- | --- | --- | --- |
| $\nu_{1}$ | 5.00 | 5.09 | 0.49 | 0.49 | 0.02 | 0.00 | 0.95 | 0.25 | 422 | 498 |  |
| $\nu_{2}$ | 5.00 | 5.27 | 1.46 | 1.42 | 0.05 | -0.03 | 0.96 | 2.21 | 422 | 498 |  |
| $\alpha_{21}$ | -1.02 | -1.05 | 0.54 | 0.58 | 0.03 | 0.07 | 0.96 | 0.29 | 422 | 498 |  |
| $\alpha_{22}$ | 0.70 | 0.67 | 0.84 | 0.86 | -0.05 | 0.02 | 0.95 | 0.70 | 422 | 498 |  |
| Logit | 1.15 | 1.14 | 0.14 | 0.15 | -0.01 | 0.06 | 0.96 | 0.02 | 422 | 498 |  |
| $\mu_{T1}$ | 14.32 | 14.33 | 0.34 | 0.34 | 0.00 | -0.01 | 0.94 | 0.11 | 422 | 498 |  |
| $\mu_{T2}$ | 17.79 | 17.87 | 0.39 | 0.44 | 0.00 | **0.14** | 0.96 | 0.16 | 422 | 498 |  |
| $\delta_{T1}$ | 2.80 | 2.79 | 0.43 | 0.43 | 0.00 | 0.00 | 0.93 | 0.18 | 422 | 498 |  |
| $\delta_{T2}$ | 2.80 | 2.68 | 0.52 | 0.59 | -0.04 | **0.15** | 0.96 | 0.28 | 422 | 498 |  |
| $\lambda_{21}$ | 1.05 | 1.05 | 0.03 | 0.04 | 0.00 | 0.07 | 0.96 | 0.00 | 422 | 498 |  |
| $\lambda_{22}$ | 0.94 | 0.94 | 0.04 | 0.04 | 0.00 | 0.02 | 0.94 | 0.00 | 422 | 498 |  |
| $Var(E_{11})$ | 2.30 | 2.32 | 0.21 | 0.22 | 0.01 | 0.07 | 0.91 | 0.04 | 422 | 498 |  |
| $Var(E_{12})$ | 1.06 | 1.08 | 0.19 | 0.19 | 0.02 | -0.01 | 0.95 | 0.04 | 422 | 498 |  |
| $Var(E_{21})$ | 1.42 | 1.42 | 0.08 | 0.08 | 0.00 | 0.03 | 0.95 | 0.01 | 422 | 498 |  |
| $Var(E_{22})$ | 0.64 | 0.65 | 0.07 | 0.07 | 0.01 | 0.01 | 0.96 | 0.00 | 422 | 498 |  |
| $Var({IST}_{21})$ | 0.63 | 0.63 | 0.09 | 0.10 | 0.00 | 0.05 | 0.96 | 0.01 | 422 | 498 |  |
| $Var({IST}_{22})$ | 0.33 | 0.32 | 0.09 | 0.09 | -0.02 | 0.02 | 0.96 | 0.01 | 422 | 498 |  |
| $Var(O_{1})$ | 9.97 | 10.07 | 0.56 | 0.60 | 0.01 | 0.06 | 0.95 | 0.33 | 422 | 498 |  |
| $Var(O_{2})$ | 0.78 | 0.79 | 0.12 | 0.12 | 0.02 | 0.04 | 0.95 | 0.01 | 422 | 498 |  |
| $\sigma_{T1}^{2}$ | 1.59 | 1.58 | 0.69 | 0.70 | -0.01 | 0.02 | 0.96 | 0.47 | 422 | 498 |  |
| $\sigma_{T2}^{2}$ | 0.64 | 0.92 | 0.83 | 0.93 | **0.44** | **0.11** | 0.95 | 0.77 | 422 | 498 |  |
| *Note*. Absolute *peb* and *seb* values exceeding the cut-offs for at least medium bias (> 0.10) are printed in bold*. c =* index for class; $\alpha_{2c}$= intercept for the second indicator; $\delta_{Tc}$= skewness*; E_jc_* = residual variable at occasion *j*; Estimate = mean of estimates of all included replications; $IST_{2c}$ = indicator-specific trait factor*;*$\lambda_{2c}$ = trait loading*;MSE* = mean squared error;$O_{c}$= occasion-specific factor*;peb* = parameter estimation bias; Pop. = population value; reps = replications; *SD* = standard deviation of parameter estimates; *SE* = standard error; *seb* = standard error bias; *T*_c_ = latent trait variable*;*$\nu_{c}$= degrees of freedom parameter; $\mu_{Tc}$ = location parameter for the latent trait variable; $\sigma_{Tc}^{2}$ = scale parameter for the latent trait variable. | | | | | | | | | | | |

Table S 40. *Results for the Simulation Study for the Skew t Mixture Latent State-Trait Model with* $\boldsymbol{\delta}_{\boldsymbol{Tc}}\boldsymbol{=2.8}$*, 6 Occasions and 1,000 Observations*

| Parameter | Pop. | Estimate | *SD* | *SE* | *peb* | *seb* | 95% coverage | *MSE* | included reps | converged reps |  |
| --- | --- | --- | --- | --- | --- | --- | --- | --- | --- | --- | --- |
| $\nu_{1}$ | 5.00 | 5.07 | 0.33 | 0.34 | 0.01 | 0.03 | 0.95 | 0.12 | 474 | 500 |  |
| $\nu_{2}$ | 5.00 | 5.06 | 0.84 | 0.90 | 0.01 | 0.07 | 0.96 | 0.71 | 474 | 500 |  |
| $\alpha_{21}$ | -1.02 | -1.04 | 0.39 | 0.40 | 0.02 | 0.01 | 0.95 | 0.15 | 474 | 500 |  |
| $\alpha_{22}$ | 0.70 | 0.69 | 0.58 | 0.58 | -0.02 | -0.01 | 0.95 | 0.34 | 474 | 500 |  |
| Logit | 1.15 | 1.14 | 0.09 | 0.10 | -0.01 | 0.08 | 0.96 | 0.01 | 474 | 500 |  |
| $\mu_{T1}$ | 14.32 | 14.31 | 0.23 | 0.23 | 0.00 | -0.01 | 0.96 | 0.05 | 474 | 500 |  |
| $\mu_{T2}$ | 17.79 | 17.82 | 0.23 | 0.25 | 0.00 | 0.07 | 0.97 | 0.05 | 474 | 500 |  |
| $\delta_{T1}$ | 2.80 | 2.81 | 0.28 | 0.29 | 0.00 | 0.03 | 0.93 | 0.08 | 474 | 500 |  |
| $\delta_{T2}$ | 2.80 | 2.76 | 0.32 | 0.34 | -0.02 | 0.06 | 0.95 | 0.10 | 474 | 500 |  |
| $\lambda_{21}$ | 1.05 | 1.05 | 0.02 | 0.02 | 0.00 | 0.01 | 0.95 | 0.00 | 474 | 500 |  |
| $\lambda_{22}$ | 0.94 | 0.94 | 0.03 | 0.03 | 0.00 | -0.01 | 0.93 | 0.00 | 474 | 500 |  |
| $Var(E_{11})$ | 2.30 | 2.31 | 0.15 | 0.15 | 0.01 | 0.03 | 0.91 | 0.02 | 474 | 500 |  |
| $Var(E_{12})$ | 1.06 | 1.07 | 0.13 | 0.13 | 0.01 | -0.01 | 0.96 | 0.02 | 474 | 500 |  |
| $Var(E_{21})$ | 1.42 | 1.42 | 0.05 | 0.06 | 0.00 | 0.05 | 0.94 | 0.00 | 474 | 500 |  |
| $Var(E_{22})$ | 0.64 | 0.64 | 0.05 | 0.05 | 0.00 | 0.02 | 0.96 | 0.00 | 474 | 500 |  |
| $Var({IST}_{21})$ | 0.63 | 0.63 | 0.07 | 0.07 | 0.00 | 0.05 | 0.97 | 0.00 | 474 | 500 |  |
| $Var({IST}_{22})$ | 0.33 | 0.33 | 0.06 | 0.06 | -0.01 | 0.02 | 0.95 | 0.00 | 474 | 500 |  |
| $Var(O_{1})$ | 9.97 | 10.03 | 0.40 | 0.41 | 0.01 | 0.01 | 0.94 | 0.16 | 474 | 500 |  |
| $Var(O_{2})$ | 0.78 | 0.79 | 0.08 | 0.08 | 0.02 | -0.01 | 0.96 | 0.01 | 474 | 500 |  |
| $\sigma_{T1}^{2}$ | 1.59 | 1.56 | 0.51 | 0.49 | -0.02 | -0.04 | 0.95 | 0.26 | 474 | 500 |  |
| $\sigma_{T2}^{2}$ | 0.64 | 0.75 | 0.56 | 0.56 | **0.17** | 0.00 | 0.94 | 0.32 | 474 | 500 |  |
| *Note*. Absolute *peb* and *seb* values exceeding the cut-offs for at least medium bias (> 0.10) are printed in bold*. c =* index for class; $\alpha_{2c}$= intercept for the second indicator; $\delta_{Tc}$= skewness*; E_jc_* = residual variable at occasion *j*; Estimate = mean of estimates of all included replications; $IST_{2c}$ = indicator-specific trait factor*;*$\lambda_{2c}$ = trait loading*;MSE* = mean squared error;$O_{c}$= occasion-specific factor*;peb* = parameter estimation bias; Pop. = population value; reps = replications; *SD* = standard deviation of parameter estimates; *SE* = standard error; *seb* = standard error bias; *T*_c_ = latent trait variable*;*$\nu_{c}$= degrees of freedom parameter; $\mu_{Tc}$ = location parameter for the latent trait variable; $\sigma_{Tc}^{2}$ = scale parameter for the latent trait variable. | | | | | | | | | | | |

## Supplementary Figures

**Supplementary Figure 1.** Number of parameters per condition fixed due to singularity of the information matrix (Warning message given by Mplus). Scaling of the y-axis differs between parameters. C1 *=* class 1; C2 = class 2; E_1_= residual variable for the first occasion; E_2_ = residual variable for occasions > 1; O = latent occasion-specific variable; IST = latent indicator-specific trait residual variable for the second indicator; *j* = occasions; T = latent trait variable; skew = class-specific skewness parameter for the trait factor ($\delta_{Tc})$*;* $\mu_{Tc}$ = location parameter for the latent trait variable; $\sigma_{Tc}^{2}$ = scale parameter for the latent trait variable.

**Supplementary Figure 2.** Absolute bias across conditions. (A) mean parameter estimation bias (*peb*); (B) mean standard error bias (*seb*); (C) median (MD) *peb*; (D) MD *seb*. Different colours visualise different measures of central tendency. The horizontal dashed lines represent the cut-off for medium bias (10 % < *peb/* *seb* < 30 %), the horizontal dotted lines represent the cut-off for large bias (*peb/ seb* > 30 %). Error bars reflect standard errors. *j* = number of occasions; N = sample size; skew = class-specific skewness parameter for the trait factor ($\delta_{Tc}$).

**Supplementary Figure 3.** Median bias for the logit parameter; the horizontal dashed lines represent the cut-offs for medium bias (10 % < absolute *peb/ seb* < 30 %), dotted lines represent the cut-offs for large bias (absolute *peb/ seb* > 30 %). The scaling of the y-axis differs between panels. (A) Parameter estimation bias; (B) standard error bias; *j* = number of occasions; N = sample size; *peb* = parameter estimation bias; *seb* = standard error bias; skew = class-specific skewness parameter for the trait factor ($\delta_{Tc}$).

**Supplementary Figure 4.** Median (MD) bias across conditions for parameters referring to the skew *t*-distribution. The horizontal dashed lines represent the cut-offs for medium bias (10% < absolute *peb* < 30%), the horizontal dotted lines represent the cut-offs for large bias (absolute *peb* > 30%). To visualize small values, the y-axis of $\nu_{2}$ was cut off. The scaling of the y-axis differs between parameters. Error bars reflect standard errors. $\nu_{c}$= degrees of freedom parameter for class *c*; $\delta_{Tc}$ = skewness parameter for *T_c_* for class *c;* (A) parameter estimation bias (*peb*); (B) standard error bias (*seb*).

**Supplementary Figure 5***.* Median (MD) parameter estimation bias (*peb*) across replications for the variances/ scale parameters of the latent variables. The red dashed lines represent the cut-off for medium bias (10% < absolute *peb* < 30%), dotted lines represent the cut-off for large bias (absolute *peb* > 30%). To visualize small values, the y-axis of $\sigma_{T2}^{2}$ was cut off. The scaling of the y-axis differs between parameters; *c* = index for class; $IST_{c}$ = indicator-specific trait residual factor;*j* = occasions; $O_{c}$ = occasion-specific factor; skew = class-specific skewness parameter for the trait factor ($\delta_{Tc}$); $\sigma_{Tc}^{2}$ = scale parameter for *T_c_*.

**Supplementary Figure 6***.* Median (MD) standard error bias (*seb*) across replications for the variances of the latent variables. The red dashed lines represent the cut-off for medium bias (10% < absolute *peb* < 30%), dotted lines represent the cut-off for large bias (absolute *peb* > 30%). To visualize small values, the y-axes of $\sigma_{Tc}^{2}$were cut off. The scaling of the y-axis differs between parameters; *c* = index for class; $IST_{c}$ = indicator-specific trait residual factor;*j* = occasions; $O_{c}$ = occasion-specific factor; skew = class-specific skewness parameter for the trait factor ($\delta_{Tc}$); $\sigma_{Tc}^{2}$ = scale parameter for *T_c_*.

**Sample MPlus Input for the Simulation Study**

TITLE:

Data generation for a mixture LST-Model

mixture modeling: 2 classes

specification: 3 occasions 1 Trait 1 IST

Skew = 6

MONTECARLO:

NAMES=Y11 Y21 Y12 Y22 Y13 Y23;

NREPS=500;

NOBSERVATIONS=125;

CLASSES=c(2);

GENCLASSES=c(2); ! 2 classes

seed=55719;

Repsave=All;

Save=data*.dat;

Results=results_skew_s=6_j=3_n=125.txt;

MODEL POPULATION:

%OVERALL%

! Class probability

[c#1*1.15268];

! Trait

! for first indicator lam = 1

! for second indicator in class 1 = 1.05

! for second indicator in class 2 see below

T1 BY Y11@1 Y12@1 Y13@1

Y21*1.05 Y22*1.05 Y23*1.05(lamc1);

! IST2: factor for second indicator, lam all 1 (identifiability)

IST2 BY Y21@1 Y22@1 Y23@1;

! Mean(IST2) = 0 (residual factor)

[IST2@0];

! Occasion specific factors

O1 BY Y11@1 Y21@1;

O2 BY Y12@1 Y22@1;

O3 BY Y13@1 Y23@1;

! Mean(Ok) = 0 (residual factors)

[O1@0 O2@0 O3@0];

! No covariance between Ok, T1, IST

T1 with O1@0 O2@0 O3@0;

IST2 with O1@0 O2@0 O3@0;

O1 with O2@0 O3@0;

O2 with O3@0;

T1 with IST2@0;

! Item intercepts = 0 for first indicator

[Y11@0];

[Y12@0];

[Y13@0];

! Model Skewness and degrees of freedom

{T1*6};

{df*5};

! Skewness set to zero for Ok and IST2

{O1@0};

{O2@0};

{O3@0};

{IST2@0};

%c#1%

! Item intercept = -1.02 for second indicator in class 1

[Y21*-1.02] (alpha1);

[Y22*-1.02] (alpha1);

[Y23*-1.02] (alpha1);

! Scale of T1 and IST2 in class 1

T1*1.59;

IST2*0.63;

! Location (T1) in class 1

[T1*14.32];

! Var (Ok) equal across time for class 1

O1*9.97 (varo1);

O2*9.97 (varo1);

O3*9.97 (varo1);

! Residual variances of items for class 1 equal across time

! with socratic effects

Y11*2.30 (resvar11);

Y21*2.30 (resvar11);

Y12*1.42 (resvar21);

Y22*1.42 (resvar21);

Y13*1.42 (resvar21);

Y23*1.42 (resvar21);

%c#2%

! Trait

! for first indicator lam = 1

! for second indicator in class 2 = 0.94

T1 BY Y11@1 Y12@1 Y13@1

Y21*0.94 Y22*0.94 Y23*0.94(lamc2);

! intercepts = 0.70 for second indicator in class 2

[Y21*0.70] (alpha2);

[Y22*0.70] (alpha2);

[Y23*0.70] (alpha2);

! Scale T1 and IST2 in class 2

T1*0.64;

IST2*0.33;

! Location (T1) in class 2

[T1*17.79];

! Var (Ok) equal across time for class 2

O1*0.78 (varo2);

O2*0.78 (varo2);

O3*0.78 (varo2);

! Residual variances of items for class 2 equal across time

! with socratic effects

Y11*1.06 (resvar12);

Y21*1.06 (resvar12);

Y12*0.64 (resvar22);

Y22*0.64 (resvar22);

Y13*0.64 (resvar22);

Y23*0.64 (resvar22);

ANALYSIS:

type = mixture;

estimator = MLR;

distribution = SKEWT;

MODEL:

%OVERALL%

! Class probability

[c#1*1.15268];

! Trait

! for first indicator lam = 1

! for second indicator in class 1 = 1.05

! for second indicator in class 2 see below

T1 BY Y11@1 Y12@1 Y13@1

Y21*1.05 Y22*1.05 Y23*1.05(lamc1);

! IST2: factor for second indicator, lam all 1 (identifiability)

IST2 BY Y21@1 Y22@1 Y23@1;

! Mean(IST2) = 0 (residual factor)

[IST2@0];

! Occasion specific factors, lam all 1

O1 BY Y11@1 Y21@1;

O2 BY Y12@1 Y22@1;

O3 BY Y13@1 Y23@1;

! Mean(Ok) = 0 (residual factors)

[O1@0 O2@0 O3@0];

! No covariance between Ok, T1, IST

T1 with O1@0 O2@0 O3@0;

IST2 with O1@0 O2@0 O3@0;

O1 with O2@0 O3@0;

O2 with O3@0;

T1 with IST2@0;

! Item intercepts = 0 for first indicator

[Y11@0];

[Y12@0];

[Y13@0];

! Model Skewness and degrees of freedom

{T1*6};

{df*5};

! Skewness set to zero for Ok and IST2

{O1@0};

{O2@0};

{O3@0};

{IST2@0};

%c#1%

! intercept = -1.02 for second indicator in class 1

[Y21*-1.02] (alpha1);

[Y22*-1.02] (alpha1);

[Y23*-1.02] (alpha1);

! Scale T1 and IST2 in class 1

T1*1.59;

IST2*0.63;

! Location (T1) in class 1

[T1*14.32];

! Var(Ok) equal across time for class 1

O1*9.97 (varo1);

O2*9.97 (varo1);

O3*9.97 (varo1);

! Residual variances of items for class 1 equal across time

! with socratic effects

Y11*2.30 (resvar11);

Y21*2.30 (resvar11);

Y12*1.42 (resvar21);

Y22*1.42 (resvar21);

Y13*1.42 (resvar21);

Y23*1.42 (resvar21);

%c#2%

! Traitfaktor

! for first indicator lam = 1

! for second indicator in class 2 = 0.94

T1 BY Y11@1 Y12@1 Y13@1

Y21*0.94 Y22*0.94 Y23*0.94(lamc2);

! intercepts = 0.70 for second indicator in class 2

[Y21*0.70] (alpha2);

[Y22*0.70] (alpha2);

[Y23*0.70] (alpha2);

! Scale of T1 and IST2 in class 2

T1*0.64;

IST2*0.33;

! Location (T1) in class 2

[T1*17.79];

! Var(Ok) equal across time for class 2

O1*0.78 (varo2);

O2*0.78 (varo2);

O3*0.78 (varo2);

! Residual variances of items for class 2 equal across time

! with socratic effects

Y11*1.06 (resvar12);

Y21*1.06 (resvar12);

Y12*0.64 (resvar22);

Y22*0.64 (resvar22);

Y13*0.64 (resvar22);

Y23*0.64 (resvar22);

Output: Tech1 Tech8 Tech9 Residual;
