# Supplementary figures and images for: Skew t Mixture Latent State-Trait Analysis: A Monte Carlo Simulation Study on Statistical Performance
Source: Front Psychol. 2018 Aug 2;9:1323. doi: 10.3389/fpsyg.2018.01323 (PMC6083219; doi:10.3389/fpsyg.2018.01323)

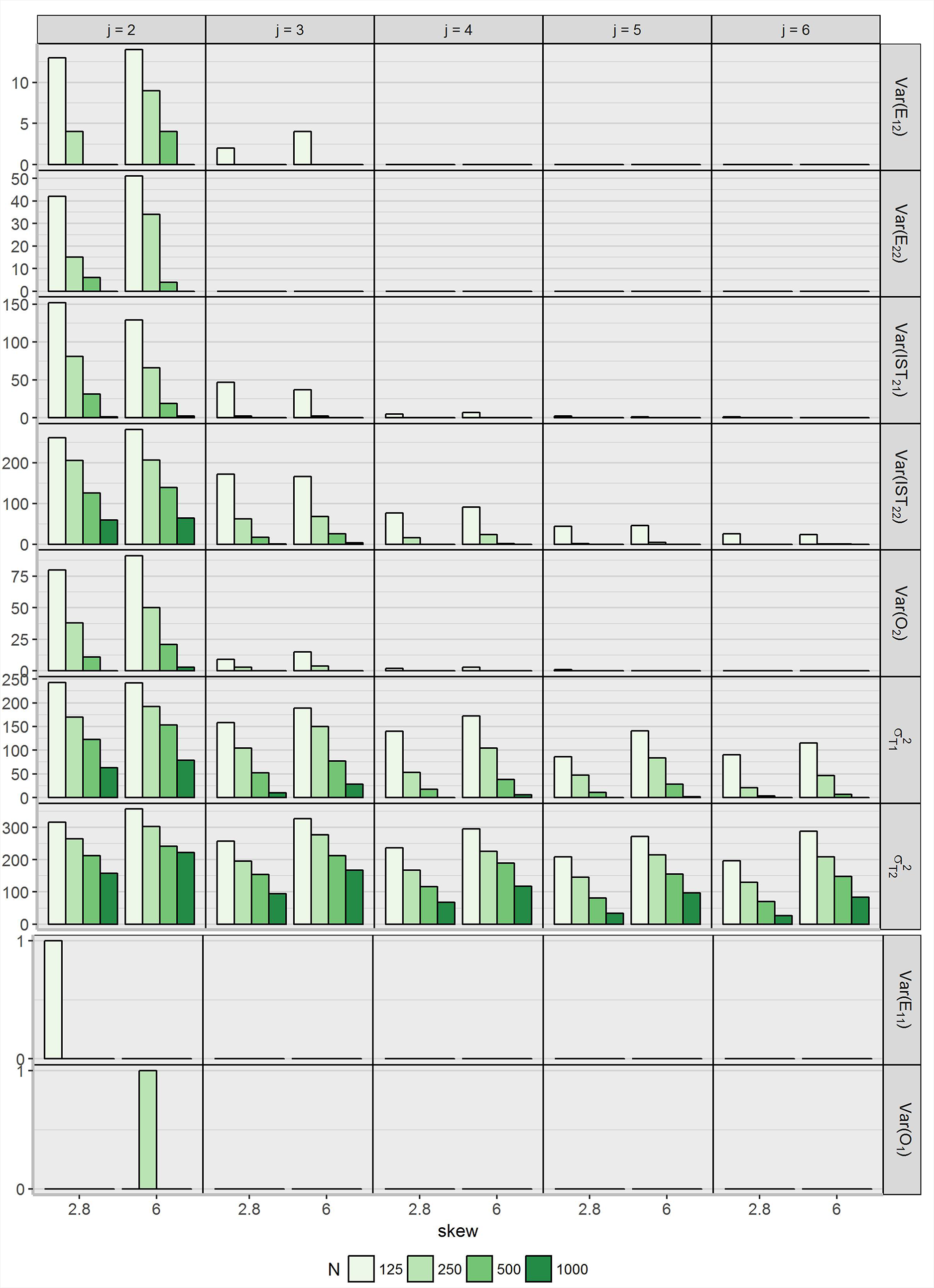

Supplement: Supplementary file 2 [file Image_1.TIF]

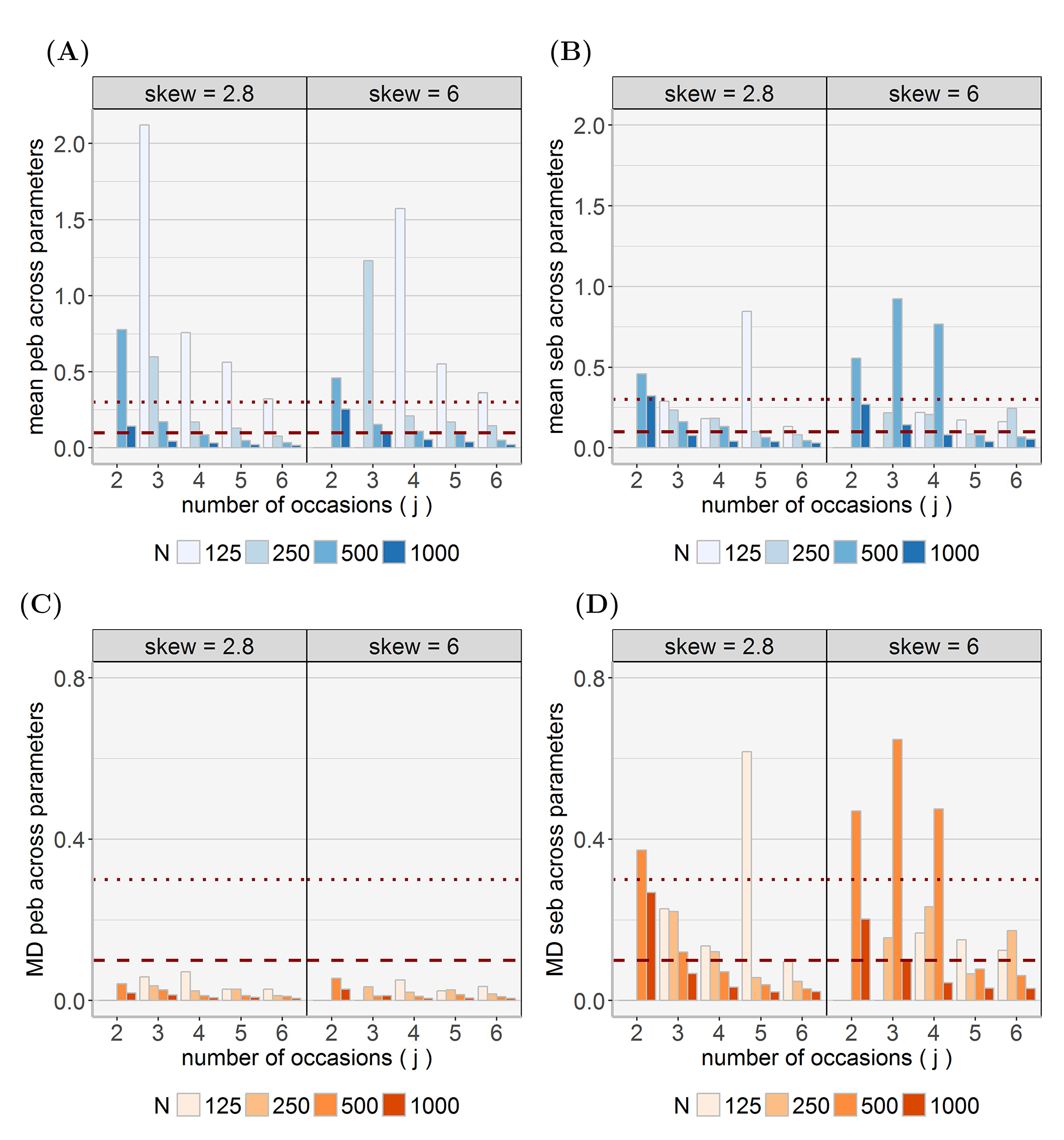

Supplement: Supplementary file 3 [file Image_2.TIF]

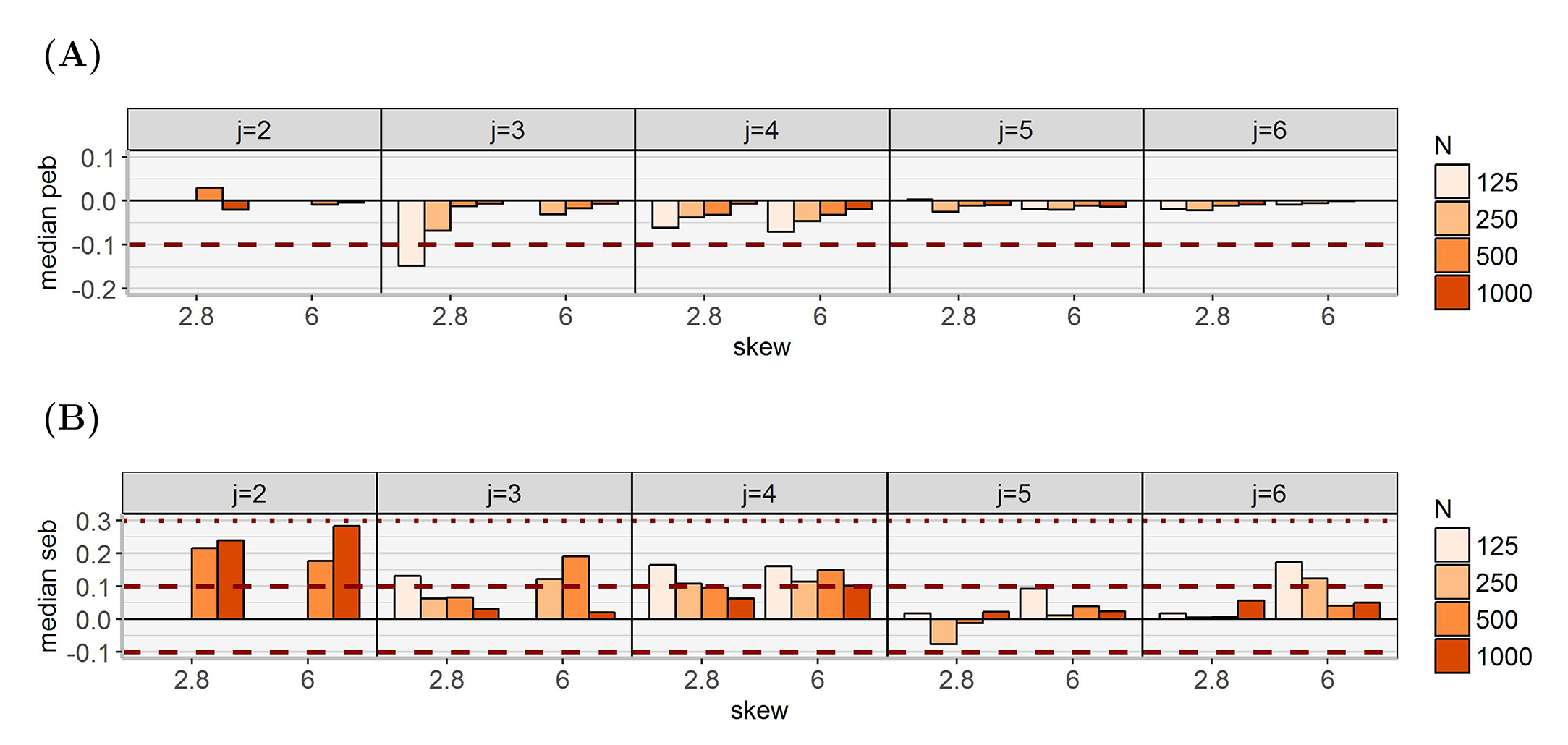

Supplement: Supplementary file 4 [file Image_3.TIF]

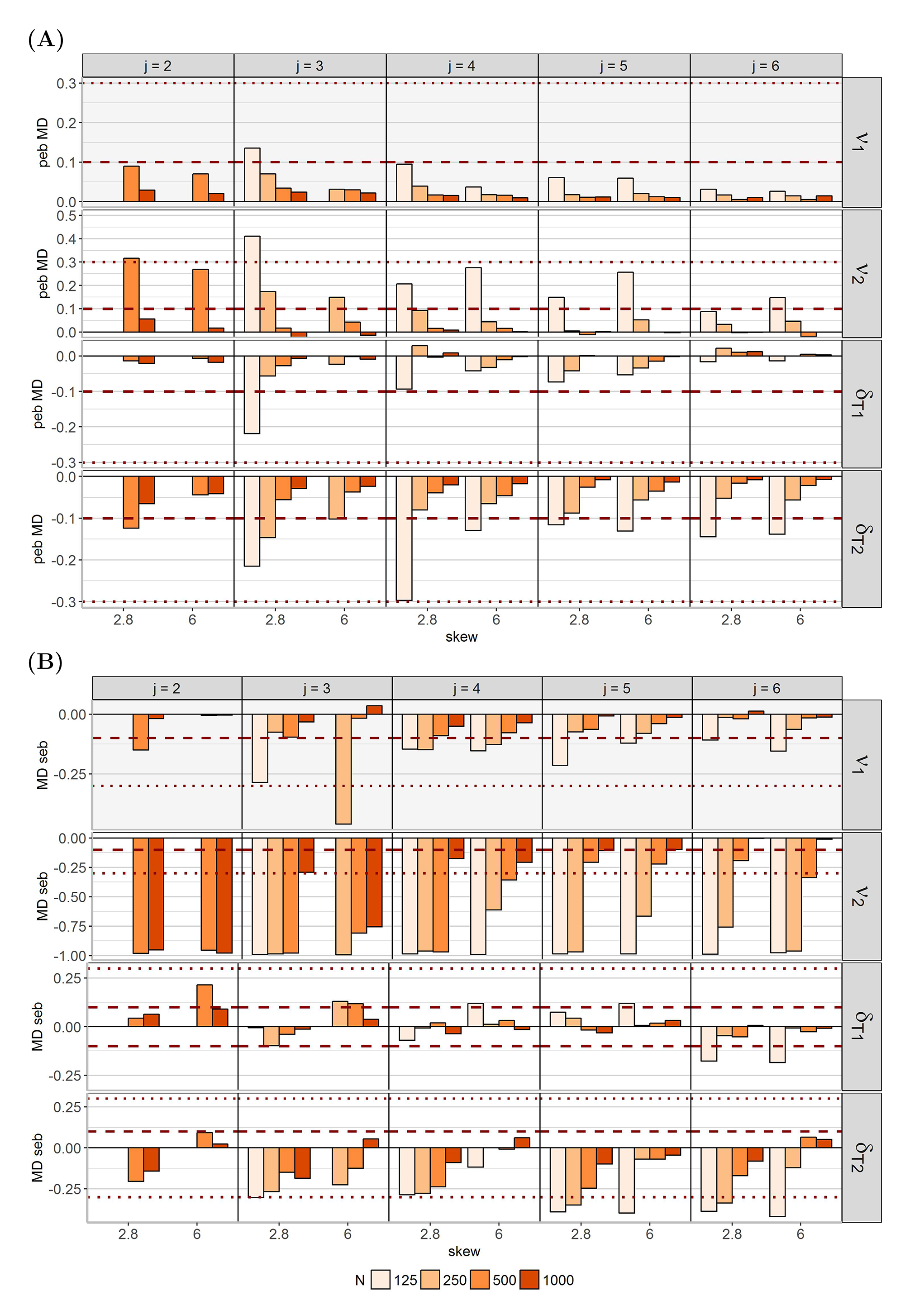

Supplement: Supplementary file 5 [file Image_4.TIF]

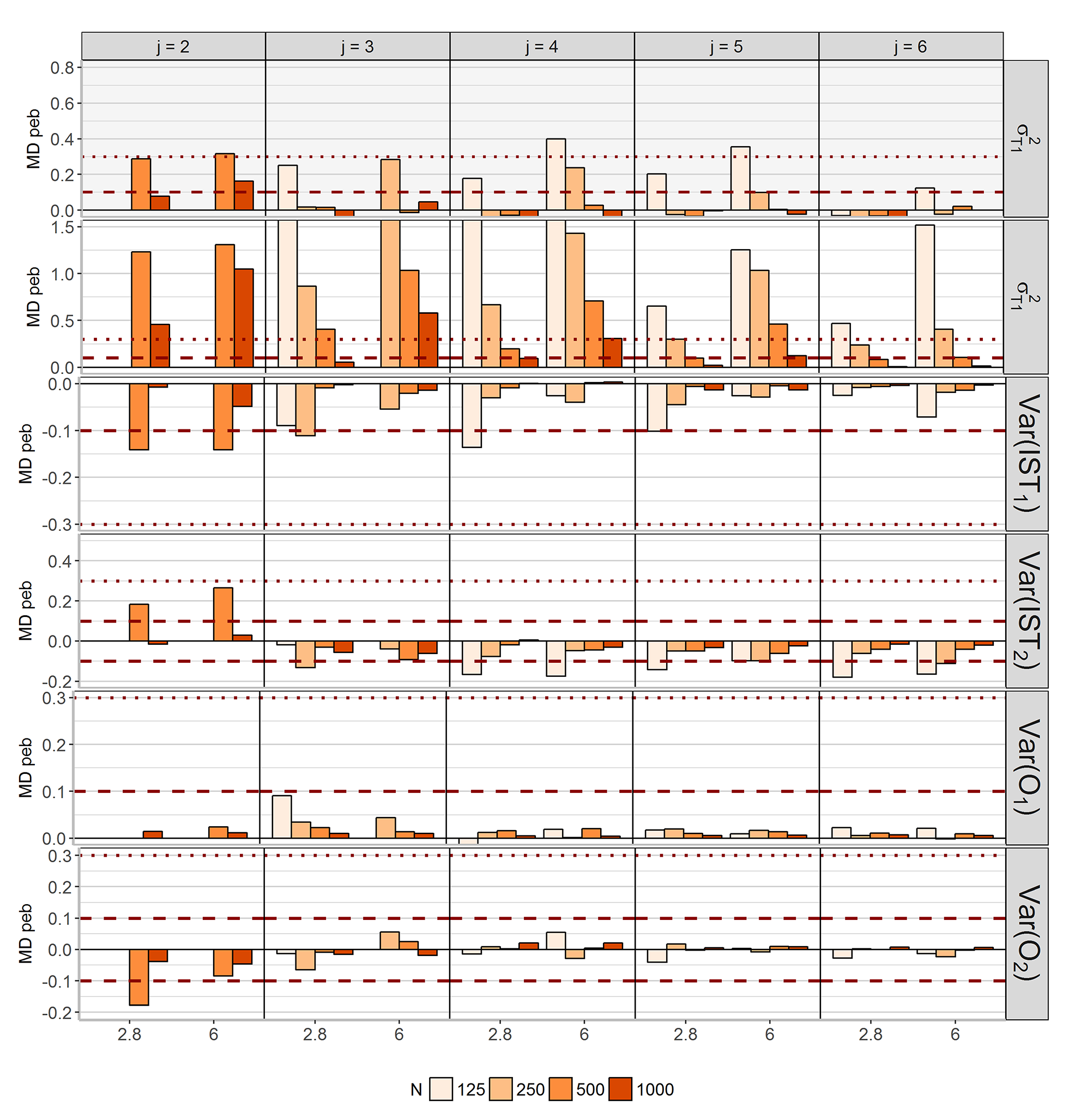

Supplement: Supplementary file 6 [file Image_5.TIF]

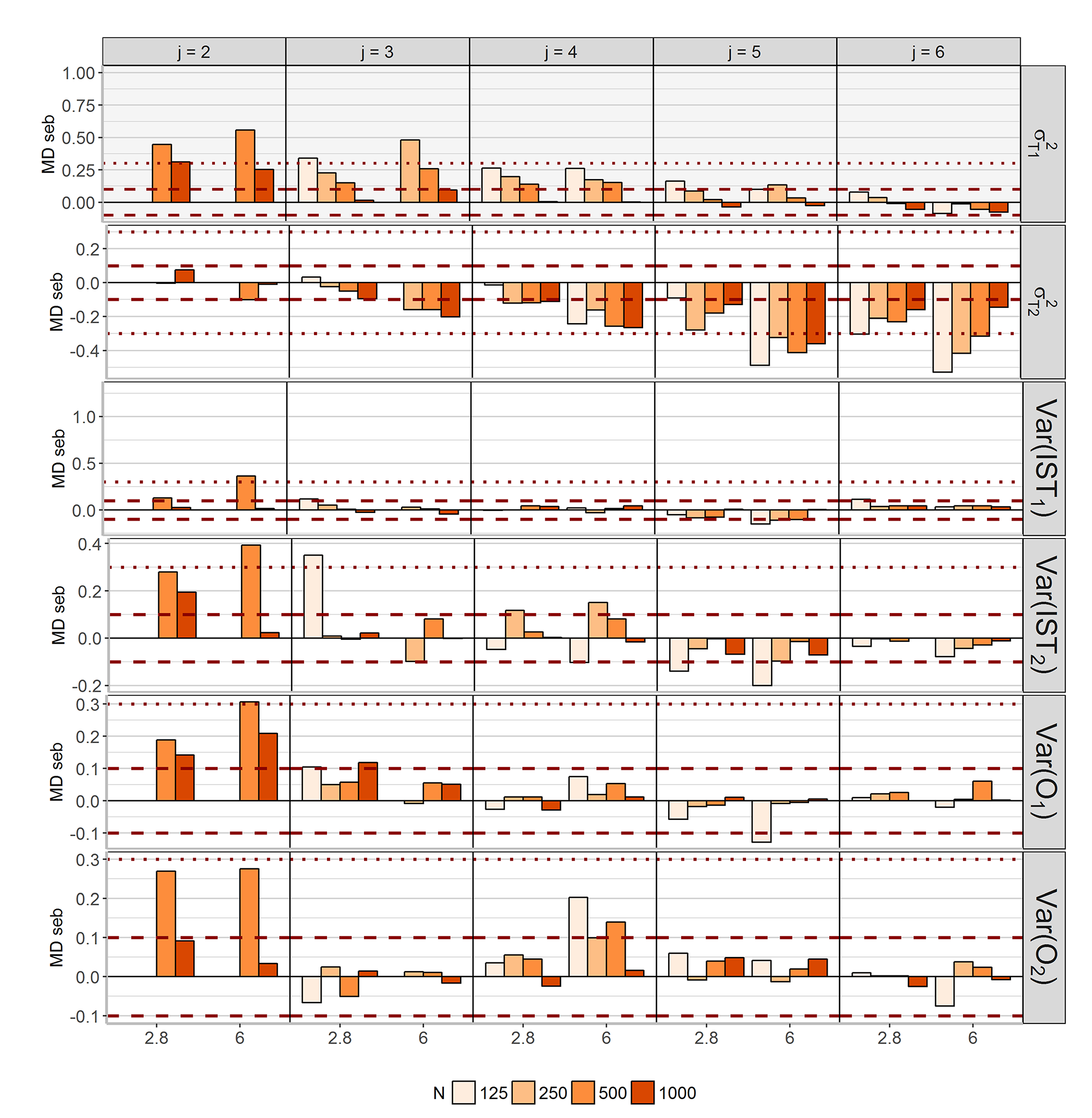

Supplement: Supplementary file 7 [file Image_6.TIF]
